# Design, Synthesis, Bioevaluation, and Bioinformatics Study of 5‐Benzylidene Hydantoin Derivatives as Novel Tyrosine Kinase Inhibitors

**DOI:** 10.1002/open.202500158

**Published:** 2025-10-09

**Authors:** Muhammad Naufal, Elvira Hermawati, Ade Danova, Ika Wiani Hidayat, Jamaludin Al‐Anshori

**Affiliations:** ^1^ Department of Chemistry, Faculty of Mathematics and Natural Sciences Universitas Padjadjaran Jl. Raya Bandung‐Sumedang km.21 Jatinangor 45363 Indonesia; ^2^ Division of Organic Chemistry Faculty of Mathematics and Natural Sciences Institut Teknologi Bandung Jl. Ganesha 10 Bandung 40132 Indonesia

**Keywords:** cancer, heterocyclic, hydantoin, tyrosine kinase

## Abstract

Tyrosine kinases regulate cellular growth, differentiation, and metabolism, and their dysregulation is implicated in malignancies, making them therapeutic targets. This study synthesizes novel 5‐benzylidene hydantoin derivatives (24–38) via benzylation and condensation, characterized by nuclear magnetic resonance (NMR), mass spectrometry, and fourier‐transform infrared (FTIR). Anticancer activity was evaluated against eight receptor tyrosine kinases at 10 μM. Six compounds—24 (34%), 25 (45%), 28 (57%), 32 (60%), 34 (49%), and 38 (56%)—show moderate HER2 inhibition (%enzyme activity ≤ 60%). Compound 38 additionally inhibits VEGFR2 (27%), PDGFRα (32%), and PDGFRβ (25%). Molecular docking reveals interactions with HER2 residues Met801, Leu726, Leu852, Phe1004, Val734, and Leu796, suggesting a structural basis for selectivity. The HER2‐targeting derivatives demonstrate potential for development as novel HER2 inhibitors. Compound 38's multikinase inhibition resembles sunitinib, a clinically approved drug for renal cell carcinoma and gastrointestinal stromal tumors, highlighting its promise for broader kinase‐targeted therapy. These findings underscore the therapeutic relevance of the 5‐benzylidene hydantoin scaffold, warranting further optimization to enhance potency and selectivity against HER2 and other oncogenic kinases.

## Graphical abstract

A series of 5‐benzylidene hydantoin derivatives is synthesized and evaluated against eight receptor tyrosine kinases (RTKs). Several compounds show selective and moderate inhibition of human epidermal growth factor receptor 2 (HER2). Molecular docking reveals key interactions with the HER2 active site, highlighting the potential of 5‐benzylidene hydantoins as anticancer lead compounds.

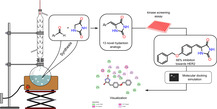

## 1 Introduction

Cancer is one of the most lethal diseases worldwide. GLOBOCAN estimates that around 20 million people are diagnosed with cancer, and 9.7 million deaths are recorded in 2022.^[^ ^1^ ^]^ Receptor tyrosine kinases (RTKs) have garnered significant attention as therapeutic targets due to their involvement in numerous cellular dysregulations, including cancer‐associated dysregulations.^[^ ^2^, ^3^, ^4^ ^]^ For example, overexpression of HER2 is seen as the leading cause of breast and ovarian cancer, and this leads to the alteration of downstream signaling pathways. These pathways are activated by homodimerization of HER2, heterodimerization with EGFR, and HER3.^[^ ^5^ ^]^ Thus, small‐molecule tyrosine kinase inhibitors (SMTKIs) have emerged as a prominent class of targeted cancer therapeutics. Nonetheless, the development of TKIs with optimal potency, selectivity, and minimal off‐target activity remains a significant challenge.^[^ ^6^ ^]^

**Figure 1:**
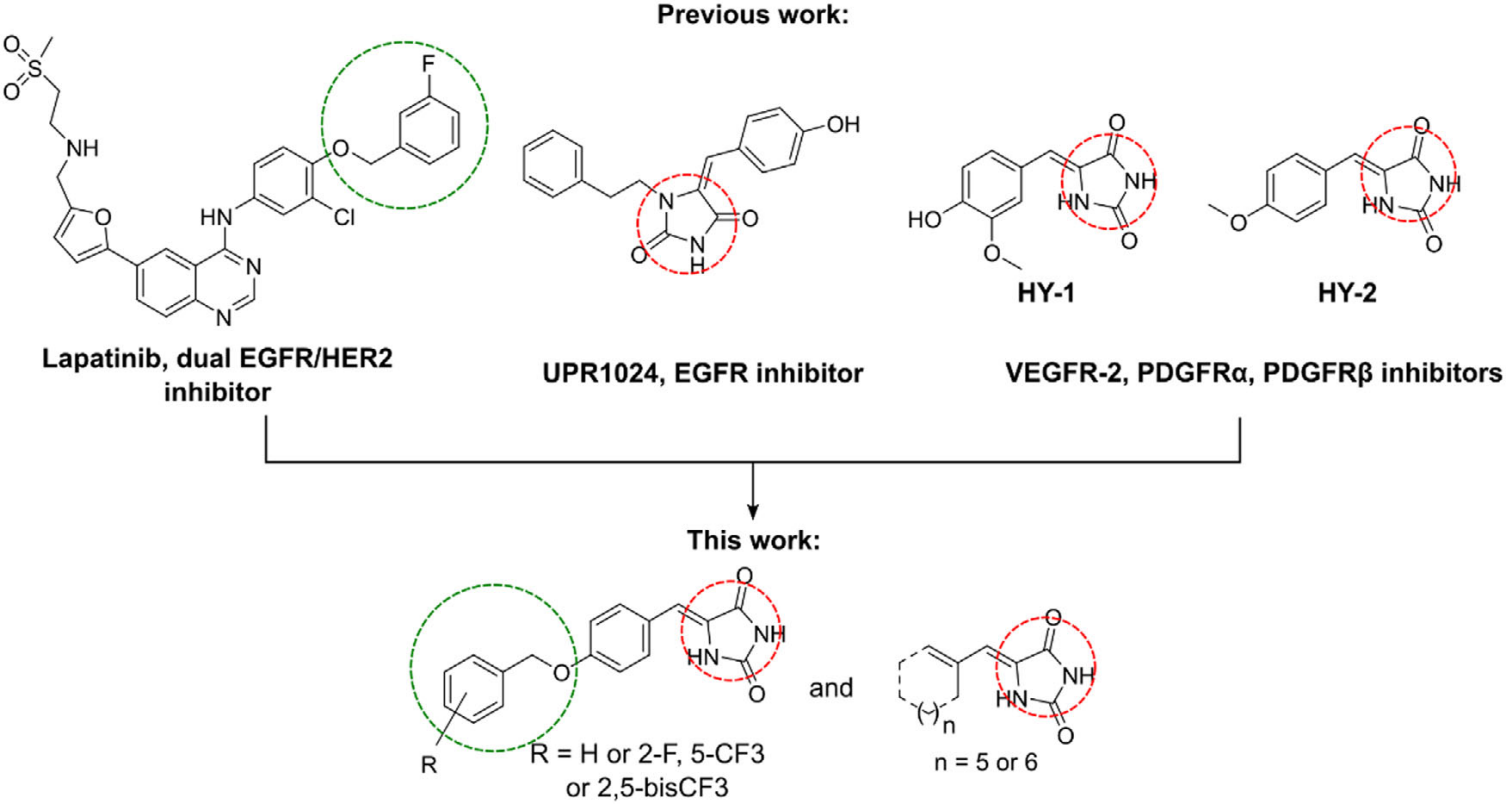
The reported tyrosine kinase inhibitors and designed novel 5‐benzylidene hydantoin derivatives.

Emerging strategies prioritize heterocyclic scaffolds that mimic the adenine‐binding motif of ATP, a hallmark of kinase inhibitor pharmacophores.^[^ ^7^ ^]^ 5‐Benzylidene hydantoin, a five‐membered diimide heterocycle, represents an underexplored candidate for TKI development. 5‐Benzylidene hydantoins have demonstrated various significant ranges of biological activity, including anticonvulsant,^[^ ^8^ ^]^ antidiabetic,^[^ ^9^ ^]^ and anticancer.^[^ ^10^ ^]^ In fact, 5‐benzylidene hydantoin derivative (UPR1024, **Figure** **1**) has been reported to inhibit EGFR autophosphorylation and induce DNA damage.^[^ ^11^ ^]^ Moreover, a recent study showed that 5‐benzylidene hydantoin (HY‐1 and HY‐2) derivatives also exhibited moderate activity toward VEGFR‐2, PDGFR*α*, and PDGFRβ.^[^ ^12^ ^]^ In this study, hydantoin derivative skeletons are constructed of 1) a hydantoin ring as an adenine mimic, as a continuous endeavor to explore hydantoin chemistry and its biological properties.^[^ ^13^, ^14^, ^15^ ^]^ A modeling study showed that the hydantoin ring formed a hydrogen bond with the hinge region.^[^ ^16^ ^]^ Next is the 2) benzylidenes as *α*,*β*‐unsaturated fragment. This moiety is a vital pharmacophore for cytotoxicity^[^ ^17^, ^18^ ^]^ since it acts as an acceptor toward biological thiol nucleophiles through Michael addition.^[^ ^19^, ^20^ ^]^ Hence, *α*,*β*‐unsaturated carbonyls are often applied to the irreversible inhibitor skeleton. Third is 3) trifluoro and fluoro benzyl group (Figure 1). The reason is that Lapatinib and SYR127063 are HER2 inhibitors that utilize fluorine size to fill the enzyme hydrophobic pocket and explain their selectivity.^[^ ^21^, ^22^, ^23^ ^]^ Furthermore, fluorine has been incorporated into hydantoin structures as the backbone of nonsteroidal antiandrogen drugs such as nilutamide, enzalutamide, and apalutamide.^[^ ^24^ ^]^ Moreover, 340 fluoro‐pharmaceutical registered drugs exist, and around 9% are antitumor agents.^[^ ^25^ ^]^

This study leverages hydantoin's inherent adaptability to address critical gaps in TKI design. Novel synthesized 5‐benzylidene hydantoins’ inhibition capability was tested against eight RTKs using a luminescent‐based assay. In addition, their binding interactions are studied through molecular docking to provide new insights into the anticancer activity of 5‐benzylidene hydantoin‐based tyrosine kinase inhibitors. This work positions 5‐benzylidene hydantoin as a versatile scaffold for next‐generation TKIs, offering a roadmap to a new class of TKIs while preserving synthetic accessibility.

## 2 Results and Discussion

### 2.1 Chemistry

**Scheme 1:**
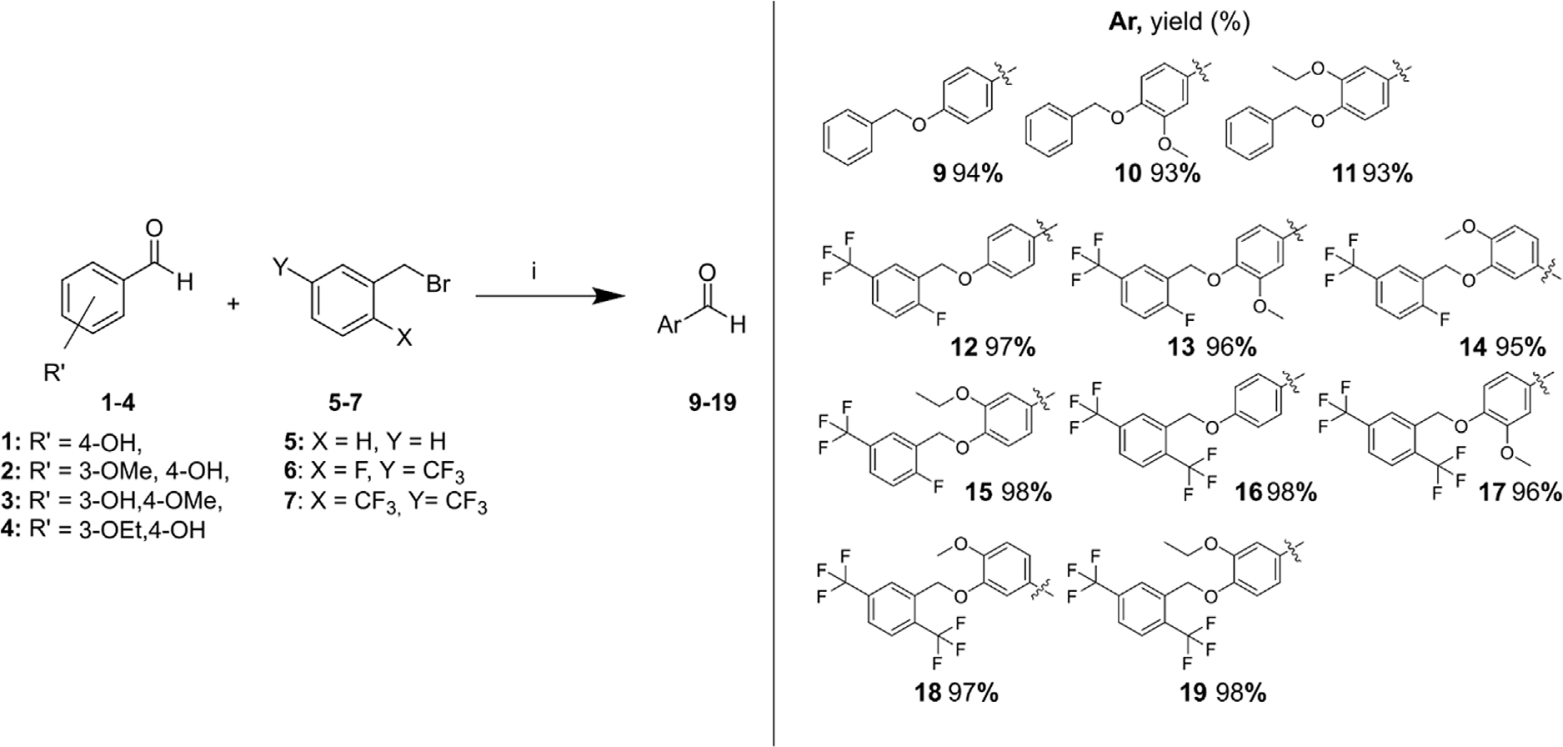
Synthesis of **9–19** (i: K_2_CO_3_, acetonitrile, reflux, 3–5 h).

Initially, benzylated aldehydes were produced by alkylation of commercially available benzaldehydes **1–4** with appropriate commercial benzyl bromides **5**–**7** in refluxing acetonitrile with potassium carbonate present (**Scheme** **1**), followed by quenching with water. Fluorinated benzaldehydes (**12**–**19**) were directly filtered and washed with hexane since they were directly precipitated. Meanwhile, **9**–**11** were extracted with ethyl acetate and evaporated to form solids. The desired products were formed in excellent yields (93%–98%). All benzylated aldehydes **9**–**19** were confirmed using ^1^H‐NMR and ^13^C‐NMR (Figure S1–S22, Supporting Information). The oxygenated methylene's proton (Ar–CH_2_–O–Ar) signals were recorded as singlets around 5.23–5.54 ppm with the integration of 2H, while their carbons were found in a range of 63.44–71.25 ppm.

**Scheme 2:**
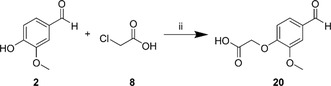
Synthesis of **20** (ii: NaOH, water, reflux, 6 h).

Using NaOH as a base in water, vanillin **2** and chloroacetic acid **8** were added to create 2‐(4‐formyl‐2‐methoxyphenoxy)acetic acid **20** (**Scheme** **2**), which were then worked up in an acidic medium and washed with hexane. The ^1^H‐NMR of **20** showed a singlet at 4.89 ppm (2H), which is attributable to the HCOO‐CH_2_‐O‐Ar resonance. In ^13^C‐NMR, the carbon atom of the same methylene group resonated at 64.98 ppm (Figure S23–S24, Supporting Information).

**Scheme 3:**
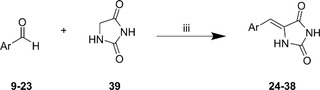
Synthesis of **24–38** (NH_4_OAc, AcOH, reflux, 5–7 h).

**Figure 2:**
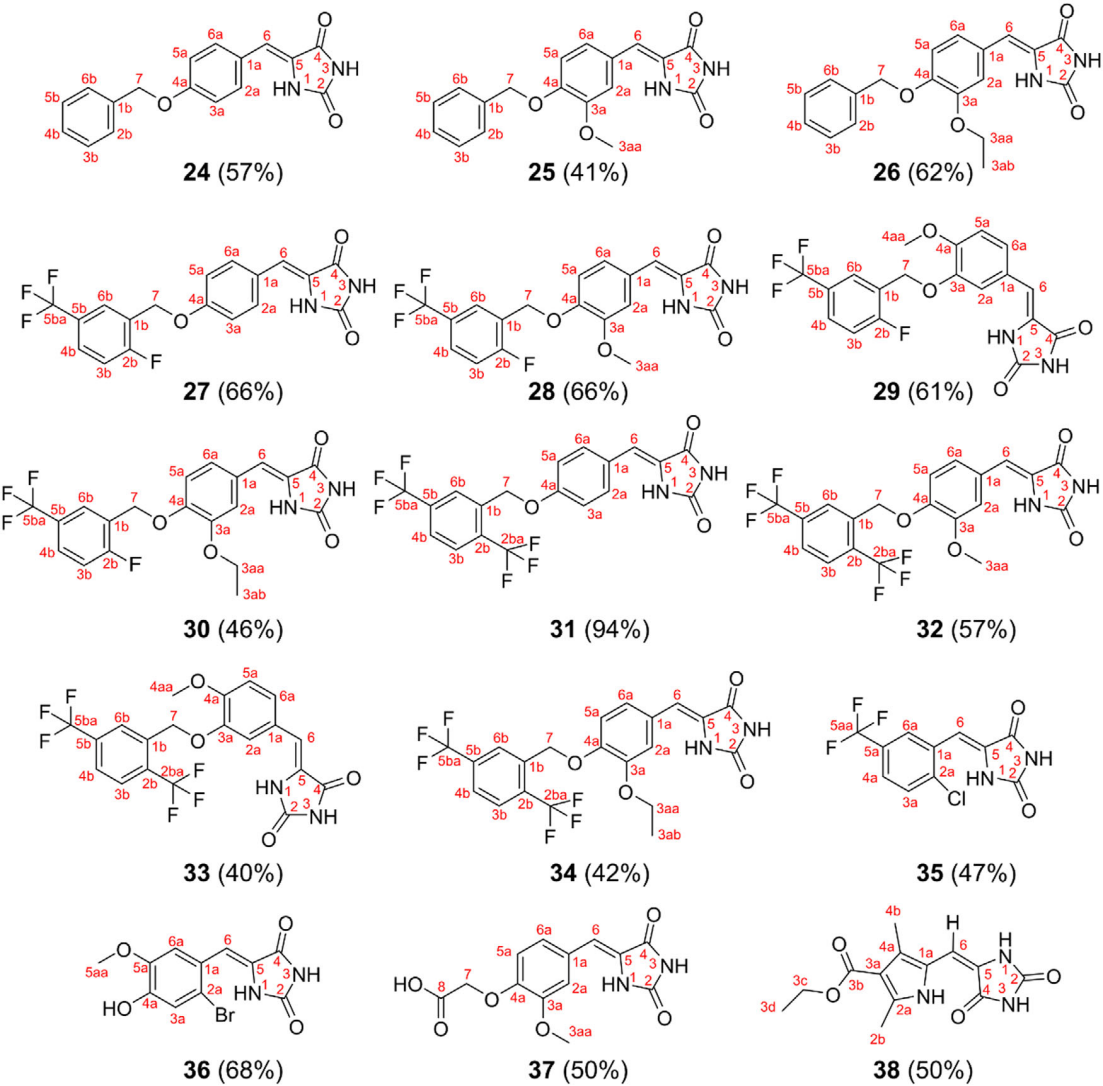
Structure of compounds **24–38**, the number in parentheses is the yield.

All benzylated benzaldehydes **9**‐**19**, alkylated benzaldehyde **20,** and commercial aldehydes **21**‐**23** served as precursors for condensation with hydantoin **39**. Condensations were performed using the NH_4_OAc/AcOH system at reflux temperature, as already reported before^[^ ^26^ ^]^ (**Scheme** **3**). The 5‐benzylidene hydantoins **24**‐**37** and pyrrolmethylene hydantoin **38** were obtained in a modest yield of around 27%–94%. These synthesized compounds were confirmed with ^1^H‐NMR, ^13^C‐NMR, ^19^F‐NMR, mass spectrometry, and infrared. In ^1^H‐NMR, vinylogous protons of **24**‐**38** resonated as singlets in the range of 6.31–6.47 ppm. At the same time, these vinylogous carbons were identified in the range of 97.39–109.11 ppm. These shifts indicate that all synthesized **24**–**38** are formed in the *Z* isomer,^[^ ^27^ ^]^ except **38** is an *E*‐isomer (**Figure** **2**). Amides of hydantoins were shifted to a more downfield area around 10–11 ppm. In ^1^H‐NMR, the splitting coupling constant of di‐substituted and tri‐substituted arylidene protons were also identified around *J* = 7–8 Hz except for compounds **36** and **38** (Figure S25–S82, Supporting Information). In mass spectrometry, the mass of all target compounds **24**–**38** was consistent with calculated mass data (*m/z* deviation ≤ 0.0005 ppm). Brominated **35** and chlorinated **36** derivatives showed a characteristic peak to their corresponding isotope (Figures S70 and S74, Supporting Information). Infrared spectroscopy showed that N–H stretches of targeted compounds are found in 3100–3400 cm^−1^. Their C=O stretches are found in the 1710–1780 cm^−1^ range, while their corresponding C=C stretches are around 1600 cm^−1^ (Figure S25–S82, Supporting Information).

### 2.2 Biological Activity

Compounds **24**–**38** were screened against the kinase panel containing 8 different RTKs that consist of EGFR, HER2, HER3, IGF1R, InsR, VEGFR‐2, PDGFRα, and PDGFRβ, and erlotinib served as a positive control. Five benzylated analogs showed moderate and selective activity towards HER‐2 (**Table** **1**). Unsubstituted benzyloxy (**24**) showed the most potent inhibition with 34%enzyme activity, while 3‐ethoxy benzyloxy (**26**, 101%enzyme activity) had the lowest inhibition of all tested compounds. In the 2‐fluoro‐5‐CF_3_ benzyloxy series (Table 1), the 3‐methoxy analog **28** has the highest activity with 57%enzyme activity. The addition of 2‐F and 5‐CF_3_ in **27** significantly decreased the activity (90%enzyme activity) compared to its counterpart **24**. The 2‐fluoro‐5‐CF_3_ benzyloxy in *meta* position (compound **29**) resulted in lower activity than its isomer **28**. Meanwhile, the 3‐ethoxy 2‐fluoro‐5‐CF_3_ derivative **30** (78%enzyme activity) showed higher inhibition than its counterpart **26** (101%enzyme activity).

**Table 1:** Tyrosine kinase activity of compounds **24–38** at concentration 10 μM.

Compounds/tyrosine kinase	**24**	**25**	**26**	**27**	**28**	**29**	**30**	**31**	**32**	**33**	**34**	**35**	**36**	**37**	**38**	Erlotinibb)
	%enzyme activitya)															
EGFR	100	90	104	103	103	88	91	104	88	88	65	107	101	101	98	0
HER2	34	45	101	90	57	90	78	67	60	66	49	80	96	93	56	41
HER4	90	73	102	96	96	99	97	101	102	98	97	90	81	99	88	38
IGF1R	100	78	100	100	94	97	98	94	83	89	87	107	96	99	83	97
InsR	101	101	107	99	104	100	104	93	104	104	103	98	97	96	102	42
VEGFR‐2	83	75	95	89	96	100	98	101	98	94	93	107	91	103	27	6
PDGFRα	86	84	82	83	88	88	91	84	85	91	86	94	65	88	32	26
PDGFRβ	86	79	108	89	101	102	103	91	96	96	94	95	68	97	25	16

a) >60%activity = weak, 20%–60% activity = moderate, <20% activity = strong;

b) at concentration 1 μM.

In the 2,5‐bisCF_3_ derivatives (**31**–**34**, Table 1), the 3‐ethoxy‐2,5‐bisCF_3_ derivative **34** (49%enzyme activity) exhibited the best activity in comparison with other 2,5‐bisCF_3_ derivatives (compounds **31–33**). The unsubstituted 2,5‐bisCF_3_ analogs **31** recorded a better activity (67%enzyme activity) rather than 2‐fluro‐5‐CF_3_ derivatives **27** (90%enzyme activity). Unfortunately, the HER2 activity **31** is still recorded twice as high as **27**. The 3‐methoxy‐2,5‐bisCF_3_ analog **32** had slightly lower activity than its counterpart **28** (60 vs. 57%enzyme activity) and significantly lower than **25** (45%enzyme activity). In contrast, the bis‐CF_3_ benzyloxy group in the *meta* position (compound **33**, 66%enzyme activity) increased the activity compared to its *meta*‐2‐fluoro‐5‐CF_3_ derivatives (**29**, 90%enzyme activity). The disappearance of the benzyloxy group in **35–37** (Table 1) resulted in a significant loss of activity towards HER‐2 (80, 94, and 93%enzyme activity, respectively). This stressed the importance of the benzyloxy group in the 5‐benzylidene hydantoin structure to inhibit HER2 activity. Interestingly, **38** recorded moderate activity toward HER‐2 with 56%enzyme activity. In addition, this compound also showed vigorous activity toward VEGFR‐2 (27%enzyme activity), PDGFRα (32%enzyme activity), and PDGFRβ (25%enzyme activity).

In general, compound **24** is more potent than the known RTK inhibitor erlotinib (34%enzyme activity vs. 41%enzyme activity). In addition, **25** and **34** also have slightly similar inhibition to erlotinib. Screening results suggest that 3‐methoxy derivatives (**25**, **28**, and **32**) have a consistently moderate HER2 inhibitory activity regardless of their benzyloxy substituents. These trends, while preliminary, provide a roadmap for prioritizing substituents in future hydantoin‐based TKIs. This inhibition assay prioritized derivatives showing ≤60% activity at 10 μM, a threshold used in early‐stage TKI discovery.^[^ ^28^, ^29^, ^30^ ^]^ These compounds were flagged as leads for downstream validation since overexpression of HER2 is seen as the leading cause of breast and ovarian cancer, leading to the alteration of downstream signaling pathways.^[^ ^5^ ^]^ Moreover, VEGFR‐2 dimerization also led to the activation of downstream signaling pathways such as Akt, mTOR, Erk1/2, and FAK. These signals promote angiogenesis for cancer cell survival.^[^ ^31^ ^]^

### 2.3 Molecular Docking

Molecular docking is performed to study the interaction of those moderately active compounds (**24**, **25**, **28**, **32**, **34**, and **38**) with HER‐2. All compounds are docked in the ATP binding site of HER2. In this study, the crystal structure is obtained from the protein data bank with PDB ID: 3PP0. The receptor is a dimer with chain A and chain B. Both chains are complexed with a strong and selective HER2 inhibitor SYR127063 (IC_50_ = 11 nM).^[^ ^21^ ^]^ This inhibitor then docked onto chain B since this chain has more complete residues than chain A, with the additional Pro999 to Leu1009. Particularly, Phe1004 resides near the adenine binding site. Validation from redocking of SYR127063 toward chain B resulted in an root mean square deviation (RMSD) = 0.32 Å with a binding affinity of −14.21 kcal mol^−1^. Since chain B contains missing residues, homology modeling is also performed using MODELLER. On a more complete model, the RMSD of redocking is obtained at 0.78 Å, with SYR127063 is −11.64 kcal mol^−1^. Furthermore, molecular docking is also validated in various scoring functions such as vinardo,^[^ ^32^ ^]^ smina,^[^ ^33^ ^]^ and AutoDock4.^[^ ^34^ ^]^ RMSD of those three scoring functions are 0.77, 0.77, and 1.00 Å, respectively, with each SYR127063's binding energy is measured at −6.70, −11.70, and −6.70 kcal mol^−1^. All postures had RMSD values below the criterion of 2.0 Å.^[^ ^35^ ^]^ Thus the redocking protocol is also applied to all moderately active synthesized derivatives and erlotinib. In all scoring functions, synthesized derivatives (**24**, **25**, **28**, **32**, and **34**) exhibit lower binding affinity than erlotinib. SYR127063 has the lowest binding affinity, and **38** has the highest binding affinity (**Table** **2**). While no universally standardized thresholds exist, several studies showed that ligands with vina scores ≤−7.5 kcal mol^−1^ were classified as potential HER2 inhibitors.^[^ ^36^, ^37^ ^]^

**Table 2:** Molecular docking binding affinity data from various scoring functions (missres = missing residues, AD4 = AutoDock4).

Compound	%Enzyme activity	Vina missres	Vina complete	Vinardo	AD4	Smina
		Binding affinity [kcal mol^−1^]				
**24**	34	−9.41	−9.29	−6.96	−8.07	−9.61
**25**	45	−10.22	−9.62	−6.80	−8.50	−9.53
**28**	57	−10.66	−10.51	−7.41	−8.61	−10.33
**32**	60	−10.86	−10.58	−7.50	−8.87	−10.35
**34**	49	−11.33	−10.45	−7.28	−8.83	−10.42
**38**	56	−7.83	−7.98	−5.43	−7.71	−8.06
Erlotinib	41	−8.74	−8.61	−5.21	−8.45	−8.10
SYR127063	–	−14.21	−11.64	−6.70	−9.26	−11.70
R^2^	–	0.06	0.11	0.06	0.07	0.07
RMSD of SYR127063	–	0.32	0.78	0.77	1.00	0.77

However, the correlation between docking‐calculated binding affinity and %enzyme activity by the kinase assay method used in this research resulted in a low coefficient of determination (r^2^ ≤  0.11). Several factors led to the low r^2^ value, including the following: 1) Kinase assay in this research is designed as initial high‐throughput derivatives screening or profiling; thus, more detailed approaches such as IC_50_ measurement or more direct measurement of thermodynamic parameters such as isothermal titration calorimetry (ITC) are required for further experiments.^[^ ^38^ ^]^ 2) Since molecular docking is frequently used as a virtual screening tool on big datasets of chemicals, it is designed to be robust and employs sampling techniques on rigid protein structures to reduce the time and expense of computational calculations. However, molecular docking is a reliable tool for visually inspecting the observed binding resemblance with ligand‐receptor structure complex and their inter/intramolecular interactions.^[^ ^39^ ^]^

**Figure 3:**
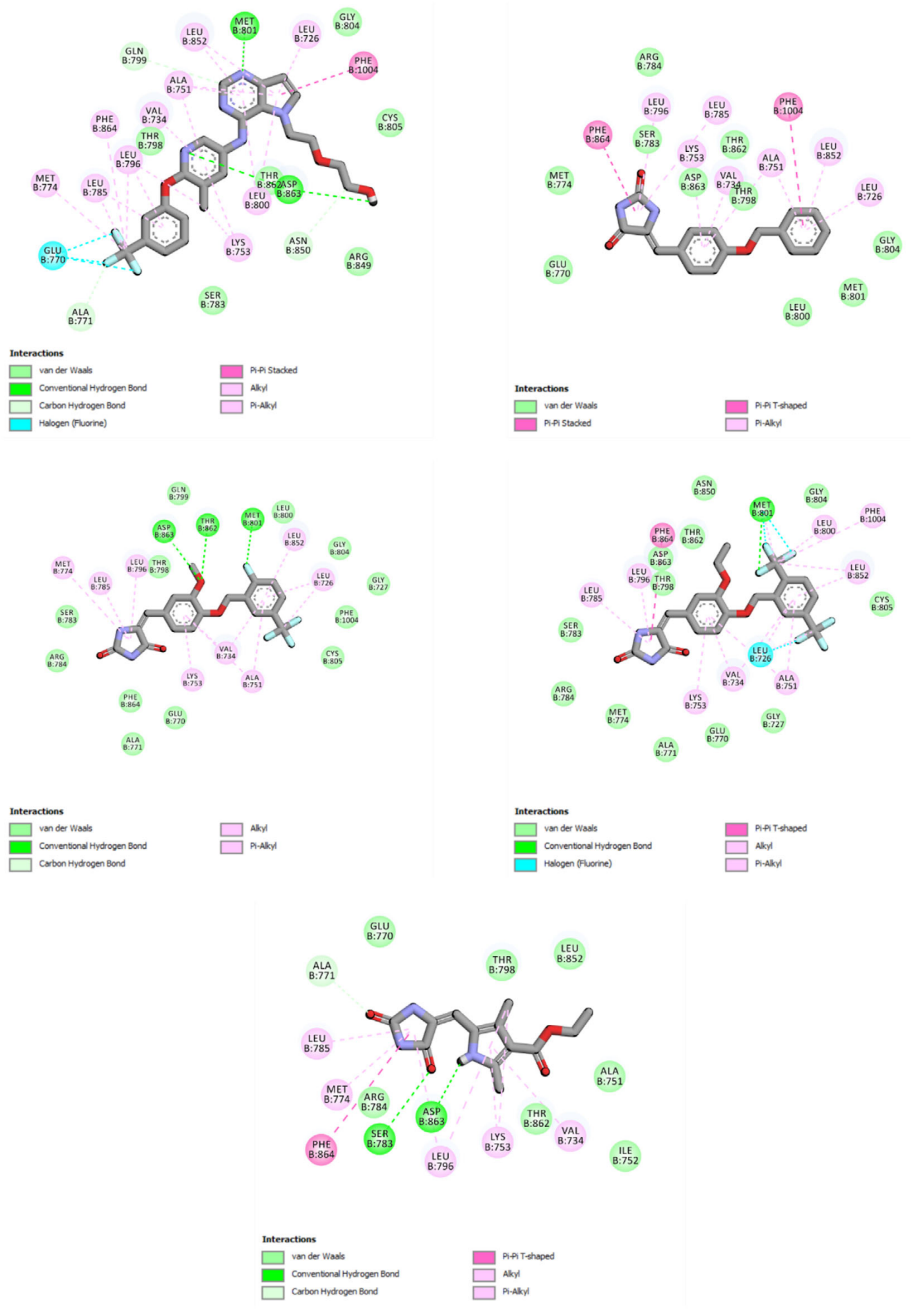
Molecular docking interactions of SYR127063, **24**, **28**, **34**, and **38** in the HER2 ATP binding pocket.

Based on molecular docking analysis, we present several important interactions between inhibitors in the adenine binding site of HER2. The pyrrolo[3,2‐d]pyrimidine ring of SYR127063 formed a hydrogen bond with Met801 of HER2 in the hinge region. For ATP competitive inhibitors, hydrogen bond interaction with the kinase hinge is usually crucial for potent inhibition. In chain B, this ring formed π–π stacking with Phe1004. Nonbonded π interaction also formed between pyrrolo[3,2‐d]pyrimidine and several hydrophobic residues such as Leu726, Leu852, Ala751, and Leu800. The ethoxyethane‐1‐ol side chain is oriented toward the solvent‐exposed area and forms a hydrogen bond with Asp863 of the conserved DFG loop. The pyridine ring of SYR127063 formed a π‐alkyl with Val734. The trifluoromethyl benzene moiety occupies a hydrophobic region composed of Leu796, Leu785, and Phe864. At the same time, its trifluoromethyl created halogen interaction with Glu770 and Met774 of the α‐helix in the allosteric region (**Figure** **3**).

The hydantoin ring of **24** is occupied in a hydrophobic region composed of Phe864 (T‐shaped π‐π interaction) and Leu796 (π‐alkyl interaction). The arylidene moiety of **24** formed a nonbonded π interaction with Val734, Lys753, and Ala751. Meanwhile, **24**'s benzyloxy is occupied in the adenine binding site. The ring notably formed π–π stacked interaction with Phe1004 and occupied the hydrophobic pocket composed of Ala751, Leu726, and Leu852. Compound **28** was also oriented nearly in the same manner as compound **24** (Figure 3). Hydantoin resided in the hydrophobic pocket composed of Leu796, Leu785, and Met774. Its arylidene moiety made a nonbonded π interaction with Val734, Lys753, and Ala751. The methoxy group apparently formed additional hydrogen bonding with Asp863 (DFG loop) and Thr862. The 2‐fluoro‐5‐CF_3_ benzyloxy moiety occupied the hydrophobic pocket of the adenine site and created nonbonded π interactions with Leu852, Leu726, and Ala751. It is also noted that the 2‐fluorine atom formed hydrogen bonding with Met801 of the kinase hinge region.

The bisCF_3_ analog (**34**) resembles compounds **24** and **28** (Figure 3). The hydantoin ring resided in the hydrophobic region, creating non‐bonded π interaction Phe864 (T‐shaped π–π), Leu785, and Leu796. The benzyloxy occupied a hydrophobic pocket and created interactions with Val734, Lys753, and Ala751. The 2,5‐BisCF_3_ benzyloxy resided in the hydrophobic pocket of the adenine binding site and made several non‐bonded π interactions with Phe1004, Leu852, Leu800, and Ala 751. The 2‐CF_3_ created multiple interactions with Met801 (hydrogen bond and halogen interactions), while 5‐CF_3_ created a halogen interaction with Leu726.

Hydantoin of **38** also resided in a hydrophobic pocket near the allosteric site (Figure 3). It created several nonbonded π interactions with Phe864 (T‐shaped π–π), Leu785, and Met774. Hydantoin's carbonyl also formed a hydrogen bond with Ser783. The 2,4‐dimethyl‐1 H‐pyrrole moiety formed a hydrogen bond with Asp863 of the DFG loop and created a hydrophobic interaction with Val734 and Lys753. In this simulation, this compound did not exhibit any nonbonded interaction in the hydrophobic pocket of the adenine site since, without the elongated benzyloxy moiety, the size of compound **38** was smaller than the other three and the reference compound.

## 3 Conclusion

In conclusion, the targeted compound can be synthesized using Knoevenagel condensation and O‐alkylation. The benzaldehyde derivatives were successfully synthesized from the benzylation and alkylation reactions with excellent yields of >90%. 13 new derivatives of 5‐aryldine hydantoin **26**–**38** have been obtained with yields ranging from 27% to 94%. Compounds **24**, **25**, **28**, **32,** and **34** showed moderate and selective activity towards HER2, with %enzyme activity in the 34%–60% range. Moreover, compound **38** had moderate activity towards HER2, VEGFR‐2, PDGFRα, and PDGFRβ, with each %enzyme activity being 56%, 27%, 32%, and 25%, respectively. The interactions can be classified into four areas. In the hinge region, hydrogen bonding to Met801 is the essential interaction. In hydrophobic region I, Leu726, Leu852, and Phe1004 are the most interacted residues with ligands. Val734 and Phe864 are the most interacted residues in hydrophobic region II. Leu796 is the most interacted residue in hydrophobic region III with ligands. Despite its limitations, this work positions hydantoin as a versatile scaffold for overcoming TKI resistance. By integrating enzymatic screening with computational rigor, we identified key substituents for optimizing binding and selectivity. Our findings underscore the value of hybrid methodologies in early drug discovery, particularly for resource‐constrained teams. Future efforts will focus on experimental validation to translate these insights into clinically viable candidates.

## 4 Experimental Section

4.1

4.1.1

##### Materials and Methods

Benzaldehyde derivatives were synthesized in parallel using the Radleys Carousel 12 Plus Reaction Station. Moreover, 5‐benzylidene hydantoin derivatives were synthesized in conventional glass laboratory equipment at reflux temperature. Kinase selectivity profiling system (KSPS) RTKs TK1 (EGFR, HER2, HER4, IGF1R, InsR, KDR, PDGFRα, and PDGFRβ) were purchased from Promega. The ADP‐Glo Kinase assay was obtained from Promega. Kinase enzyme reaction setup, including compound dilution and preparation of kinase working stock, ATP/substrate working stock, ADP‐Glo addition, and kinase detection reagent, were carried out using a Pipetmax‐268 automatic liquid handler from Gilson. Automated KSPS protocols for Pipetmax were imported into Trilution micro 2.0 software (Gilson). Luminescence in the enzyme assay was measured with a Promega GloMax Explorer GM3510, while data processing was performed using the SMART protocol present in the GloMax Explorer software. The molecular docking calculation was performed on an Intel Core i5‐6300U@2.4 GHz.

All precursors of synthesis, hydantoin, aldehydes (4‐hydroxybenzaldehyde, 4‐hydroxy‐3‐methoxybenzaldehyde, 3‐hydroxy‐4‐methoxybenzaldehyde, 3‐ethoxy‐4‐hydroxybenzaldehyde, 2‐bromo‐4‐hydroxy‐5‐methoxybenzaldehyde, 2‐chloro‐5‐(trifluoromethyl)benzaldehyde, ethyl 5‐formyl‐2,4‐dimethyl‐1H‐pyrrole‐3‐carboxylate), benzyl bromides, and chloroacetic acid were obtained from the Merck–Sigma Aldrich and Alfa Chem. All reagents and solvents were of analytical grade and used without further purification. Thin‐layer chromatography GF_254_ was obtained from Merck. Erlotinib was purchased from Merck (SML2156). The receptor structure for molecular docking was obtained from the Protein Data Bank (https://www.rcsb.org/).

##### General Procedures for Benzylation

Compounds **1**–**4** (1 mmol), benzyl bromide **5–7** (1.2 mmol), and potassium carbonate (2 mmol) were dissolved in 6 mL of acetonitrile. The reaction mixture was stirred at reflux temperature for 4–5 h. Thin layer chromatography (TLC) monitored the progress of the reaction, and upon completion, the reaction mixture was quenched with water, extracted with ethyl acetate, and washed with brine. The organic layer was dried over anhydrous sodium sulfate, filtered, and concentrated under reduced pressure. The crude products were washed with hexane to remove the rest of the benzyl bromide. Products **9–19** were characterized using ^1^H‐NMR and ^13^C‐NMR (see Figure S7–S22, Supporting Information).

##### General Procedures for Alkylation

Compound **2** (1 mmol), chloroacetic acid **8** (2 mmol), and NaOH (3 mmol) were reacted under reflux conditions in water for 6 h.^[^ ^40^ ^]^ The reaction mixture was stirred at reflux temperature for 4–5 h. TLC monitored the reaction's progress, and upon completion, the mixture was acidified with a concentrated 37% HCl. The precipitation was filtered and washed with cold water and hexane. Product **20** was characterized by using ^1^H‐NMR and ^13^C‐NMR (Figure S23 and S24, Supporting Information).

##### General Procedures for Condensation

Hydantoin **39** (0.5 mmol), benzaldehydes **9–23** (0.55 mmol), and ammonium acetate (0.5 mmol) were dissolved in 1.5 mL of acetic acid. The reaction mixture was stirred at reflux temperature for 5–6 h. TLC monitored the progress of the reaction, and upon completion, the reaction mixture was quenched with water. The reaction mixture was basified with a saturated sodium bicarbonate solution. Crudes were washed with ethanol and purified with recrystallization from hot ethanol. Products **24–38** were characterized using ^1^H‐NMR, ^13^C‐NMR, ^19^F‐NMR, high resolution electrospray ionization time‐of‐flight mass spectrometry (HR‐ESITOFMS), and FTIR.

##### 5‐(4‐(Benzyloxy)benzylidene)imidazolidine‐2,4‐Dione (24)

84 mg (57%). m.p 253–254 °C (ref 285–288 °C^[^ ^41^ ^]^). IR (KBr disc) cm^−1^: 3141, 3021, 1756, 1716, 1653. ^1^H‐NMR (500 MHz, DMSO‐*d*_6_) ppm: *δ* 11.16 (s, 1H), 10.42 (s, 1H), 7.58 (d, *J* = 8.4 Hz, 2H), 7.45 (d, *J* = 7.5 Hz, 2H), 7.39 (t, *J* = 7.4 Hz, 2H), 7.33 (t, *J* = 7.1 Hz, 1H), 7.04 (d, *J* = 8.4 Hz, 2H), 6.39 (s, 1H), 5.15 (s, 2H). ^13^C‐NMR (126 MHz, DMSO‐*d*_6_) ppm: *δ* 165.63 (C4), 158.52 (C2), 155.64 (C4a), 136.85 (C1b), 131.10 (C2a, C6a), 128.48 (C3b, C5b), 127.92 (C4b), 127.73 (C2b, C6b), 126.18 (C1a), 125.67 (C5), 115.17 (C3a, C5a), 108.62 (C6), 69.31 (C7). HR‐ESITOFMS (positive mode) *m/z*: [M + H]^+^ 295.1074 (calcd. [M + H]^+^ for C_17_H_15_N_2_O_3_ 295.1077).

##### 5‐(4‐(Benzyloxy)‐3‐Methoxybenzylidene)imidazolidine‐2,4‐Dione (25)

66 mg (41%). m.p. 250‐251 °C (ref. 251–253 °C^[^ ^42^ ^]^). IR (KBr disc) cm^−1^: 3155, 3059, 1757, 1714, 1650. ^1^H‐NMR (500 MHz, DMSO‐*d*_6_) ppm: *δ* 11.17 (s, 1H), 10.49 (s, 1H), 7.45 (d, *J* = 7.4 Hz, 2H), 7.40 (t, *J* = 7.0 Hz, 2H), 7.34 (t, *J* = 7.1 Hz, 1H), 7.18–7.15 (m, 2H), 7.05 (d, *J* = 7.8 Hz, 1H), 6.39 (s, 1H), 5.13 (s, 2H), 3.84 (s, 3H). ^13^C‐NMR (126 MHz, DMSO‐*d*_6_) ppm: *δ* 165.61 (C4), 155.75 (C2), 149.12 (C4a), 148.29 (C3a), 136.91 (C1b), 128.44 (C3b, C5b), 127.91 (C4b), 127.81 (C2b, C6b), 126.25 (C5), 126.01 (C1a), 122.86 (C2a), 113.44 (C5a), 112.90 (C6a), 109.11 (C6), 69.82 (C7), 55.77 (C3aa). HR‐ESITOFMS (negative mode) *m/z*: [*M*‐H]^−^ 323.1034 (calcd. [*M*‐H]^−^ for C_18_H_15_N_2_O_4_ 323.1037).

##### 5‐(4‐(Benzyloxy)‐3‐Ethoxybenzylidene)imidazolidine‐2,4‐Dione (26)

106 mg (62%). m.p. 255–256 °C IR (KBr disc) cm^−1^: 3173, 3057, 1764, 1713, 1654. ^1^H‐NMR (500 MHz, DMSO‐*d*_6_) ppm: *δ* 11.08 (s, 1H), 10.52 (s, 1H), 7.45 (d, *J* = 7.2 Hz, 2H), 7.39 (t, *J* = 7.3 Hz, 2H), 7.32 (t, *J* = 6.9 Hz, 1 H), 7.15 (s, 2 H), 7.03 (d, *J* = 8.8 Hz, 1H), 6.37 (s, 1H), 5.15 (s, 2H), 4.12 (q, *J* = 13.7, 6.8 Hz, 2H), 1.33 (t, *J* = 6.8 Hz, 3H). ^13^C‐NMR (126 MHz, DMSO‐*d*_6_) ppm: *δ* 165.63 (C4), 155.76 (C2), 148.53 (C4a), 148.47 (C3a), 137.09 (C1b), 128.44 (C3b, C5b), 127.82 (C4b), 127.56 (C2b, C6b), 126.24 (C5), 126.13 (C1a), 122.94 (C2a), 114.39 (C5a), 114.01 (C6a), 109.09 (C6), 69.85 (C7), 64.06 (C3aa), 14.76 (C3ab). HR‐ESITOFMS (negative mode) *m/z*: [*M*‐H]^−^ 337.1198 (calcd. [*M*‐H]^−^ for C_19_H_17_N_2_O_4_ 337.1194).

##### 5‐(4‐((2‐Fluoro‐5‐(trifluoromethyl)benzyl)oxy)benzylidene)imidazolidine‐2,4‐Dione (27)

127 mg (66%). m.p. 262–263 °C IR (KBr disc) cm^−1^: 3176, 3064, 1757, 1715, 1653 ^1^H‐NMR (500 MHz, DMSO‐*d*_6_) ppm: *δ* 10.80 (s, 2H), 8.00 (d, *J* = 5.0 Hz, 1H), 7.84 (s, 1H), 7.61 (d, *J* = 8.7 Hz, 2H), 7.52 (t, *J* = 9.1 Hz, 1H), 7.09 (d, *J* = 8.7 Hz, 2H), 6.39 (s, 1H), 5.27 (s, 2H). ^13^C‐NMR (126 MHz, DMSO‐*d*_6_) ppm: *δ* 166.17 (C4), 163.90 (C2b, d, ^1^ *J*_C‐F_ = 253 Hz), 158.49 (C2), 156.24 (C4a), 131.55 (C2a, C6a), 128.51 (C4b, m), 128.39 (C6b, m), 124.23 (C5ba, q, ^1^ *J*_C‐F_ = 270 Hz), 126.93 (C1a), 126.63 (C5), 125.91 (C5b, d, ^2^ *J*_C‐F_ = 31 Hz), 125.72 (C1b, ^2^ *J*_C‐F_ = 16 Hz), 117.26 (C3b, d, ^2^ *J*_C‐F_ = 23 Hz), 115.52 (C3a and C5a), 108.73 (C6), 63.59 (C7). ^19^ F‐NMR (470 MHz, DMSO‐*d*_6_) ppm: *δ* −60.47 (CF_3_), −111.65 (F). HR‐ESITOFMS (negative mode) *m/z*: [*M*‐H]^−^ 379.0712 (calcd. [*M*‐H]^−^ for C_18_H_11_F_4_N_2_O_3_ 379.0711).

##### (Z)‐5‐(4‐((2‐Fluoro‐5‐(trifluoromethyl)benzyl)oxy)‐3‐Methoxybenzylidene)imidazolidine‐2,4‐Dione (28)

137 mg (66%). m.p. 265–267 °C IR (KBr disc) cm^−1^: 3152, 3054, 1769, 1713, 1655. ^1^H‐NMR (500 MHz, DMSO‐*d*_6_) ppm: *δ* 11.16 (s, 1H), 10.55 (s, 1H), 7.99 (d, *J* = 5.9 Hz, 1H), 7.84 (s, 1H), 7.52 (t, *J* = 9.1 Hz, 1H), 7.21 (dd, *J* = 8.4, 1.4 Hz, 1H), 7.17 (s, 1H), 7.13 (d, *J* = 8.4 Hz, 1H), 6.39 (s, 1H), 5.24 (s, 2H), 3.83 (s, 3H). ^13^C‐NMR (126 MHz, DMSO‐*d*_6_) ppm: *δ* 165.60 (C4), 162.42 (C2b, ^1^ *J*_C‐F_ = 253 Hz), 155.78 (C2), 149.18 (C4a), 147.79 (C3a), 127.98 (C4b, m), 127.86 (C6b, m), 126.72 (C5), 126.50 (C1a), 125.46 (C5b, ^2^ *J*_C‐F_ = 26.3 Hz), 125.39 (C1b, ^2^ *J*_C‐F_ = 22.5 Hz), 123.80 (C5ba, ^1^ *J*_C‐F_ = 270 Hz), 122.81 (C5a), 116.79 (C3b, ^2^ *J*_C‐F_ = 22.5 Hz), 113.88 (C6a), 113.00 (C2a), 108.85 (C6), 63.84 (C7), 55.77 (C3aa). ^19^F‐NMR (470 MHz, DMSO‐*d*_6_) ppm: *δ* −60.50 (CF_3_), −111.69 (F). HR‐ESITOFMS (negative mode) *m/z*: [*M*‐H]^−^ 409.0814 (calcd. [*M*‐H]^−^ for C_19_H_13_F_4_N_2_O_4_ 409.0817).

##### (Z)‐5‐(3‐((2‐Fluoro‐5‐(trifluoromethyl)benzyl)oxy)‐4‐Methoxybenzylidene)imidazolidine‐2,4‐Dione (29)

126 mg (61%). m.p. 259–260 °C. IR (KBr disc) cm^−1^: 3421, 3229, 1767, 1716, 1659. ^1^H‐NMR (500 MHz, DMSO‐*d*_6_) ppm: *δ* 11.18 (s, 1H), 10.54 (s, 1H), 8.00 (d, *J* = 6.0 Hz, 1H), 7.84 (s, 1H), 7.53 (t, *J* = 9.0 Hz, 1H), 7.34 (s, 1H), 7.26 (d, *J* = 8.4 Hz, 1H), 7.03 (d, *J* = 8.4 Hz, 1H), 6.39 (s, 1H), 5.31 (s, 2 H), 3.80 (s, 3H). ^13^C‐NMR (126 MHz, DMSO‐*d*_6_) ppm: *δ* 166.03 (C4), 162.79 (C2b, ^1^ *J*_C‐F_ = 250 Hz), 156.21 (C2), 150.15 (C3a), 147.84 (C4a), 127.95 (C4b, m), 127.83 (C6b, m), 126.67 (C1a), 126.15 (C5), 126.09 (C5b, ^2^ *J*_C‐F_ = 16 Hz), 125.89 (C1b, ^2^ *J*_C‐F_ = 32 Hz), 124.27 (C5ba, ^1^ *J*_C‐F_ = 273 Hz), 124.67 (C5a), 117.21 (C3b, ^2^ *J*_C‐F_ = 23 Hz), 114.98 (C6a), 112.69 (C2a), 109.29 (C6) 64.43 (C7), 56.12 (C4aa). ^19^F‐NMR (470 MHz, DMSO‐*d*_6_) ppm: *δ* −60.52 (CF_3_), −111.63 (F). HR‐ESITOFMS (negative mode) *m/z*: [*M*‐H]^−^ 409.0812 (calcd. [*M*‐H]^−^ for C_19_H_13_F_4_N_2_O_4_ 409.0817).

##### (Z)‐5‐(3‐Ethoxy‐4‐((2‐Fluoro‐5‐(trifluoromethyl)benzyl)oxy)benzylidene)imidazolidine‐2,4‐Dione (30)

98 mg (46%). m.p. 258–260 °C. IR (KBr disc) cm^−1^: 3165, 3050, 1768, 1710, 1652. ^1^H‐NMR (500 MHz, DMSO‐*d*_6_) ppm: *δ* 11.14 (s, 1H), 10.52 (s, 1H), 8.01 (d, *J* = 5.6 Hz, 1H), 7.83 (s, 1H), 7.51 (t, *J* = 9.1 Hz, 1H), 7.21–7.18 (m, 2H), 7.14 (d, *J* = 8.3 Hz, 1H), 6.38 (s, 1H), 5.27 (s, 2H), 4.12 (q, *J* = 6.9 Hz, 2H), 1.32 (t, *J* = 6.9 Hz, 3H). ^13^C‐NMR (126 MHz, DMSO‐*d*_6_) ppm: *δ* 165.59 (C4), 162.09 (C2b, ^1^ *J*_C‐F_ = 253 Hz), 155.76 (C2), 148.65 (C4a), 147.97 (C3a), 127.67 (C4b, m), 127.33 (C6b, m), 126.49 (C5), 126.91 (C1a), 125.75 (C5b, ^2^ *J*_C‐F_ = 15 Hz), 125.75 (C1b, ^2^ *J*_C‐F_ = 34 Hz), 123.83 (C5ba, ^1^ *J*_C‐F_ = 270 Hz), 122.92 (C5a), 116.69 (C3b, ^2^ *J*_C‐F_ = 22.5 Hz), 114.63 (C6a), 114.43 (C2a), 108.81 (C6), 64.08 (C7), 63.92 (C3aa), 14.58 (3ab). ^19^ F‐NMR (470 MHz, DMSO‐*d*_6_) ppm: *δ* −60.56 (CF_3_), −111.80 (F). HR‐ESITOFMS (negative mode) *m/z*: [*M*‐H]^−^ 423.0979 (calcd. [*M*‐H]^−^ for C_20_H_15_F_4_N_2_O_4_ 423.0973).

##### (Z)‐5‐(4‐((2,5‐Bis(trifluoromethyl)benzyl)oxy)benzylidene)imidazolidine‐2,4‐Dione (31)

202 mg (94%). m.p. 288–289 °C. IR (KBr disc) cm^−1^: 3223, 3064, 1764, 1716, 1663. ^1^H‐NMR (500 MHz, DMSO‐*d*_6_) ppm: *δ* 11.14 (s, 1H), 10.48 (s, 1H), 8.17 (s, 1H), 8.07 (d, *J* = 8.2 Hz, 1H), 8.00 (d, *J* = 8.2 Hz, 1H), 7.62 (d, *J* = 8.7 Hz, 2H), 7.07 (d, *J* = 8.7 Hz, 2H), 6.39 (s, 1H), 5.37 (s, 2H). ^13^C‐NMR (126 MHz, DMSO‐*d*_6_) ppm: *δ* 166.03 (C4), 158.31 (C2), 156.10 (C4a), 136.99 (C1a), 131.55 (C2a and C6a), 126.91 (C5), 134.64 (C5b, ^2^ *J*_C‐F_  = 18.8 Hz), 132.23 (C2b, ^2^ *J*_C‐F_  = 18.8 Hz), 128.27 (C4b, ^3^ *J*_C‐F_ = 5 Hz), 126.76 (C1b), 126.76 and 126.75 (C6b, ^3^ *J*_C‐F_ = 3.8 Hz), 126.41 (C3b, ^3^ *J*_C‐F_ = 5 Hz), 123.92 (C2ba, ^1^ *J*_C‐F_ = 273 Hz), 123.74 (C5ba, ^1^ *J*_C‐F_ = 271 Hz), 115.52 (C3a and C5a), 108.75 (C6), 66.25 (C7). ^19^ F‐NMR (470 MHz, DMSO‐*d*_6_) ppm: *δ* −59.30 (CF_3_), −61.88 (CF_3_). HR‐ESITOFMS (negative mode) *m/z*: [*M*‐H]^−^ 429.0677 (calcd. [*M*‐H]^−^ for C_19_H_11_F_6_N_2_O_3_ 429.0679).

##### (Z)‐5‐(4‐((2,5‐Bis(trifluoromethyl)benzyl)oxy)‐3‐Methoxybenzylidene)imidazolidine‐2,4‐Dione (32)

132 mg (57%). m.p. 290–291 °C. IR (KBr disc) cm^−1^: 3267, 3150, 1773, 1760, 1654. ^1^H‐NMR (500 MHz, DMSO‐*d*_6_) ppm: *δ* 11.18 (s, 1H), 10.52 (s, 1H), 8.20 (s, 1H), 8.06 (d, *J* = 8.2 Hz, 1H), 7.99 (d, *J* = 8.2 Hz, 1H), 7.20 (d, *J* = 7.6 Hz, 2H), 7.10 (d, *J* = 8.3 Hz, 1H), 6.39 (s, 1H), 5.35 (s, 2H), 3.85 (s, 3H). ^13^C‐NMR (126 MHz, DMSO‐*d*_6_) ppm: *δ* 165.60 (C4), 155.79 (C2), 149.32 (C4a), 147.54 (C3a), 136.94 (C1a), 132.85 (C5b, ^2^ *J*_C‐F_ = 31 Hz), 130.45 (C2b, ^2^ *J*_C‐F_ = 31 Hz), 127.15 (C6b, ^3^ *J*_C‐F_ = 6.3 Hz), 127.05 (C1b), 126.82 (C4b, ^3^ *J*_C‐F_ = 3.8 Hz), 126.65 (C5), 125.77 (C3b, ^3^ *J*_C‐F_ = 3.8 Hz), 124.34 (C5ba, ^1^ *J*_C‐F_ = 273 Hz), 123.33 (C2ba, ^1^ *J*_C‐F_ = 271 Hz), 122.8 (C5a), 114.22 (C6a), 113.08 (C2a), 108.71 (C6), 66.42 (C7), 55.85 (C3aa). ^19^ F‐NMR (470 MHz, DMSO‐*d*_6_) ppm: *δ* −59.26 (CF_3_), −61.94 (CF_3_). HR‐ESITOFMS (negative mode) *m/z*: [*M*‐H]^−^ 459.0780 (calcd. [*M*‐H]^−^ for C_20_H_13_F_6_N_2_O_4_ 459.0785).

##### (Z)‐5‐(3‐((2,5‐Bis(trifluoromethyl)benzyl)oxy)‐4‐Methoxybenzylidene)imidazolidine‐2,4‐Dione (33)

92 mg (40%). m.p. 287–288 °C. IR (KBr disc) cm^−1^: 3176, 3064, 1757, 1715, 1653. ^1^H‐NMR (500 MHz, DMSO‐*d*_6_) ppm: *δ* 11.18 (s, 1H), 10.52 (s, 1H), 8.23 (s, 1H), 8.05 (d, *J* = 8.2 Hz, 1H), 7.98 (d, *J* = 8.6 Hz, 1H), 7.36 (s, 1H), 7.29 (d, *J* = 8.4 Hz, 1H), 7.04 (d, *J* = 8.5 Hz, 1H), 6.39 (s, 1H), 5.40 (s, 2H), 3.81 (s, 3H). ^13^C‐NMR (126 MHz, DMSO‐*d*_6_) ppm: *δ* 165.58 (C4), 155.76 (C2), 149.87 (C4a), 147.21 (C3a), 137.16 (C1a), 132.84 (C5b, ^2^ *J*_C‐F_ = 31 Hz), 130.40 (C2b, ^2^ *J*_C‐F_ = 33 Hz), 127.08 (C6b, ^3^ *J*_C‐F_ = 5 Hz), 126.83 (C4b, ^3^ *J*_C‐F_ = 3.8 Hz), 126.33 (C5a), 125.76 (C5), 125.68 (C3b, ^3^ *J*_C‐F_ = 3.8 Hz), 124.41 (C1b), 115.34 (C6a), 112.37 (C2a), 108.73 (C6), 66.72 (C7), 55.75 (C4aa). ^19^F‐NMR (470 MHz, DMSO‐*d*_6_) ppm: *δ* −59.22 (CF_3_), −61.98 (CF_3_). HR‐ESITOFMS (negative mode) *m/z*: [*M*‐H]^−^ 459.0787 (calcd. [*M*‐H]^−^ for C_20_H_13_F_6_N_2_O_4_ 459.0785).

##### (Z)‐5‐(4‐((2,5‐Bis(trifluoromethyl)benzyl)oxy)‐3‐Ethoxybenzylidene)imidazolidine‐2,4‐Dione (34)

100 mg (42%). m.p. 290–292 °C. IR (KBr disc) cm^−1^: 3207, 3051, 1770, 1713, 1654. ^1^H‐NMR (500 MHz, DMSO‐*d*_6_) ppm: *δ* 11.18 (s, 1H), 10.51 (s, 1H), 8.25 (s, 1H), 8.04 (d, *J* = 8.1 Hz, 1H), 7.96 (d, *J* = 8.2 Hz, 1H), 7.19 (d, *J* = 6.2 Hz, 2H), 7.10 (d, *J* = 8.5 Hz, 1H), 6.38 (s, 1 H), 5.37 (s, 2 H), 4.14 (q, *J* = 6.8 Hz, 2H), 1.33 (d, *J* = 13.5 Hz, 3H^13^C‐NMR (126 MHz, DMSO‐*d*_6_) ppm: *δ* 165.58 (C4), 155.76 (C2), 148.80 (C4a), 147.66 (C3a), 137.33 (C1a), 132.81 (C5b, ^2^ *J*_C‐F_ = 33 Hz), 129.98 (C2b, ^2^ *J*_C‐F_ = 33 Hz), 127.53 (C6b, ^3^ *J*_C‐F_ = 6.3 Hz), 127.22 (C1b), 125.99 (C4b, ^3^ *J*_C‐F_ = 3.8 Hz), 126.61 (C5), 124.98 (C3b, ^3^ *J*_C‐F_ = 3.8 Hz), 124.47 (C5ba, ^1^ *J*_C‐F_ = 274 Hz), 123.39 (C2ba, ^1^ *J*_C‐F_ = 271 Hz), 122.91(C5a), 114.84 (C6a), 114.27 (C2a), 108.73 (C6), 66.32 (C7), 64.05 (C3aa), 14.50 (C3ab). ^19^F‐NMR (470 MHz, DMSO‐*d*_6_) ppm: *δ* −59.54 (CF_3_), −62.05 (CF_3_). HR‐ESITOFMS (negative mode) *m/z*: [*M*‐H]^−^ 473.0946 (calcd. [*M*‐H]^−^ for C_21_H_15_F_6_N_2_O_4_ 473.0941).

##### (Z)‐5‐(2‐Chloro‐5‐(trifluoromethyl)benzylidene)imidazolidine‐2,4‐Dione (35)

68 mg (47%). m.p. 266–267 °C IR (KBr disc) cm^−1^: 3193, 1781, 1749, 1673. ^1^H‐NMR (500 MHz, DMSO‐*d*_6_) ppm: *δ* 11.21 (s, 2H), 7.89 (s, 1H), 7.73 (d, *J* = 8.4 Hz, 1H), 7.66 (d, *J* = 8.2 Hz, 1H), 6.47 (s, 1H). ^13^C‐NMR (126 MHz, DMSO‐*d*_6_) ppm: *δ* 165.11 (C4), 155.88 (C2), 137.02 (C2a), 132.15 (C3a), 131.63 (C1a), 130.59 (C5), 128.33 (C5a, q, ^2^ *J*_C‐F_  = 33 Hz), 126.72 (C4a, d, ^3^ *J*_C‐F_ = 3.5 Hz), 126.22 (C6a, d, ^3^ *J*_C‐F_ = 3.5 Hz), 123.66 (C5aa, q, ^1^ *J*_C‐F_ = 271 Hz), 101.38 (C6). ^19^ F‐NMR (470 MHz, DMSO‐*d*_6_) ppm: *δ* −61.25 (CF_3_). HR‐ESITOFMS (negative mode) *m/z*: [*M*‐H]^−^ 289.0000 (calcd. [*M*‐H]^−^ for C_11_H_5_ClF_3_N_2_O_2_ 288.9997).

##### (Z)‐5‐(2‐Bromo‐4‐Hydroxy‐5‐Methoxybenzylidene)imidazolidine‐2,4‐Dione (36)

106 mg (68%). m.p. 295–296 °C. IR (KBr disc) cm^−1^: 3628, 3420, 1735, 1684, 1570.^1^H‐NMR (500 MHz, DMSO‐*d*_6_) ppm: *δ* 11.24 (s, 1H), 10.60 (s, 1H), 9.91 (s, 1H), 7.06 (s, 2H), 6.44 (s, 1H), 3.87 (s, 3H). ^13^C‐NMR (126 MHz, DMSO‐*d*_6_) ppm: *δ* 165.34 (C4), 155.73 (C2), 148.19 (C5a), 147.33 (C4a), 127.97 (C2a), 123.05 (C1a), 119.13 (C5), 115.17 (C6a), 112.98 (C3a), 106.95 (C6), 55.79 (C5aa). HR‐ESITOFMS (negative mode) *m/z*: [*M*‐H]^−^ 310.9673 (calcd. [*M*‐H]^−^ for C_11_H_8_BrN_2_O_4_ 310.9673).

##### (Z)‐2‐(4‐((2,5‐Dioxoimidazolidin‐4‐Ylidene)methyl)‐2‐Methoxyphenoxy)acetic Acid (37)

74 mg (50%). m.p. 298–299 °C. IR (KBr disc) cm^−1^: 3502, 3144, 1733, 1645. ^1^H‐NMR (500 MHz, DMSO‐*d*_6_) ppm: *δ* 11.17 (s, 1H), 10.50 (s, 1H), 7.15 (s, 2H), 6.86 (d, *J* = 8.3 Hz, 1H), 6.38 (s, 1H), 4.71 (s, 2H), 3.85 (s, 3H). ^13^C‐NMR (126 MHz, DMSO‐*d*_6_) ppm: *δ* 170.03 (C8), 165.61 (C4), 155.76 (C2), 148.86 (C4a), 147.67 (C3a), 126.38 (C1a), 126.33 (C5), 122.70 (C2a), 113.02 (C5a), 112.35 (C6a), 108.98 (C6), 64.87 (C7), 55.77 (C3aa). HR‐ESITOFMS (negative mode) *m/z*: [*M*‐H]^−^ 291.0618 (calcd. [*M*‐H]^−^ for C_13_H_11_N_2_O_6_ 291.0623).

##### (E)‐Ethyl 5‐((2,5‐Dioxoimidazolidin‐4‐Ylidene)methyl)‐2,4‐Dimethyl‐1H‐Pyrrole‐3‐Carboxylate (38)

37 mg (27%). m.p. 301–302 °C. IR (KBr disc) cm^−1^: 3385, 3153, 1724, 1626. ^1^H‐NMR (500 MHz, DMSO‐*d*_6_) ppm: *δ* 12.24 (s, 1H), 11.38 (s, 1H), 10.09 (s, 1H), 6.31 (s, 1H), 4.17 (q, 2H), 2.45 (s, 3H), 2.23 (s, 3H), 1.27 (t, 3H). ^13^C‐NMR (126 MHz, DMSO‐*d*_6_) ppm: *δ* 165.78 (C3b), 164.54 (C4), 152.95 (C2), 137.10 (C1a), 125.31 (C5), 122.93 (C3a), 112.06 (C4a), 104.95 (C2a), 97.39 (C6), 58.89 (C3c), 14.30 (C4b), 14.16 (C2b), 10.96 (C3d). HR‐ESITOFMS (negative mode) *m/z*: [*M*‐H]^−^ 276.0993 (calcd. [*M*‐H]^−^ for C_13_H_14_N_3_O_4_ 276.0990).

##### Enzymatic Kinase Assay

The method for the assay was done as previously described^[^ ^28^, ^29^ ^]^ and modified by Elvira and coworkers.^[^ ^43^ ^]^ In summary, the tested compound was prepared at 5% in DMSO and diluted using 4× kinase buffer (64.5 μL) and nuclease‐free water (175 μL) to reach a final concentration of 10 μM. Additionally, each kinase stock was diluted with 2.5× reaction buffer (95 μL), and an 80 μM ATP solution (20 μL) was used to dilute the substrate/cofactor stock. The assay involved dispensing 1 μL of the tested compound, 2 μL of ATP/substrate, and 2 μL of kinase into each well of 384‐well plates. The plates were then left to respond for an hour at a temperature between 22 and 25 °C. Next, 5 μL of ADP‐Glo reagent was added, and the mixture was incubated for 40 min at 22–25 °C. After that, 10 μL of kinase detection reagent was added and incubated for 30 min. Following the process, the kinase activity was determined by measuring the luminescence. The well without the tested drug solution was the negative control (100% activity), and the well without the enzyme solution produced background luminescence (0% activity). The percentage of kinase activity was determined by deducing the background light from each and every kinase reaction. This assay utilized Erlotinib (1 μM) as the positive control.

##### Molecular Docking

Target protein (PDB ID: 3PP0) was retrieved from the protein data bank website (https://www.rcsb.org/). Water molecules, ions, and other nonprotein molecules were removed. Hydrogen atoms were added, and Gasteiger charges were calculated using AutoDock Tools (ADT). The target protein structure was saved as a pdbqt file. Hydrogens were added to SYR127063 as reference ligands and saved as a pdbqt file. The grid box dimension was defined as 20 × 20 × 20, with the center of the grid box as 16.197 × 17.394 × 26.218. Molecular docking was performed using AutoDock Vina with an exhaustiveness value of 24^[^ ^44^ ^]^ in Windows Subsystem for Linux (WSL2) with Processor Intel(R) Core(TM) i5‐6300U CPU @ 2.40 GHz, 2496 MHz, 2 Core(s), 4 Logical Processor(s). Tested compounds were drawn using ChemDraw to generate smiles files. The 2D Structure was minimized using gen3d OpenBabel^[^ ^45^ ^]^ and further converted to pdbqt using the same tool. The molecular docking result was visualized using ChimeraX^[^ ^46^ ^]^ and Discovery Studio Visualizer. The same procedure was also applied in vinardo and smina molecular docking. For AutoDock4, ligand parameterization is carried out using Meeko (https://meeko.readthedocs.io/en/release‐doc/). Homology modeling is performed using MODELLER 10.5 with the following procedure described in https://salilab.org/modeller/documentation.html.^[^ ^47^ ^]^

## Conflict of Interest

The authors declare no conflict of interest.

## Supporting information

Supplementary Material

## Data Availability

The data that support the findings of this study are available in the supplementary material of this article.
