# Supplementary material for: Design, Synthesis, Bioevaluation, and Bioinformatics Study of 5‐Benzylidene Hydantoin Derivatives as Novel Tyrosine Kinase Inhibitors
Source: ChemistryOpen. 2025 Oct 9;15(1):e202500158. doi: 10.1002/open.202500158 (PMC12835546; doi:10.1002/open.202500158)
Supplement: Supplementary file 1 — Supplementary Material [file OPEN-15-e202500158-s001.pdf]

Design, Synthesis, Bioevaluation, and Bioinformatics Study of 5-Benzylidene Hydantoin Derivatives as Novel Tyrosine Kinase Inhibitors

Muhammad Naufal,[a] Elvira Hermawati,[b] Ade Danova,[b] Ika Wiani Hidayat[a] and Jamaludin Al-Anshori*[a]

[a]*Department of Chemistry, Faculty of Mathematics and Natural Sciences, Universitas Padjadjaran, Jl. Raya Bandung-Sumedang km.21, Jatinangor, 45363, Indonesia.*
[b]*Division of Organic Chemistry, Faculty of Mathematics and Natural Sciences, Institut Teknologi Bandung, Jl. Ganesha 10, Bandung 40132, Indonesia.*

Figure SI 1. ^{1}H-NMR 500 MHz of 9 in acetone-d_6. 3
Figure SI 2. ^{13}CNMR 125 MHz of 9 in acetone-d_6. 3
Figure SI 3 ^{1}H-NMR 500 MHz of 10 in acetone-d_6. 4
Figure SI 4 ^{13}CNMR 125 MHz of 10 in acetone-d_6. 4
Figure SI 5 ^{1}H-NMR 500 MHz of 11 in acetone-d_6. 5
Figure SI 6 ^{13}CNMR 125 MHz of 11 in acetone-d_6. 5
Figure SI 7 ^{1}H-NMR 500 MHz of 12 in acetone-d_6. 6
Figure SI 8 ^{13}CNMR 125 MHz of 12 in acetone-d_6. 6
Figure SI 9 ^{1}H-NMR 500 MHz of 13 in dimethyl sulfoxide-d_6. 7
Figure SI 10 ^{13}CNMR 125 MHz of 13 in dimethyl sulfoxide-d_6. 7
Figure SI 11 ^{1}H-NMR 500 MHz of 14 in dimethyl sulfoxide-d_6. 8
Figure SI 12 ^{13}CNMR 125 MHz of 14 in dimethyl sulfoxide-d_6. 8
Figure SI 13 ^{1}H-NMR 500 MHz of 15 in acetone-d_6 9
Figure SI 14 ^{13}CNMR 125 MHz of 15 in acetone-d_6. 9
Figure SI 15 ^{1}H-NMR 500 MHz of 16 in acetone-d_6. 10
Figure SI 16 ^{13}CNMR 125 MHz of 16 in acetone-d_6. 10
Figure SI 17 ^{1}H-NMR 500 MHz of 17 in acetone-d_6. 11
Figure SI 18 ^{13}CNMR 125 MHz of 17 in acetone-d_6. 11
Figure SI 19 ^{1}H-NMR 500 MHz of 18 in acetone-d_6. 12
Figure SI 20 ^{13}CNMR 125 MHz of 18 in acetone-d_6. 12
Figure SI 21 ^{1}H-NMR 500 MHz of 19 in acetone-d_6. 13
Figure SI 22 ^{13}CNMR 125 MHz of 19 in acetone-d_6. 13
Figure SI 23 ^{1}H-NMR 500 MHz of 20 in acetone-d_6. 14
Figure SI 24 ^{13}CNMR 125 MHz of 20 in acetone-d_6. 14
Figure SI 25 Infrared spectra of 24 in KBr pellets. 15
Figure SI 26 HR-TOF-MS-ES$^+$ spectra of 24. 15
Figure SI 27 ^{1}H-NMR 500 MHz of 24 in dimethyl sulfoxide-d_6. 16
Figure SI 28 ^{13}CNMR 125 MHz of 24 in dimethyl sulfoxide-d_6. 16
Figure SI 29 Infrared spectra of 25 in KBr pellets. 17
Figure SI 30 HR-TOF-MS-ES$^-$ spectra of 25. 17
Figure SI 31 ^{1}H-NMR 500 MHz of 25 in dimethyl sulfoxide-d_6. 18
Figure SI 32 ^{13}CNMR 125 MHz of 25 in dimethyl sulfoxide-d_6. 18
Figure SI 33 Infrared spectra of 26 in KBr pellets. 19
Figure SI 34 HR-TOF-MS-ES$^-$ spectra of 26. 19
Figure SI 35 ^{1}H-NMR 500 MHz of 26 in dimethyl sulfoxide-d_6. 20
Figure SI 36 ^{13}CNMR 125 MHz of 26 in dimethyl sulfoxide-d_6. 20
Figure SI 37 Infrared spectra of 27 in KBr pellets. 21
Figure SI 38 HR-TOF-MS-ES$^-$ spectra of 27. 21
Figure SI 39 ^{1}H-NMR 500 MHz of 27 in dimethyl sulfoxide-d_6. 22
Figure SI 40 ^{13}CNMR 125 MHz of 27 in dimethyl sulfoxide-d_6. 22
Figure SI 41 Infrared spectra of 28 in KBr pellets. 23
Figure SI 42 HR-TOF-MS-ES- spectra of 28. 23
Figure SI 43 ^{1}H-NMR 500 MHz of 28 in dimethyl sulfoxide-d_6. 24
Figure SI 44 ^{13}CNMR 125 MHz of 28 in dimethyl sulfoxide-d_6. 24
Figure SI 45 Infrared spectra of 29 in KBr pellets. 25
Figure SI 46 HR-TOF-MS-ES- spectra of 29. 25
Figure SI 47 ^{1}H-NMR 500 MHz of 29 in dimethyl sulfoxide-d_6. 26
Figure SI 48 ^{13}CNMR 125 MHz of 29 in dimethyl sulfoxide-d_6. 26
Figure SI 49 Infrared spectra of 24 in KBr pellets. 27
Figure SI 50 HR-TOF-MS-ES- spectra of 30. 27

Figure SI 51 ^{1}H-NMR 500 MHz of 30 in dimethyl sulfoxide-d_6. ... 28
Figure SI 52 ^{13}CNMR 125 MHz of 30 in dimethyl sulfoxide-d_6. ... 28
Figure SI 53 Infrared spectra of 31 in KBr pellets. ... 29
Figure SI 54 HR-TOF-MS-ES- spectra of 31. ... 29
Figure SI 55 ^{1}H-NMR 500 MHz of 31 in dimethyl sulfoxide-d_6. ... 30
Figure SI 56 ^{13}CNMR 125 MHz of 31 in dimethyl sulfoxide-d_6. ... 30
Figure SI 57 Infrared spectra of 32 in KBr pellets. ... 31
Figure SI 58 HR-TOF-MS-ES- spectra of 32. ... 31
Figure SI 59 ^{1}H-NMR 500 MHz of 32 in dimethyl sulfoxide-d_6. ... 32
Figure SI 60 ^{13}CNMR 125 MHz of 32 in dimethyl sulfoxide-d_6. ... 32
Figure SI 61 Infrared spectra of 33 in KBr pellets. ... 33
Figure SI 62 HR-TOF-MS-ES- spectra of 33. ... 33
Figure SI 63 ^{1}H-NMR 500 MHz of 33 in dimethyl sulfoxide-d_6. ... 34
Figure SI 64 ^{13}CNMR 125 MHz of 33 in dimethyl sulfoxide-d_6. ... 34
Figure SI 65 Infrared spectra of 34 in KBr pellets. ... 35
Figure SI 66 HR-TOF-MS-ES- spectra of 34. ... 35
Figure SI 67 ^{1}H-NMR 500 MHz of 34 in dimethyl sulfoxide-d_6. ... 36
Figure SI 68 ^{13}CNMR 125 MHz of 34 in dimethyl sulfoxide-d_6. ... 36
Figure SI 69 Infrared spectra of 35 in KBr pellets. ... 37
Figure SI 70 HR-TOF-MS-ES- spectra of 35. ... 37
Figure SI 71. ^{1}H-NMR 500 MHz of 35 in dimethyl sulfoxide-d_6. ... 38
Figure SI 72 ^{13}CNMR 125 MHz of 35 in dimethyl sulfoxide-d_6. ... 38
Figure SI 73 Infrared spectra of 36 in KBr pellets. ... 39
Figure SI 74 HR-TOF-MS-ES- spectra of 36. ... 39
Figure SI 75 ^{1}H-NMR 500 MHz of 36 in dimethyl sulfoxide-d_6. ... 40
Figure SI 76 ^{13}CNMR 125 MHz of 36 in dimethyl sulfoxide-d_6. ... 40
Figure SI 77. Infrared spectra of 37 in KBr pellets. ... 41
Figure SI 78 HR-TOF-MS-ES- spectra of 37. ... 41
Figure SI 79 ^{1}H-NMR 500 MHz of 37 in dimethyl sulfoxide-d_6. ... 42
Figure SI 80 ^{13}CNMR 125 MHz of 37 in dimethyl sulfoxide-d_6. ... 42
Figure SI 81 Infrared spectra of 38 in KBr pellets. ... 43
Figure SI 82 Infrared spectra of 38 in KBr pellets. ... 43
Figure SI 83 ^{1}H-NMR 500 MHz of 38 in dimethyl sulfoxide-d_6. ... 44
Figure SI 84 ^{13}CNMR 125 MHz of 38 in dimethyl sulfoxide-d_6. ... 44
Figure SI 85 ^{19}FNMR spectra of 27-30. ... 45
Figure SI 86 ^{19}FNMR spectra of 31-34. ... 45

4-(benzyloxy)benzaldehyde (**9**)

^{1}H NMR (500 MHz, acetone)δ 9.91 (s, 1H), 7.89 (d, *J* = 8.8 Hz, 2H), 7.51 (d, *J* = 7.4 Hz, 2H), 7.41 (t, *J* = 7.4 Hz, 2H), 7.35 (t, *J* = 7.3 Hz, 1H), 7.21 (d, *J* = 8.7 Hz, 2H), 5.26 (s, 2H).

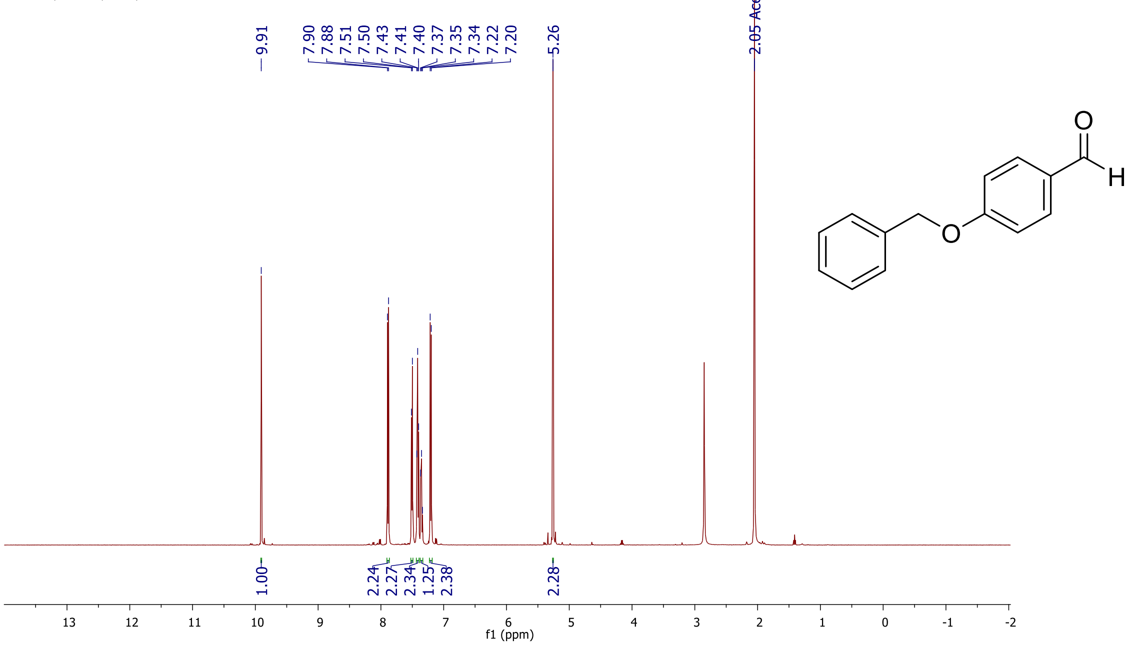

Figure SI 1. ^{1}H-NMR 500 MHz of 9 in acetone-d_6.

^{13}C NMR (126 MHz, acetone)δ 191.17, 164.62, 137.56, 132.50, 131.34, 129.39, 128.91, 128.58, 116.08, 70.86, 29.84.

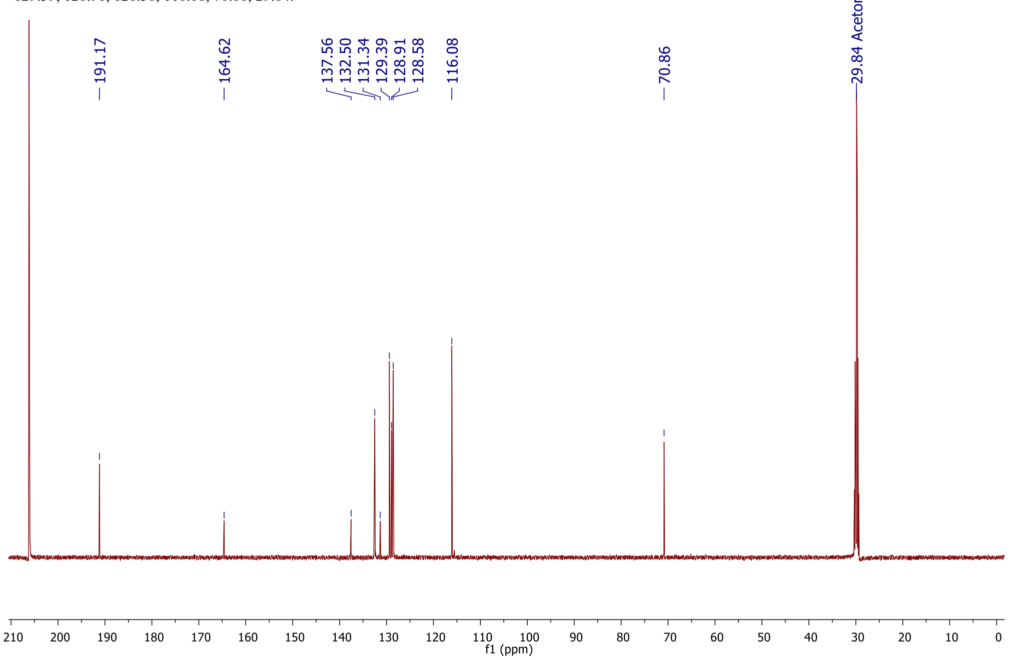

Figure SI 2. ^{13}CNMR 125 MHz of 9 in acetone-d_6.

4-(benzyloxy)-3-methoxybenzaldehyde (**10**).

^{1}H NMR (500 MHz, acetone)δ 9.85 (s, 1H), 7.50 (d, *J* = 7.1 Hz, 3H), 7.44 (d, *J* = 1.4 Hz, 1H), 7.39 (t, *J* = 7.4 Hz, 2H), 7.33 (t, *J* = 7.3 Hz, 1H), 7.22 (d, *J* = 8.2 Hz, 1H), 5.23 (s, 2H), 3.88 (s, 3H).

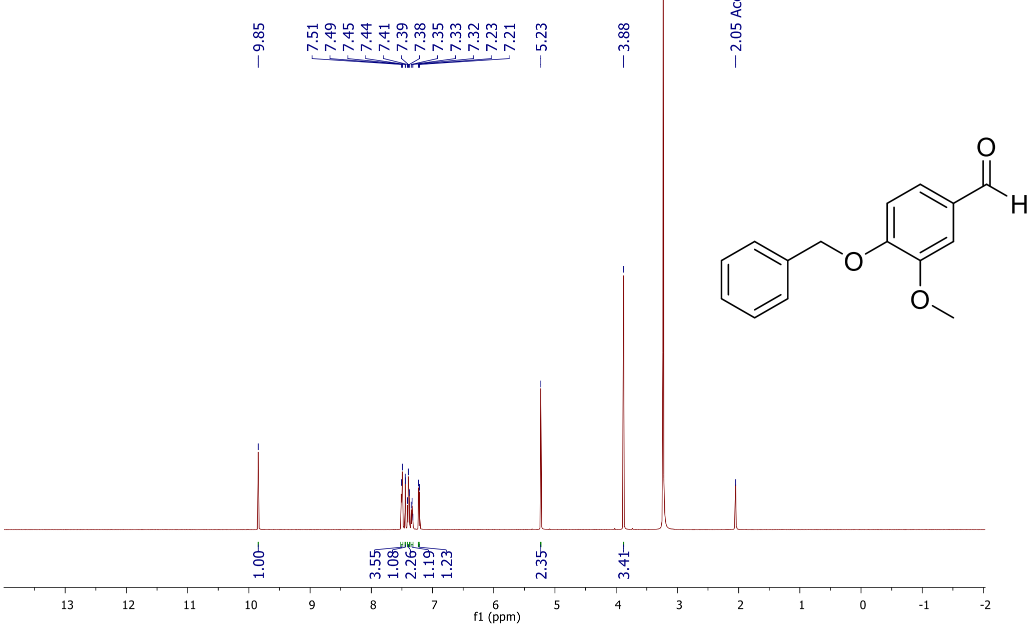

Figure SI 3 ^{1}H-NMR 500 MHz of 10 in acetone-d_6.

^{13}C NMR (126 MHz, acetone)δ 191.56, 154.55, 150.95, 137.51, 131.29, 129.29, 128.83, 128.53, 126.64, 113.48, 110.60, 71.19, 56.14, 29.84.

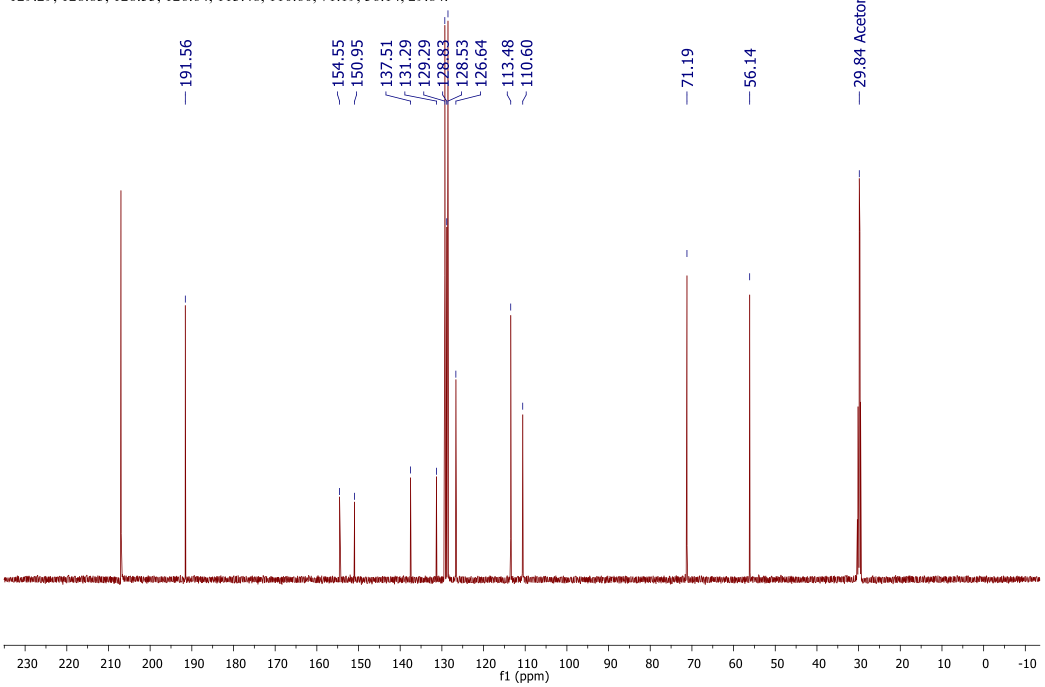

Figure SI 4 ^{13}CNMR 125 MHz of 10 in acetone-d_6.

4-(benzyloxy)-3-ethoxybenzaldehyde (**11)**

^{1}H NMR (500 MHz, acetone)δ 9.85 (s, 1H), 7.50 (dd,*J* = 12.4, 4.5 Hz, 3H), 7.44 (d, *J* = 1.6 Hz, 1H), 7.41 (t,*J* = 7.5 Hz, 2H), 7.34 (t,*J* = 7.3 Hz, 1H), 7.23 (d, *J* = 8.2 Hz, 1H), 5.27 (s, 2H), 4.16 (q,*J* = 7.0 Hz, 2H), 1.41 (t,*J* = 7.0 Hz, 3H).

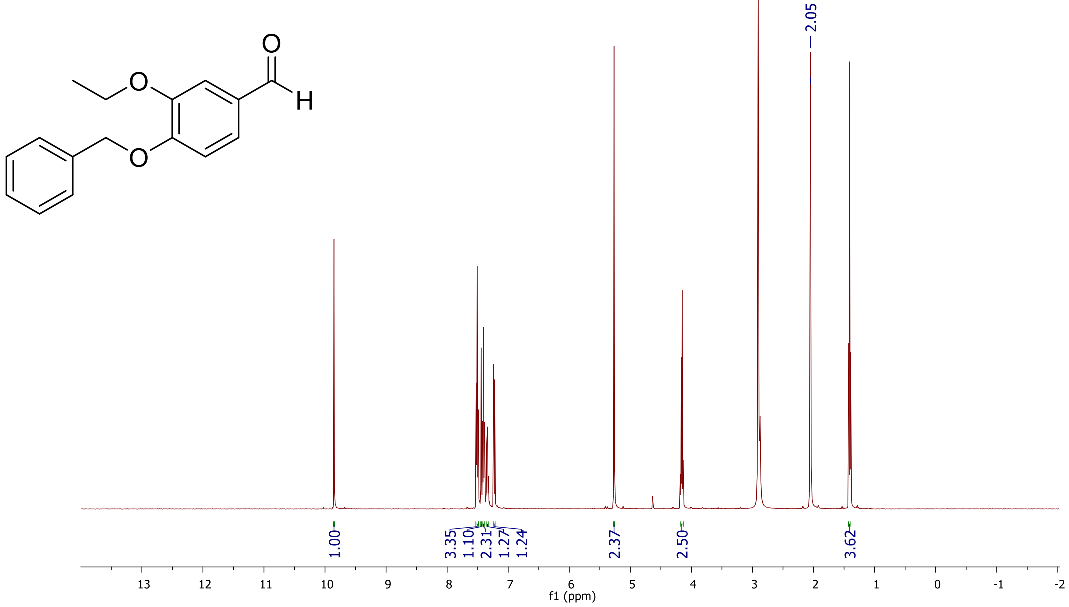

Figure SI 5 ^{1}H-NMR 500 MHz of 11 in acetone-d_6.

^{13}C NMR (126 MHz, acetone)δ 191.35, 154.83, 150.37, 137.80, 131.51, 129.33, 128.79, 128.36, 126.42, 114.01, 112.17, 71.25, 65.13, 29.84, 15.05.

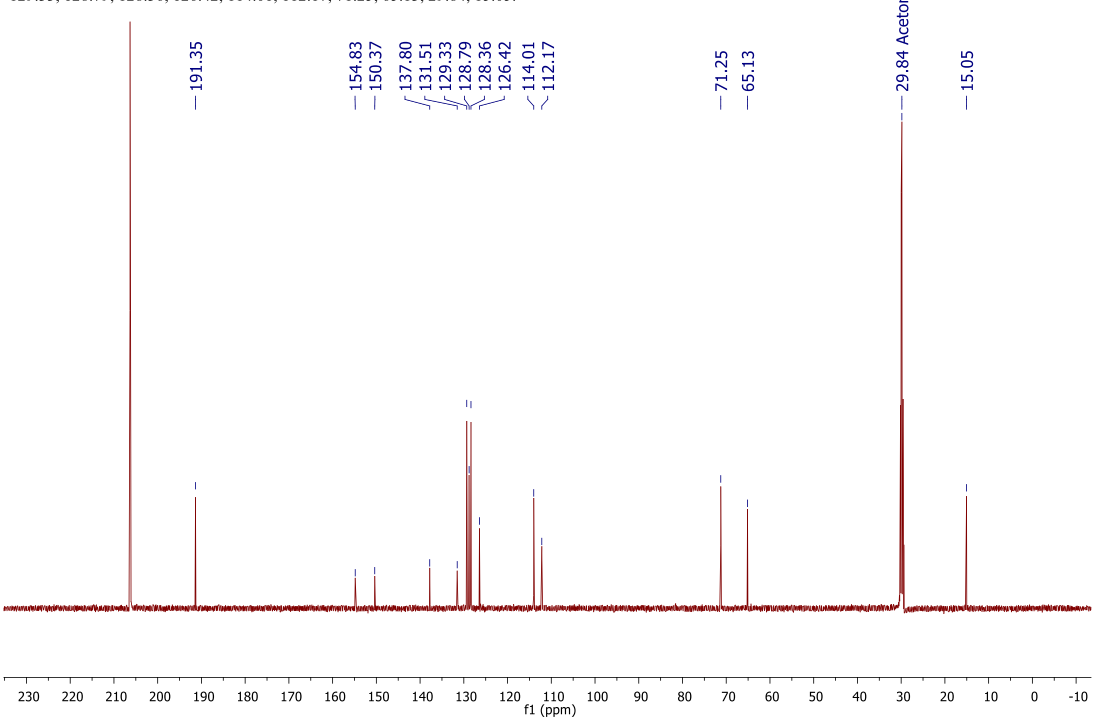

Figure SI 6 ^{13}CNMR 125 MHz of 11 in acetone-d_6.

4-((2-fluoro-5-(trifluoromethyl)benzyl)oxy)benzaldehyde (**12**)

^{1}H NMR (500 MHz, acetone) δ 9.93 (s, 1H), 8.01 (d, *J* = 6.2 Hz, 1H), 7.93 (d, *J* = 8.9 Hz, 2H), 7.84 (s, 1H), 7.48 (t, *J* = 9.1 Hz, 1H), 7.28 (d, *J* = 8.7 Hz, 2H), 5.41 (s, 2H).

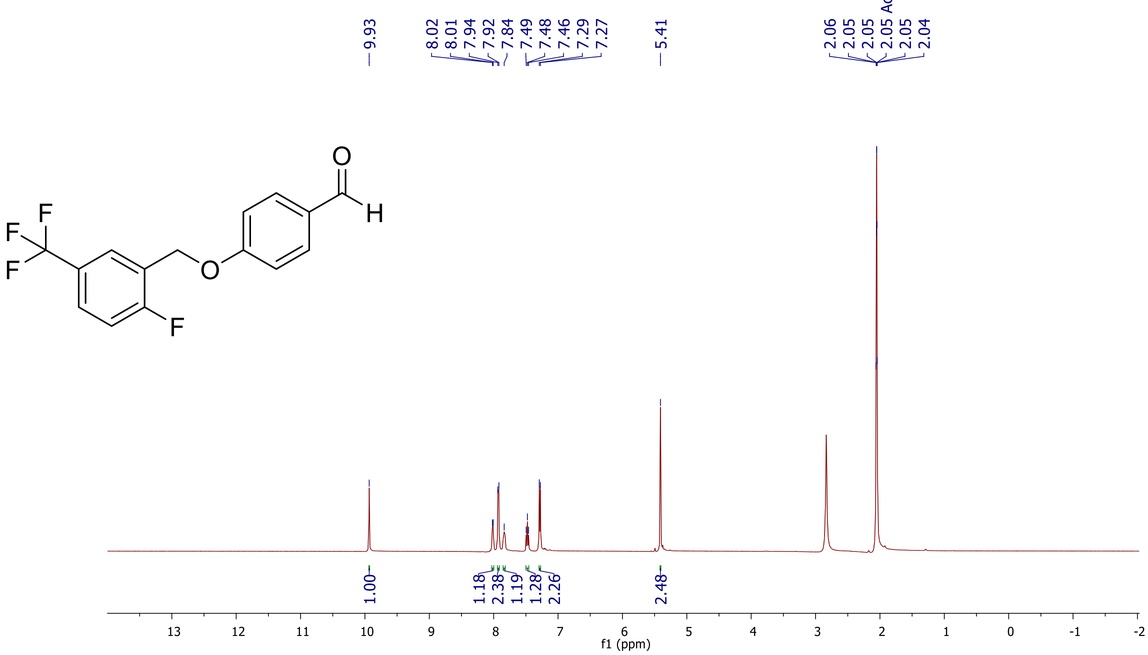

Figure SI 7 ^{1}H-NMR 500 MHz of 12 in acetone-d_6.

^{13}C NMR (126 MHz, acetone) δ 191.24, 164.61, 164.07, 162.60, 132.58, 131.78, 128.79, 128.58, 126.19, 117.55, 117.37, 116.03, 64.35

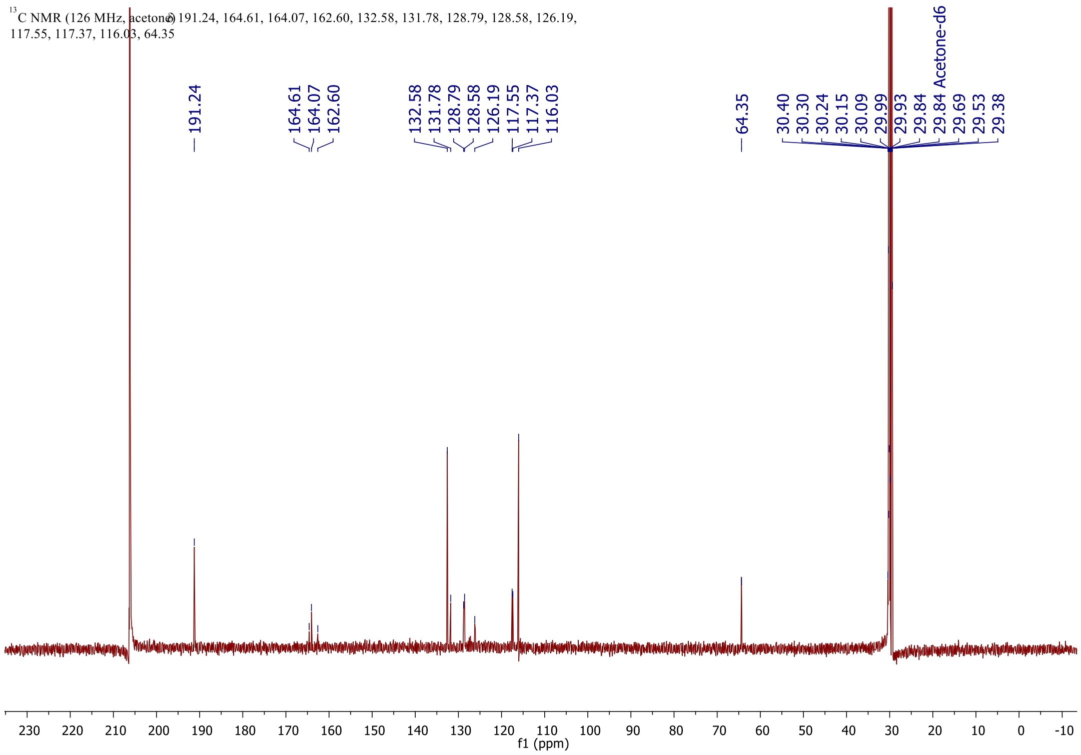

Figure SI 8 ^{13}CNMR 125 MHz of 12 in acetone-d_6.

4-((2-fluoro-5-(trifluoromethyl)benzyl)oxy)-3-methoxybenzaldehyde (**13)**

^{1}H NMR (500 MHz, dmso) δ 9.87 (s, 1H), 8.02 (d, J = 6.1 Hz, 1H), 7.86 (s, 1H), 7.58 (d, J = 8.1 Hz, 1H), 7.53 (t, J = 9.1 Hz, 1H), 7.44 (s, 1H), 7.36 (d, J = 8.2 Hz, 1H), 5.32 (s, 2H), 3.83 (s, 3H).

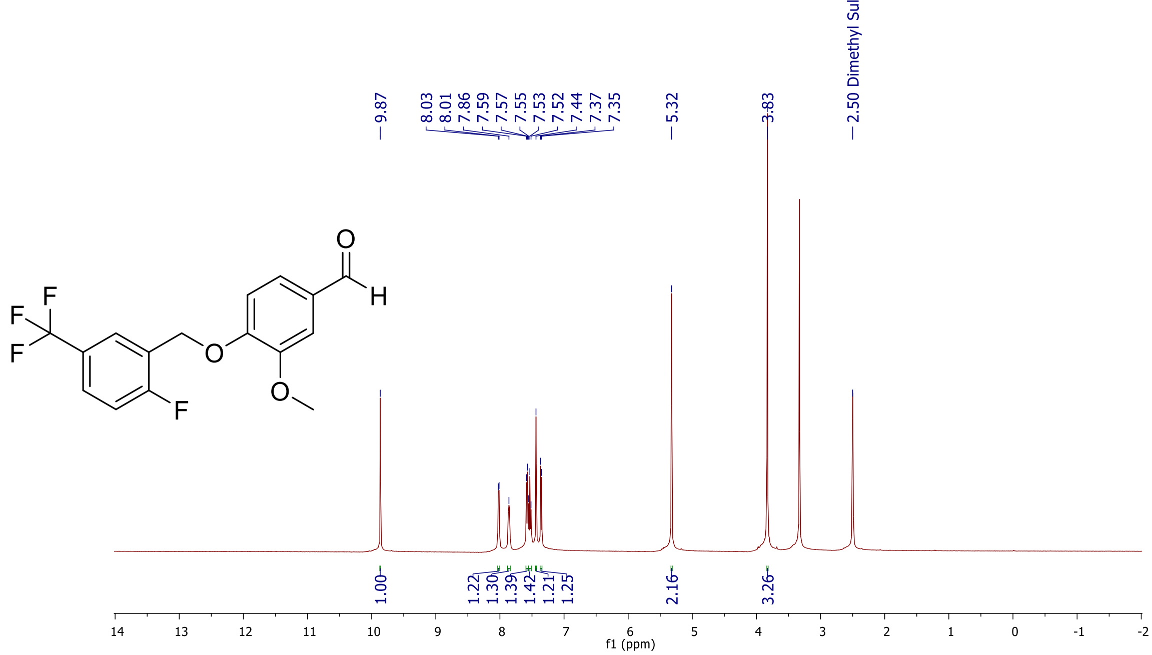

Figure SI 9 ^{1}H-NMR 500 MHz of 13 in dimethyl sulfoxide-d_6.

^{13}C NMR (126 MHz, dmso) δ 191.42, 163.51, 161.50, 152.66, 149.39, 130.29, 128.15, 125.72, 125.61, 125.35, 124.91, 124.78, 122.66, 116.94, 116.76, 112.86, 109.96, 63.91, 55.61, 39.52, 39.52.

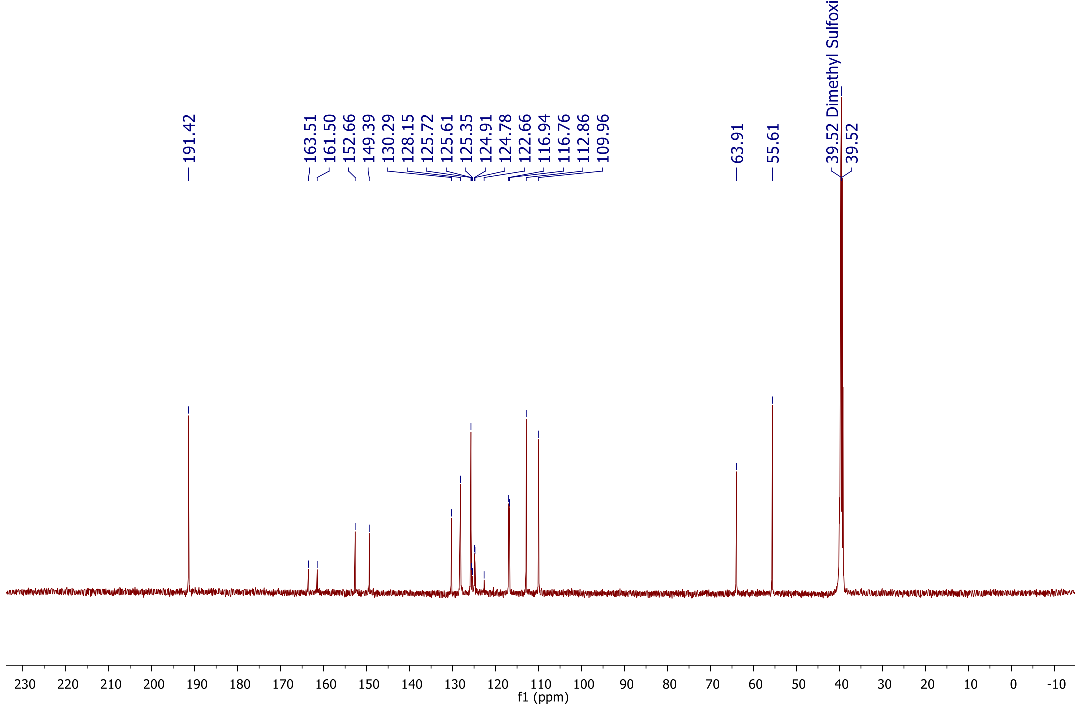

Figure SI 10 ^{13}CNMR 125 MHz of 13 in dimethyl sulfoxide-d_6.

3-((2-fluoro-5-(trifluoromethyl)benzyl)oxy)-4-methoxybenzaldehyde (**14**)

^{1}H NMR (500 MHz, dmso)δ 9.85 (s, 1H), 8.00 (d, *J* = 6.1 Hz, 1H), 7.84 (s, 1H), 7.61 (d, *J* = 8.3 Hz, 1H), 7.57 (s, 1H), 7.53 (d, *J* = 9.2 Hz, 1H), 7.22 (d, *J* = 8.3 Hz, 1H), 5.27 (s, 2H), 3.87 (s, 3H).

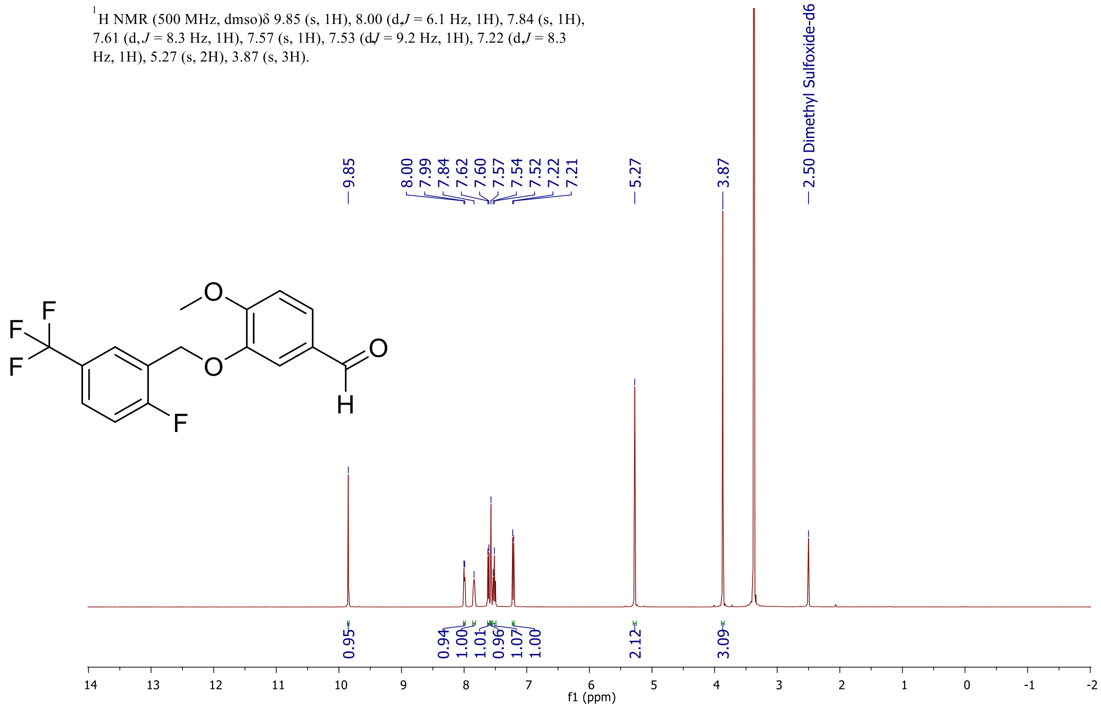

Figure SI 11 ^{1}H-NMR 500 MHz of 14 in dimethyl sulfoxide-d_6.

^{13}C NMR (126 MHz, dmso)δ 191.32, 163.50, 161.49, 154.55, 147.77, 129.63, 128.11, 126.80, 125.62, 125.27, 125.14, 124.88, 122.72, 116.92, 116.74, 111.79, 63.92, 56.04.

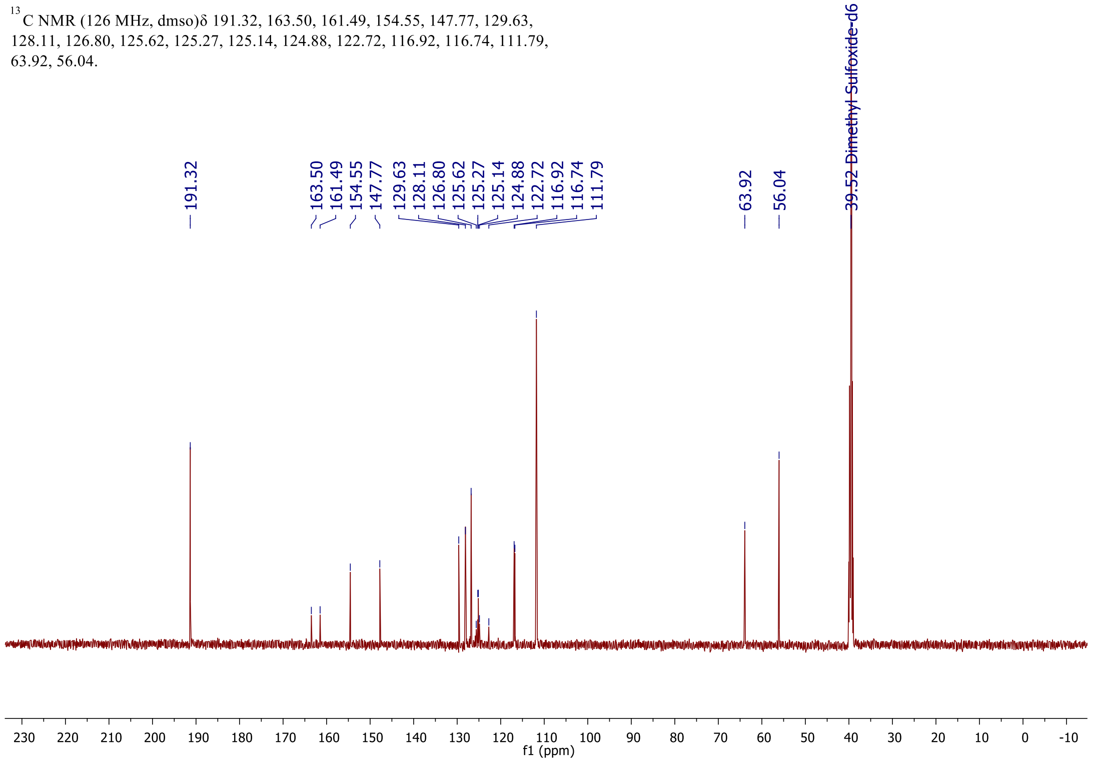

Figure SI 12 ^{13}CNMR 125 MHz of 14 in dimethyl sulfoxide-d_6.

3-ethoxy-4-((2-fluoro-5-(trifluoromethyl)benzyl)oxy)benzaldehyde **(15)**

^{1}H NMR (500 MHz, acetone) δ 9.88 (s, 1H), 8.07 (d, J = 6.2 Hz, 1H), 7.81 (s, 1H), 7.56 (d, J = 8.2 Hz, 1H), 7.48 (s, 1H), 7.44 (d, J = 9.1 Hz, 1H), 7.34 (d, J = 8.2 Hz, 1H), 5.40 (s, 2H), 4.17 (q, J = 6.9 Hz, 2H), 1.42 (t, J = 6.9 Hz, 3H).

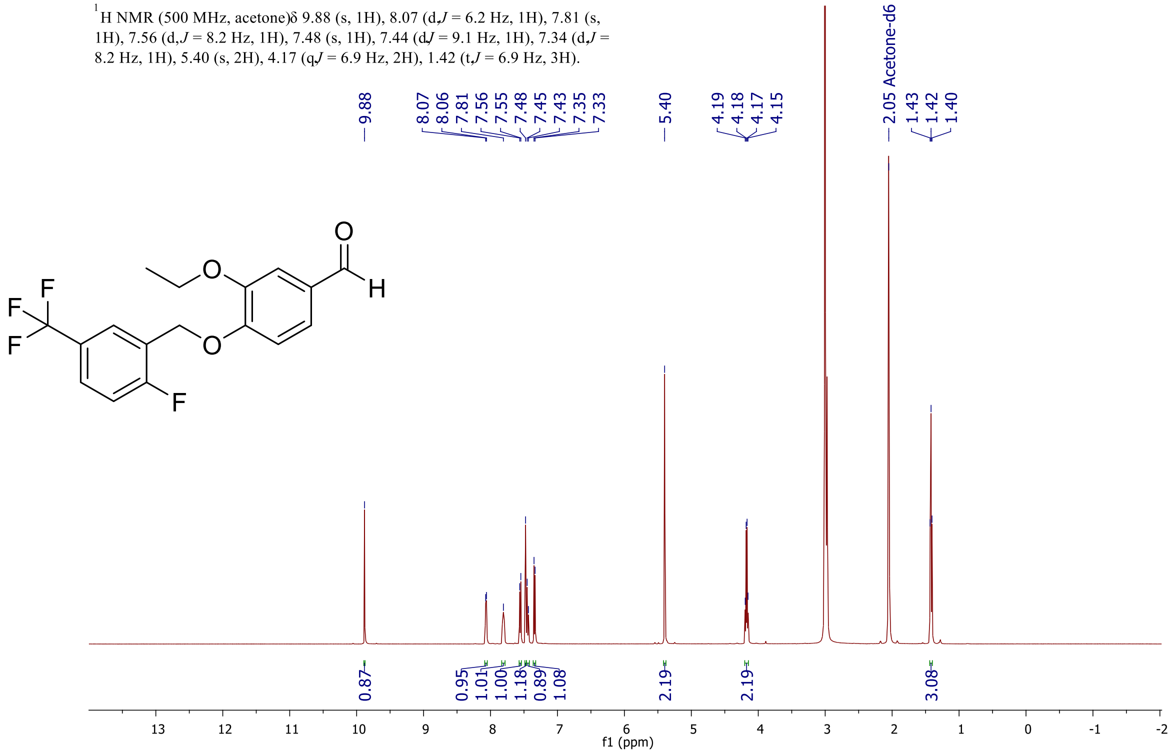

Figure SI 13 ^{1}H-NMR 500 MHz of 15 in acetone-d_6

^{13}C NMR (126 MHz, acetone) δ 191.52, 164.22, 162.22, 154.15, 150.50, 132.12, 128.45, 128.42, 128.37, 128.34, 128.07, 128.04, 127.99, 126.62, 126.49, 126.30, 117.37, 117.19, 114.47, 112.28, 65.17, 64.92, 14.94.

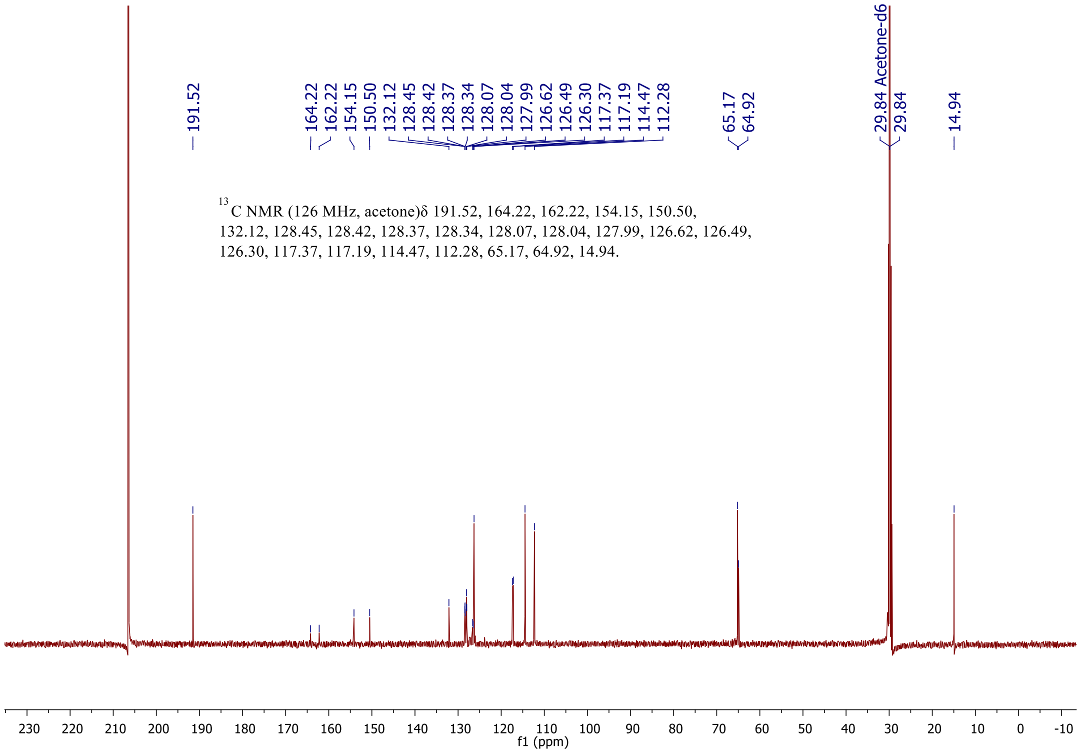

Figure SI 14 ^{13}CNMR 125 MHz of 15 in acetone-d_6.

4-((2,5-bis(trifluoromethyl)benzyl)oxy)benzaldehyde (**16**)

^{1}H NMR (500 MHz, acetone)δ 9.94 (s, 1H), 8.21 (s, 1H), 8.09 (d,*J* = 8.2 Hz, 1H), 7.99 (d,*J* = 8.2 Hz, 1H), 7.94 (d,*J* = 8.2 Hz, 2H), 7.29 (d,*J* = 8.2 Hz, 2H), 5.53 (s, 2H).

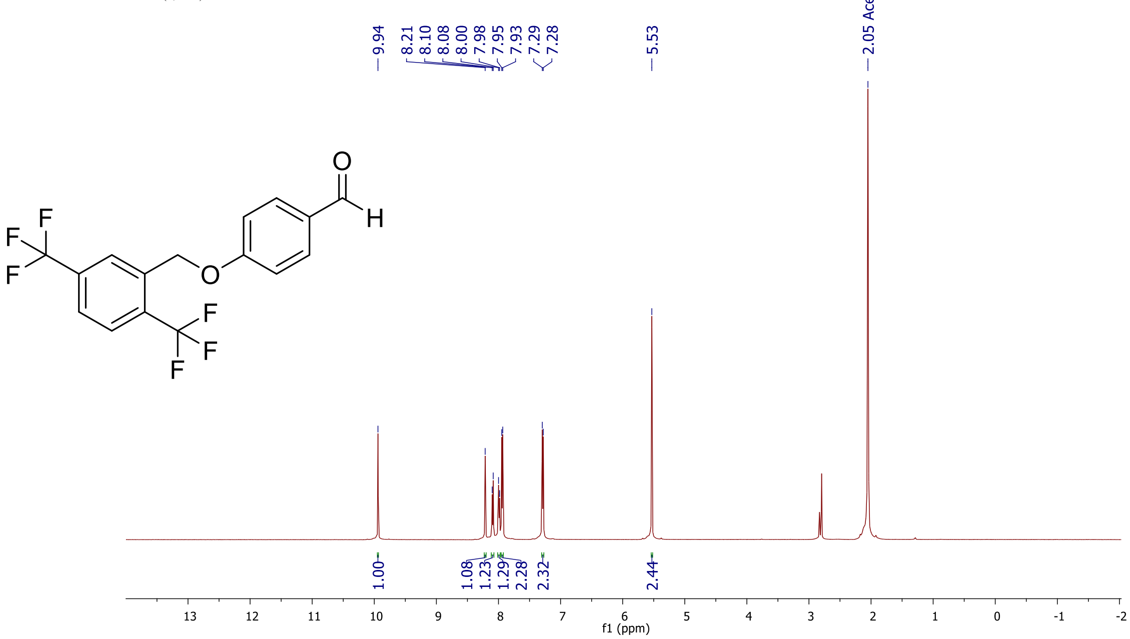

Figure SI 15 ^{1}H-NMR 500 MHz of 16 in acetone-d_6.

^{13}C NMR (126 MHz, acetone)δ 191.24, 163.80, 137.53, 134.87, 132.61, 131.95, 128.49, 128.45, 128.40, 127.65, 126.61, 125.76, 123.59, 123.35, 116.14, 116.11, 116.07, 66.97.

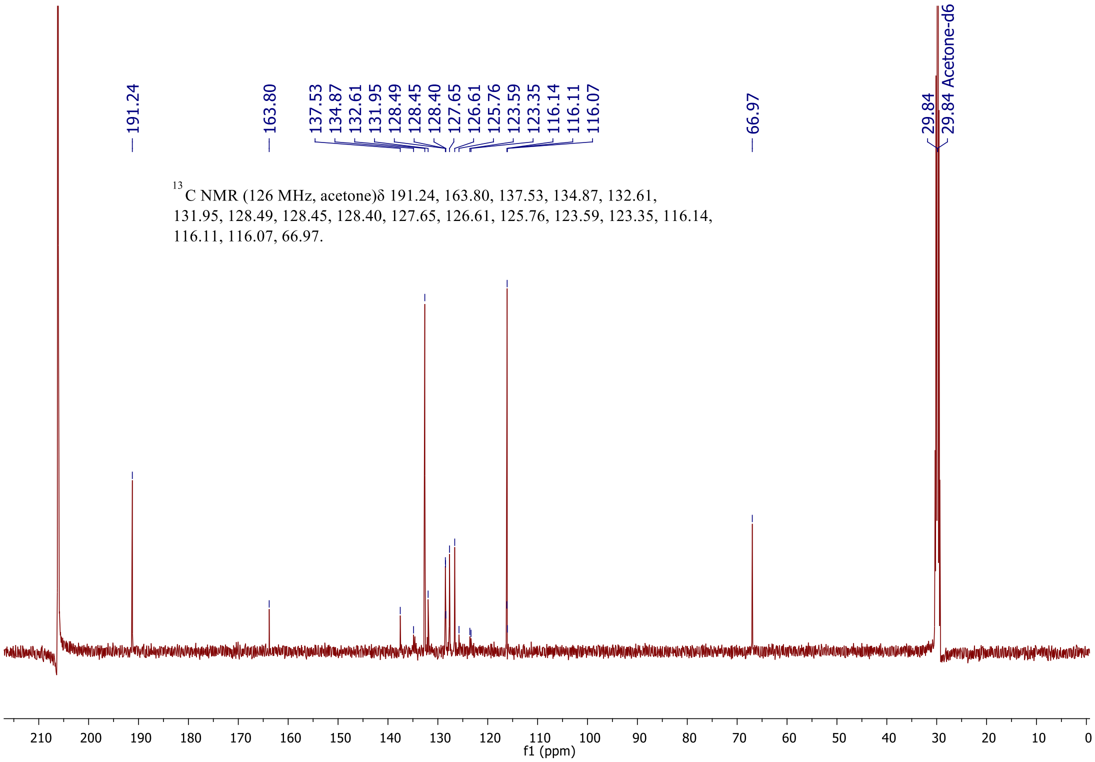

Figure SI 16 ^{13}CNMR 125 MHz of 16 in acetone-d_6.

4-((2,5-bis(trifluoromethyl)benzyl)oxy)-3-methoxybenzaldehyde (**17**)

^{1}H NMR (500 MHz, acetone)δ 9.90 (s, 1H), 8.29 (s, 1H), 8.07 (d,*J* = 8.2 Hz, 1H), 7.97 (d,*J* = 8.2 Hz, 1H), 7.57 (d,*J* = 8.1 Hz, 1H), 7.51 (s, 1H), 7.32 (d,*J* = 8.1 Hz, 1H), 5.52 (s, 2H), 3.96 (s, 3H).

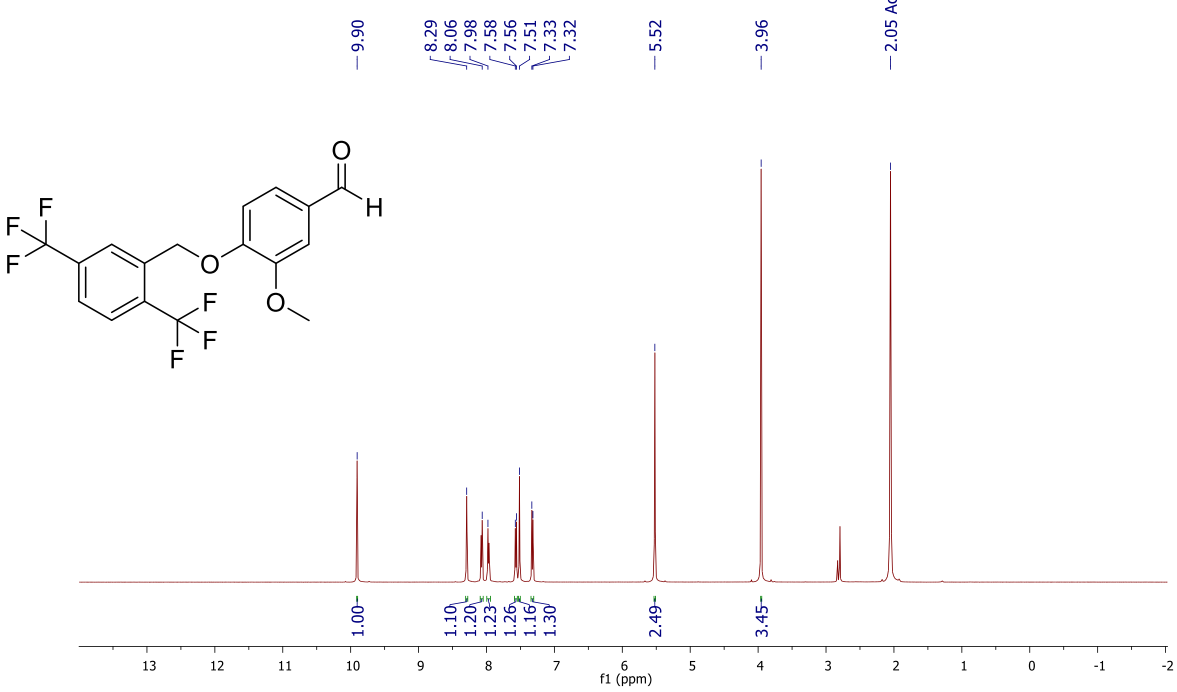

Figure SI 17 ^{1}H-NMR 500 MHz of 17 in acetone-d_6.

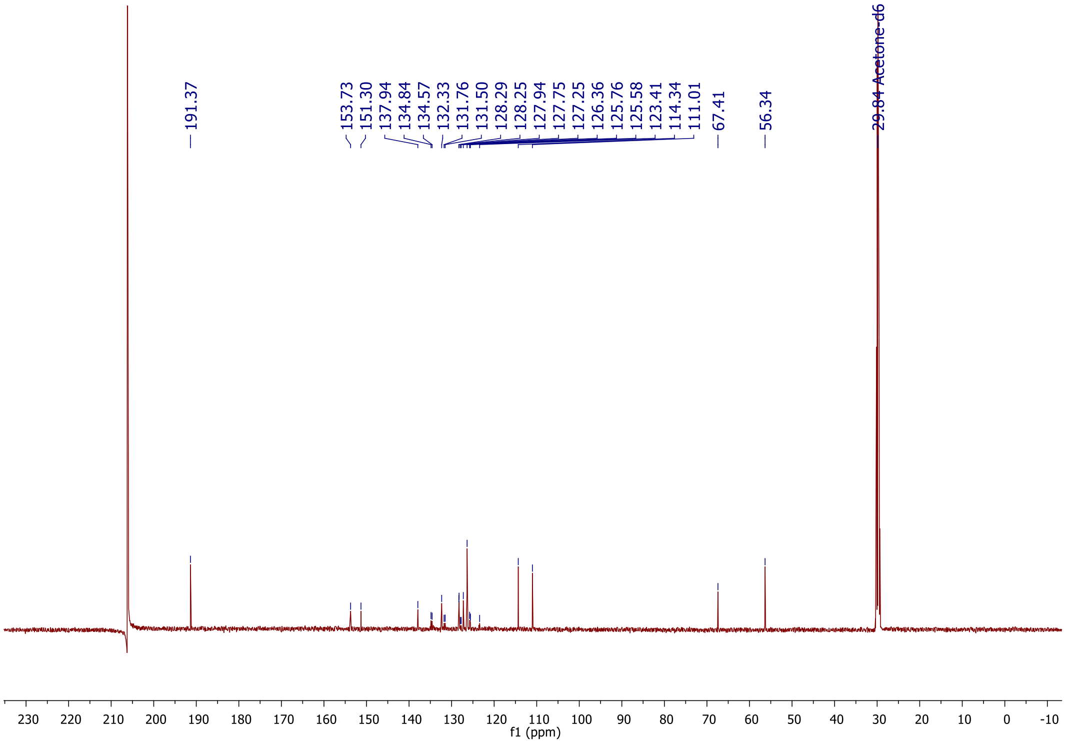

Figure SI 18 ^{13}CNMR 125 MHz of 17 in acetone-d_6.

3-((2,5-bis(trifluoromethyl)benzyl)oxy)-4-methoxybenzaldehyde (**18**)

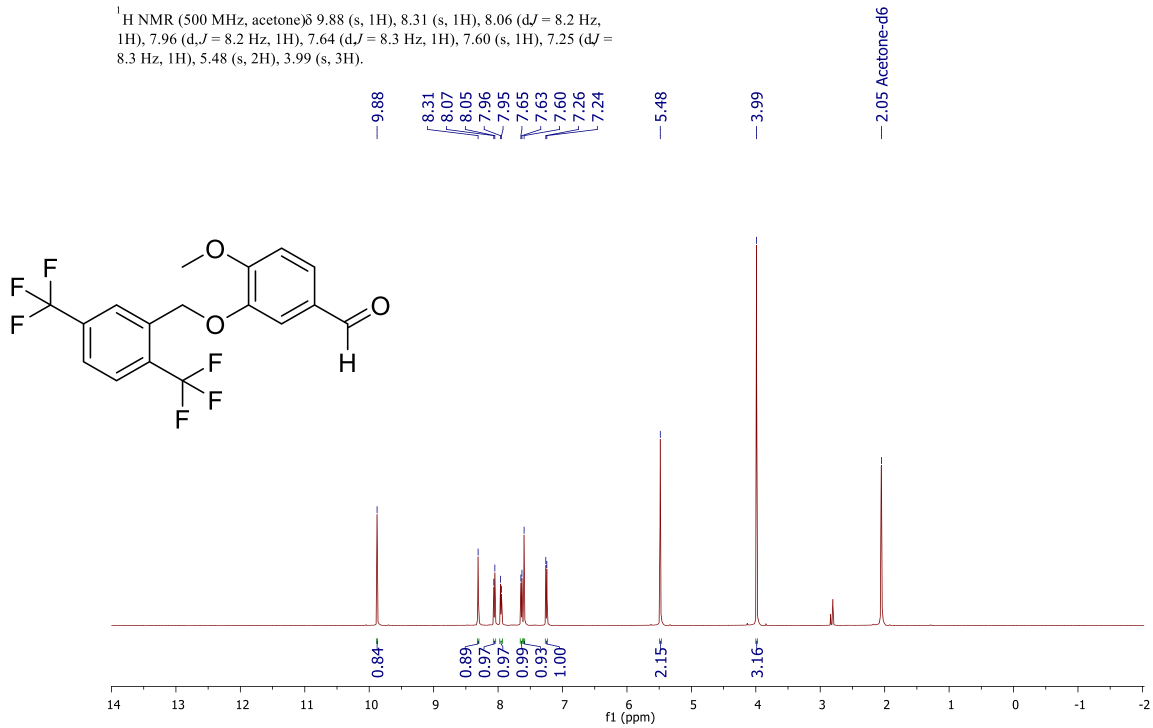

Figure SI 19 ^{1}H-NMR 500 MHz of 18 in acetone-d_6.

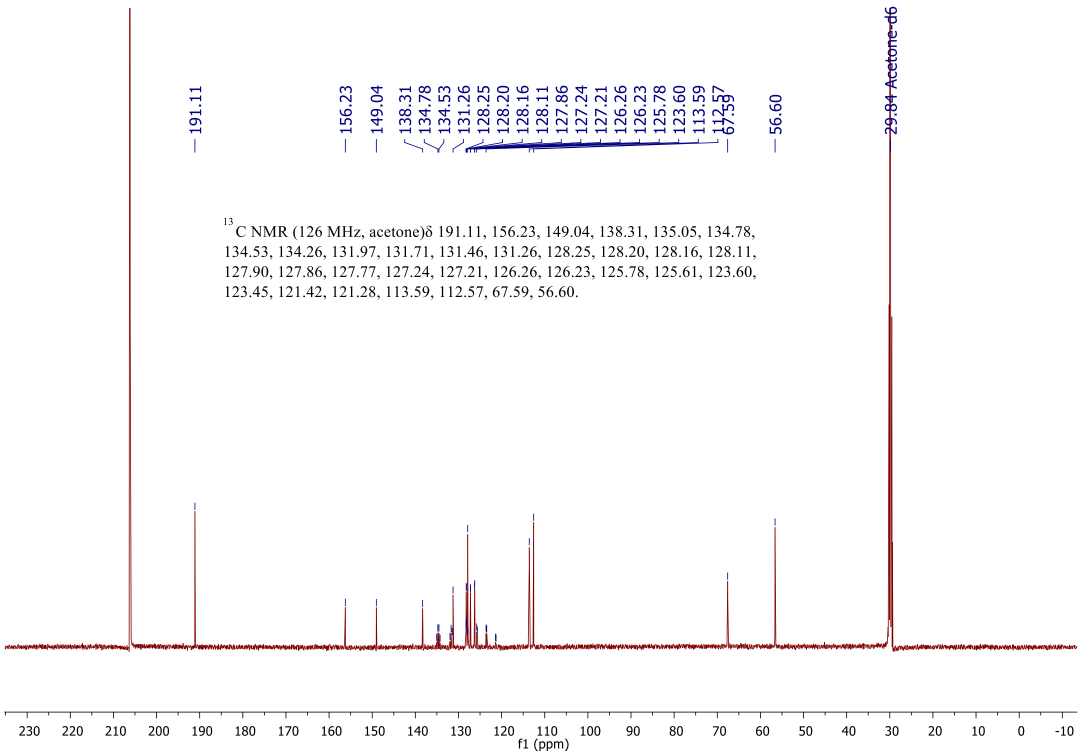

Figure SI 20 ^{13}CNMR 125 MHz of 18 in acetone-d_6.

4-((2,5-bis(trifluoromethyl)benzyl)oxy)-3-ethoxybenzaldehyde (**19**)

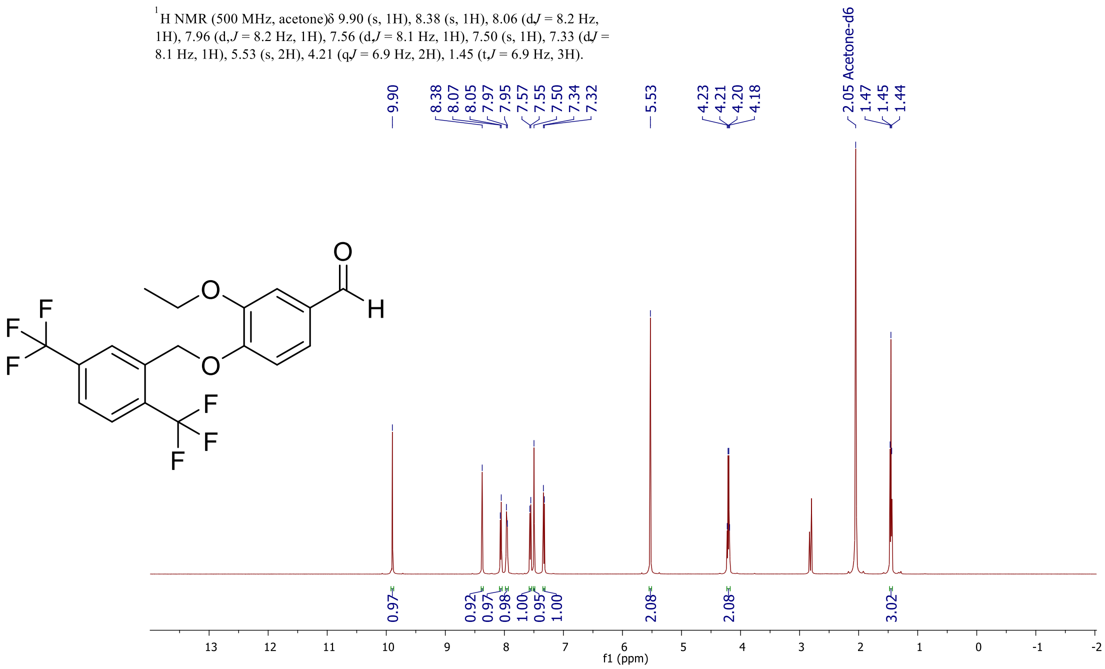

Figure SI 21 ^{1}H-NMR 500 MHz of 19 in acetone-d_6.

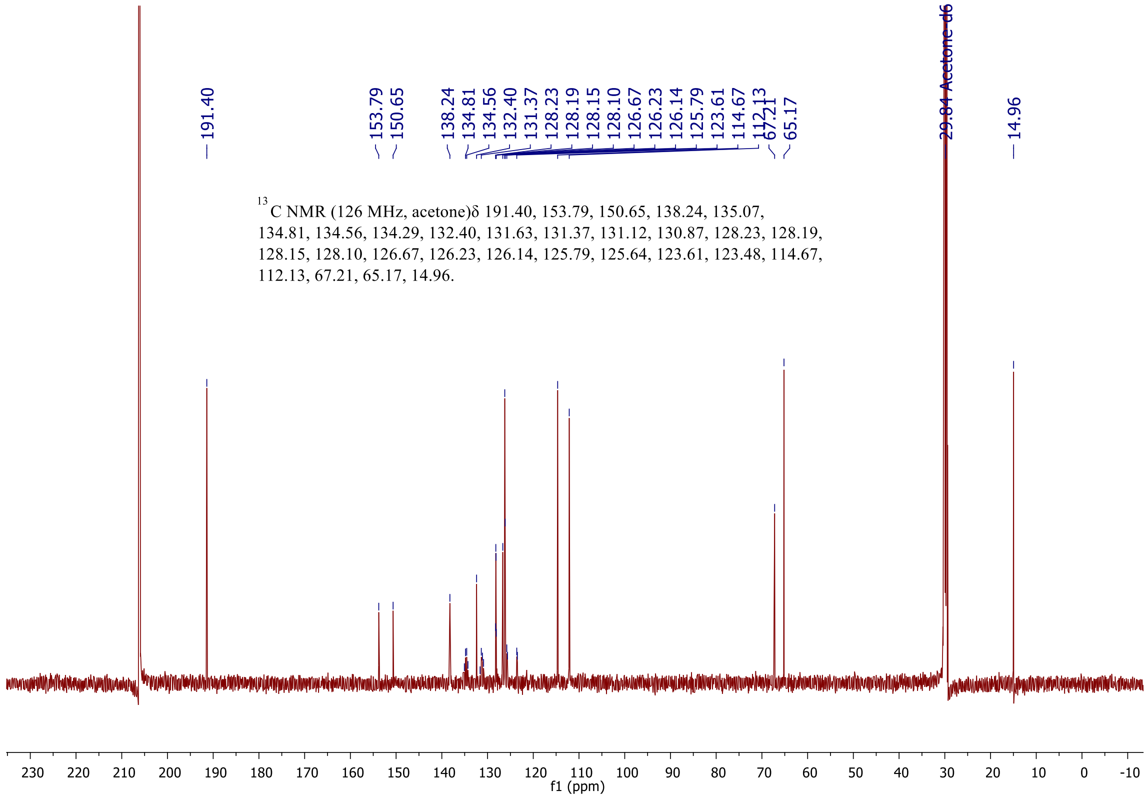

Figure SI 22 ^{13}CNMR 125 MHz of 19 in acetone-d_6.

2-(4-formyl-2-methoxyphenoxy)acetic acid (**20**)

^{1}H NMR (500 MHz, acetone)δ 9.88 (s, 1H), 7.52 (d, *J* = 8.2 Hz, 1H), 7.47 (s, 1H), 7.11 (d, *J* = 8.2 Hz, 1H), 4.88 (s, 2H), 3.92 (s, 3H).

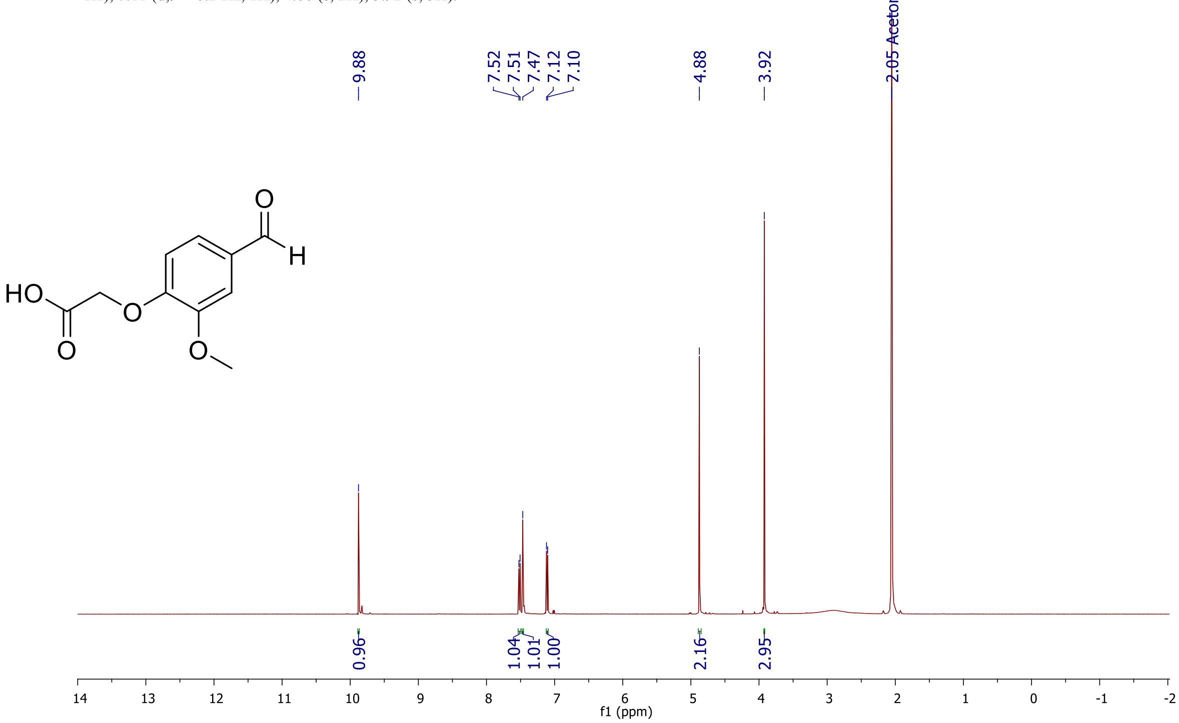

Figure SI 23 ^{1}H-NMR 500 MHz of 20 in acetone-d_6.

^{13}C NMR (126 MHz, acetone)δ 191.31, 169.63, 153.88, 150.95, 131.98, 126.18, 113.68, 111.16, 65.88, 56.26.

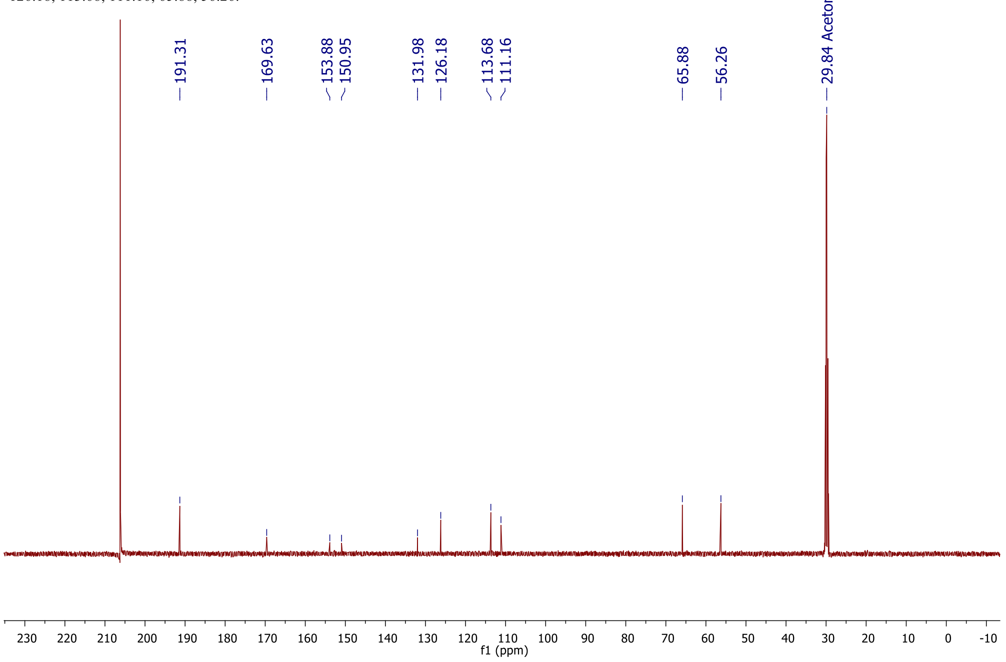

Figure SI 24 ^{13}CNMR 125 MHz of 20 in acetone-d_6.

(Z)-5-(4-(benzyloxy)benzylidene)imidazolidine-2,4-dione (**24**)

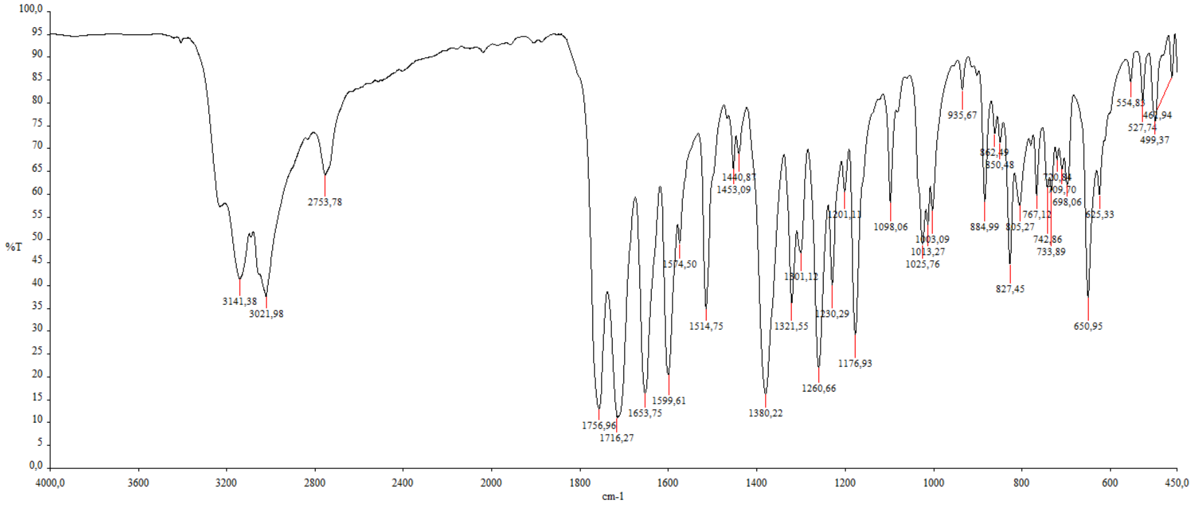

Figure SI 25 Infrared spectra of 24 in KBr pellets.

Elemental Composition Report **Page 1**

Single Mass Analysis
Tolerance = 10.0 PPM / DBE: min = -1.5, max = 50.0
Element prediction: Off
Number of isotope peaks used for i-FIT = 3

Monoisotopic Mass, Even Electron Ions
149 formula(e) evaluated with 1 results within limits (up to 3 best isotopic matches for each mass)
Elements Used:
C: 11-30 H: 0-17 N: 0-5 O: 0-10 I: 0-1

Naufal_BZO_CHO_HYD_pos 8 (0.119)
TOF MS ES+

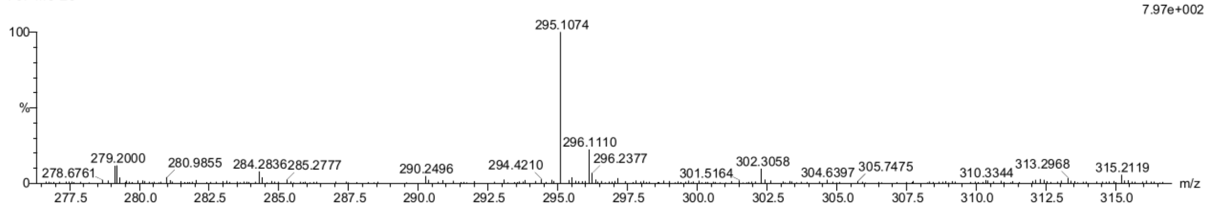

Figure SI 26 HR-TOF-MS-ES$^+$ spectra of 24.

1H NMR (500 MHz, dmso) δ 11.16 (s, 1H), 10.42 (s, 1H), 7.58 (d, *J* = 8.4 Hz, 2H), 7.45 (d, *J* = 7.5 Hz, 2H), 7.39 (t, *J* = 7.4 Hz, 2H), 7.33 (t, *J* = 7.1 Hz, 1H), 7.04 (d, *J* = 8.4 Hz, 2H), 6.39 (s, 1H), 5.15 (s, 2H)

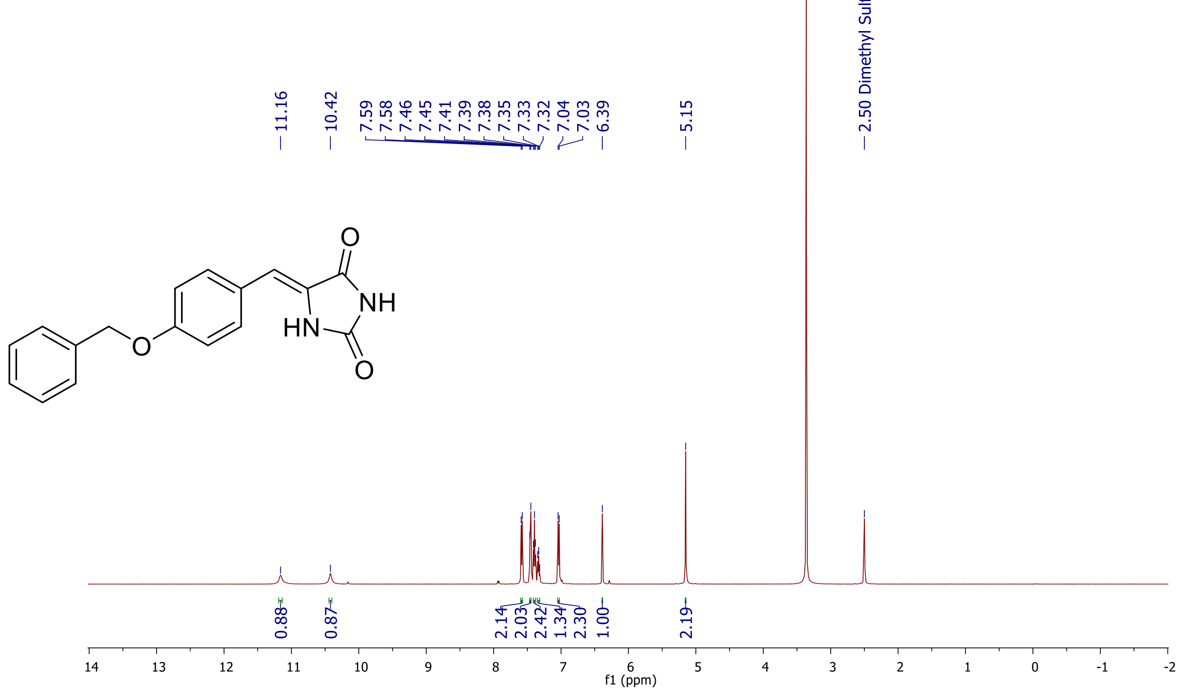

Figure SI 27 ^{1}H-NMR 500 MHz of 24 in dimethyl sulfoxide-d_6.

13C NMR (126 MHz, dmso) δ 165.63, 158.52, 155.64, 136.85, 131.10, 128.48, 127.92, 127.73, 126.18, 125.67, 115.17, 108.62, 69.31, 39.52.

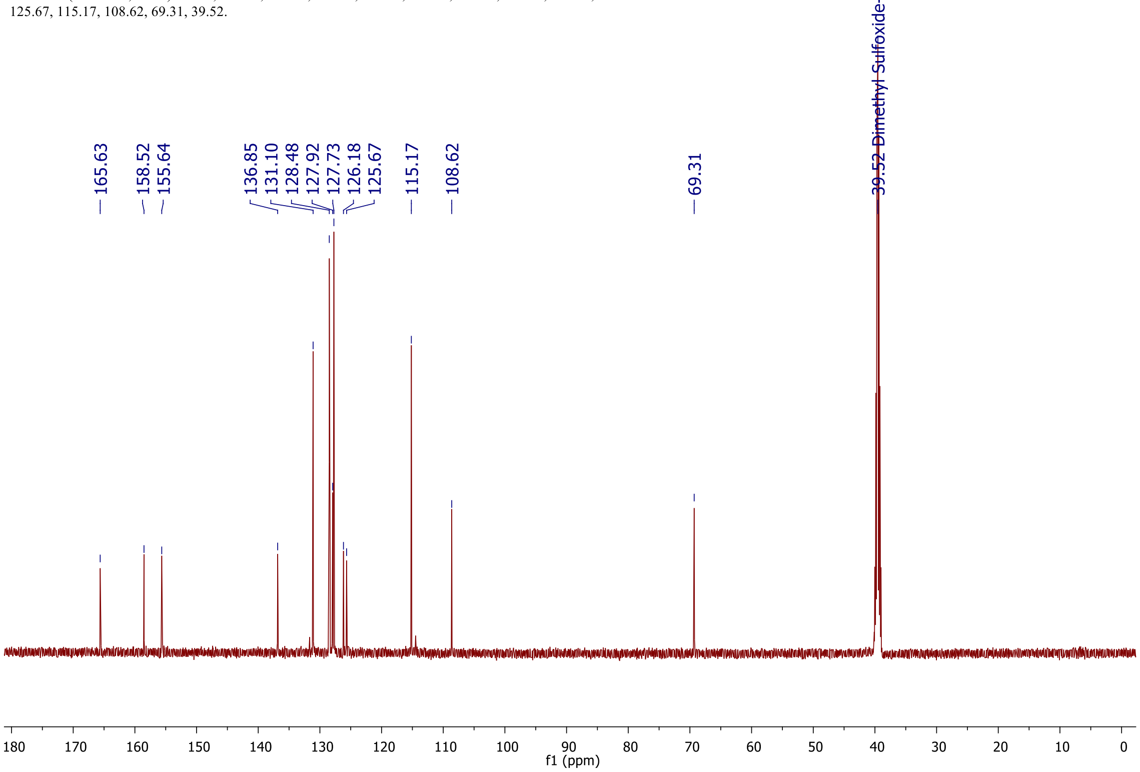

Figure SI 28 ^{13}CNMR 125 MHz of 24 in dimethyl sulfoxide-d_6.

(Z)-5-(4-(benzyloxy)-3-methoxybenzylidene)imidazolidine-2,4-dione (**25**)

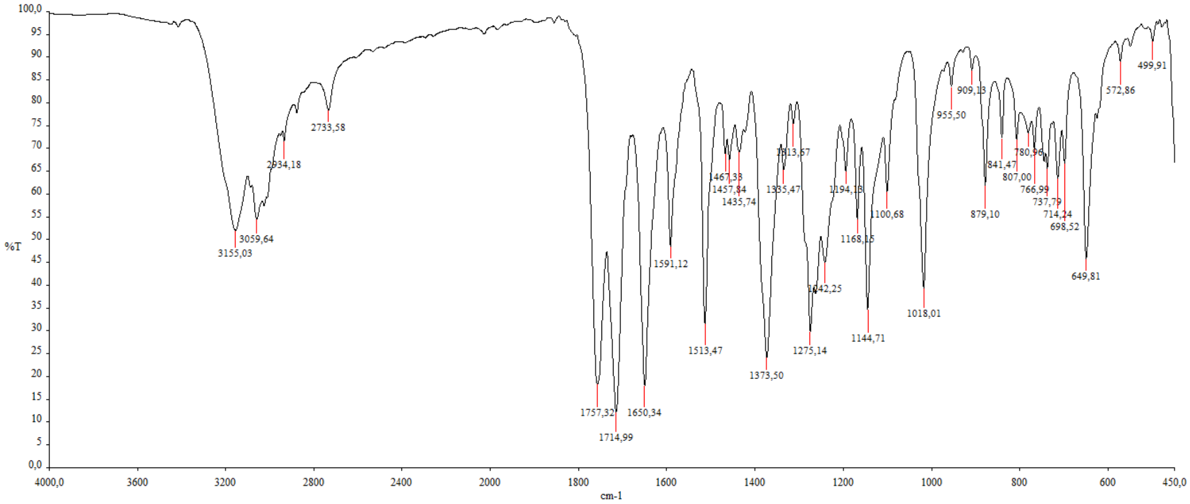

Figure SI 29 Infrared spectra of 25 in KBr pellets.

Elemental Composition Report **Page 1**

Single Mass Analysis
Tolerance = 10.0 PPM / DBE: min = -1.5, max = 50.0
Element prediction: Off
Number of isotope peaks used for i-FIT = 3

Monoisotopic Mass, Even Electron Ions
128 formula(e) evaluated with 1 results within limits (up to 3 best isotopic matches for each mass)
Elements Used:
C: 0-20 H: 0-40 N: 0-5 O: 0-7
Naufal_Bzovanhyd_neg 21 (0.339)
TOF MS ES-

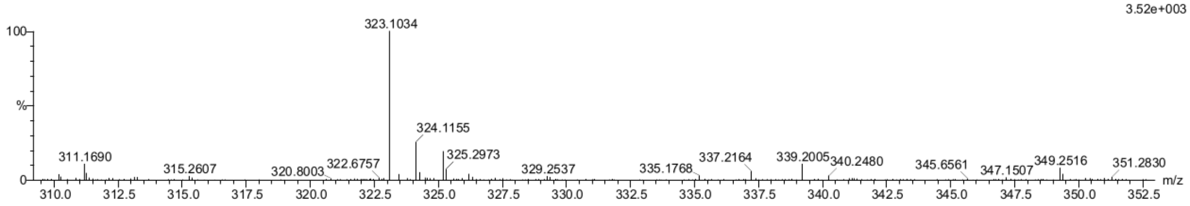

Figure SI 30 HR-TOF-MS-ES^{-} spectra of 25.

^{1}H NMR (500 MHz, dmso) δ 11.17 (s, 1H), 10.49 (s, 1H), 7.45 (d, *J* = 7.4 Hz, 2H), 7.40 (t, *J* = 7.0 Hz, 2H), 7.34 (t, *J* = 7.1 Hz, 1H), 7.18 – 7.15 (m, 2H), 7.05 (d, *J* = 7.8 Hz, 1H), 6.39 (s, 1H), 5.13 (s, 2H), 3.84 (s, 3H).

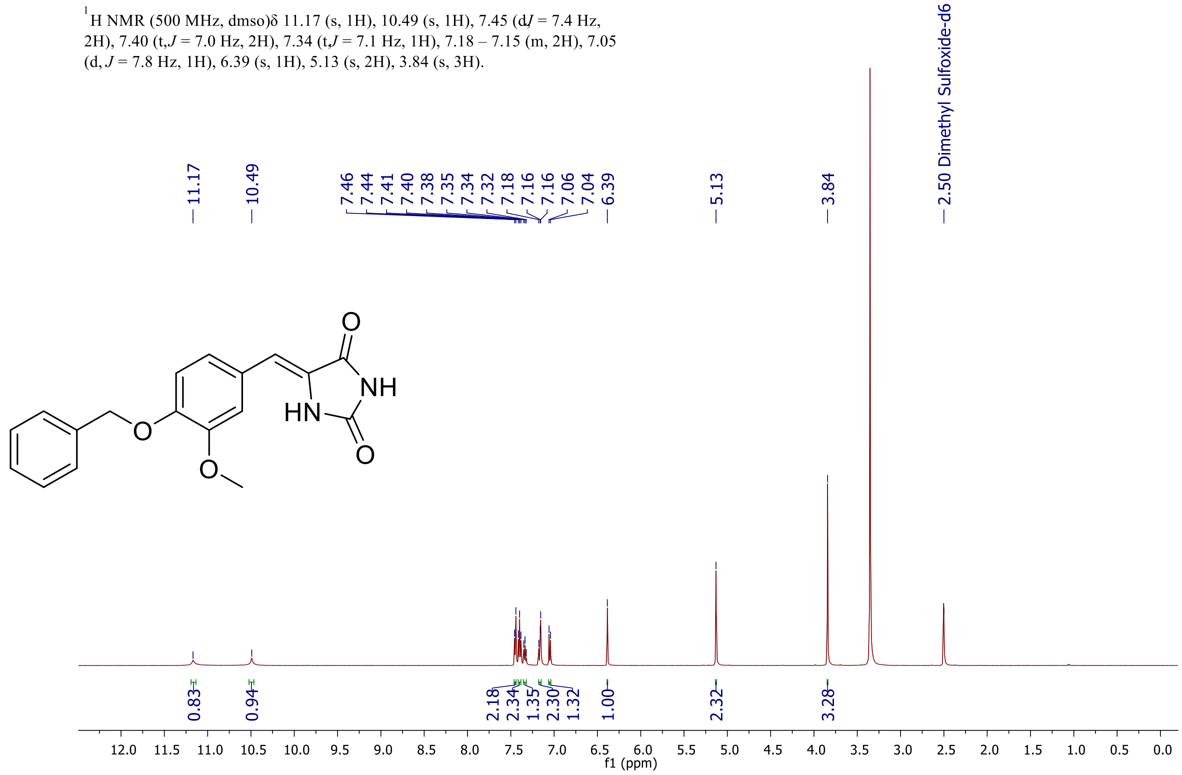

Figure SI 31 ^{1}H-NMR 500 MHz of 25 in dimethyl sulfoxide-d_6.

^{13}C NMR (126 MHz, dmso) δ 165.61, 155.75, 149.12, 148.29, 136.91, 128.44, 127.91, 127.81, 126.25, 126.01, 122.86, 113.44, 112.90, 109.11, 69.82, 55.77, 39.52.

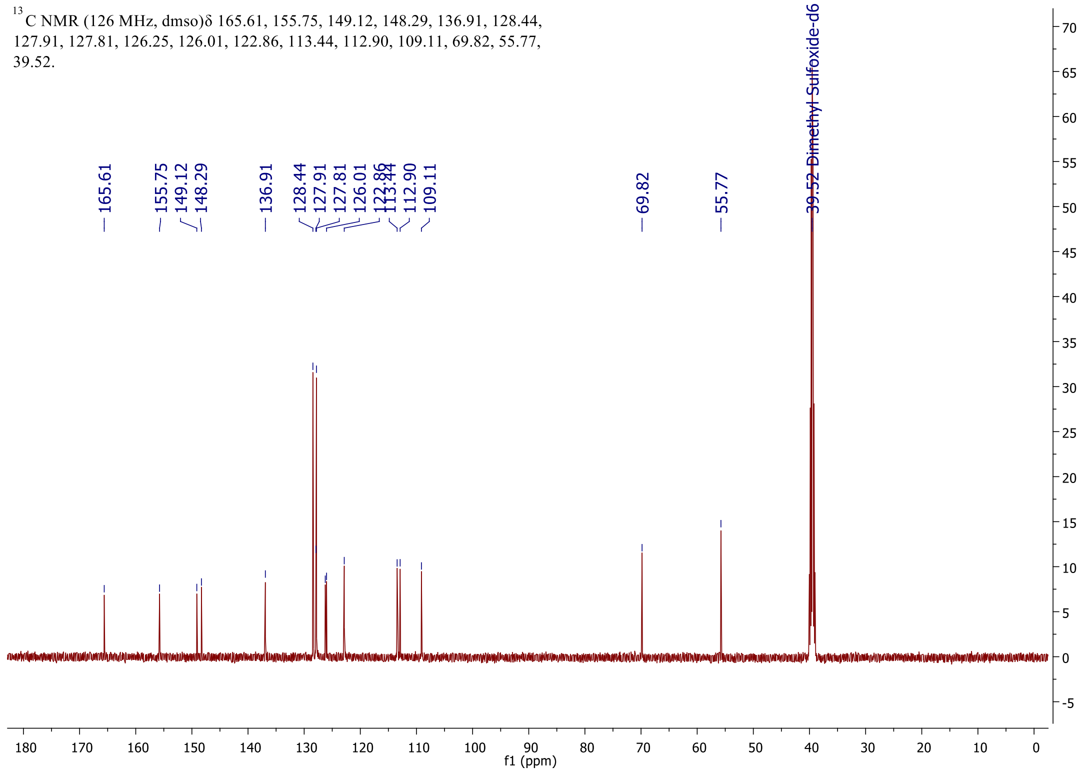

Figure SI 32 ^{13}CNMR 125 MHz of 25 in dimethyl sulfoxide-d_6.

(Z)-5-(4-(benzyloxy)-3-ethoxybenzylidene)imidazolidine-2,4-dione **26**

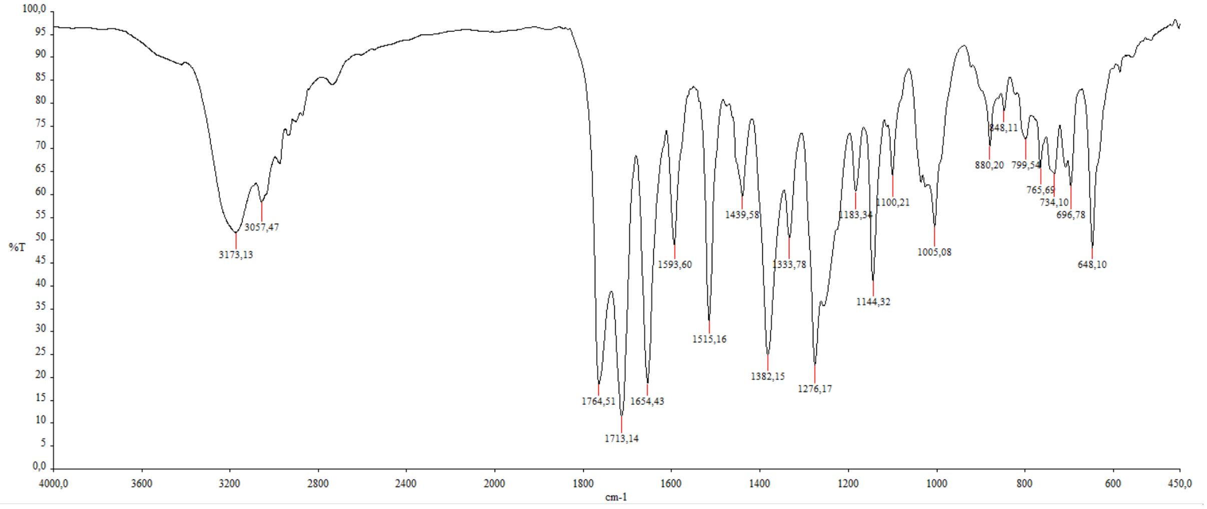

Figure SI 33 Infrared spectra of 26 in KBr pellets.

Elemental Composition Report **Page 1**

Single Mass Analysis
Tolerance = 10.0 PPM / DBE: min = -1.5, max = 50.0
Element prediction: Off
Number of isotope peaks used for i-FIT = 3

Monoisotopic Mass, Even Electron Ions
116 formula(e) evaluated with 1 results within limits (up to 3 best isotopic matches for each mass)
Elements Used:
C: 0-20 H: 0-40 N: 0-5 O: 0-7
Naufal_BZOEVanHyd_neg 101 (1.697) Cm (99:104)
TOF MS ES-

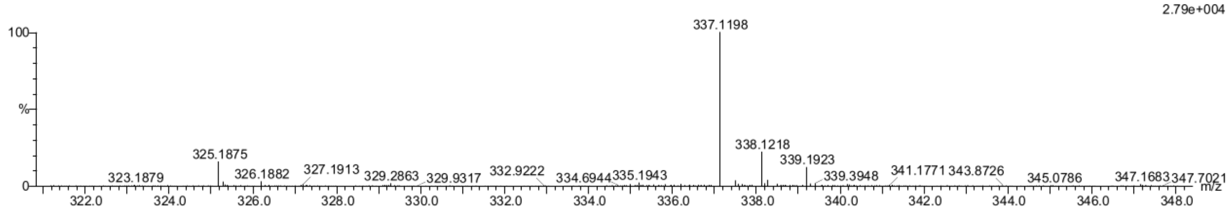

Figure SI 34 HR-TOF-MS-ES^{-} spectra of 26.

^{1}H NMR (500 MHz, dmso) δ 11.08 (s, 1H), 10.52 (s, 1H), 7.45 (d, *J* = 7.2 Hz, 2H), 7.39 (t, *J* = 7.3 Hz, 2H), 7.32 (t, *J* = 6.9 Hz, 1H), 7.15 (s, 2H), 7.03 (d, *J* = 8.8 Hz, 1H), 6.37 (s, 1H), 5.15 (s, 2H), 4.12 (q, *J* = 13.7, 6.8 Hz, 2H), 1.33 (t, *J* = 6.8 Hz, 3H).

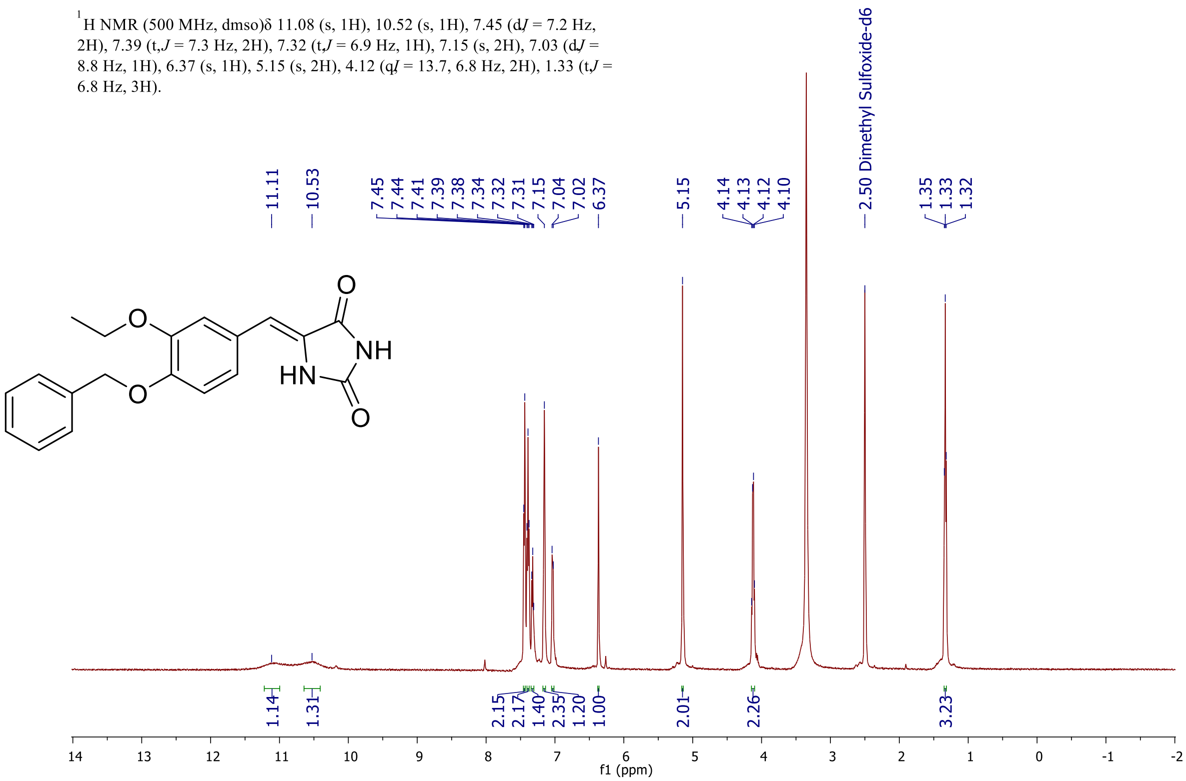

Figure SI 35 ^{1}H-NMR 500 MHz of 26 in dimethyl sulfoxide-d_6.

^{13}C NMR (126 MHz, dmso) δ 165.63, 155.76, 148.53, 148.47, 137.09, 128.44, 127.82, 127.56, 126.24, 126.13, 122.94, 114.39, 114.01, 109.09, 69.85, 64.06, 39.52, 14.76.

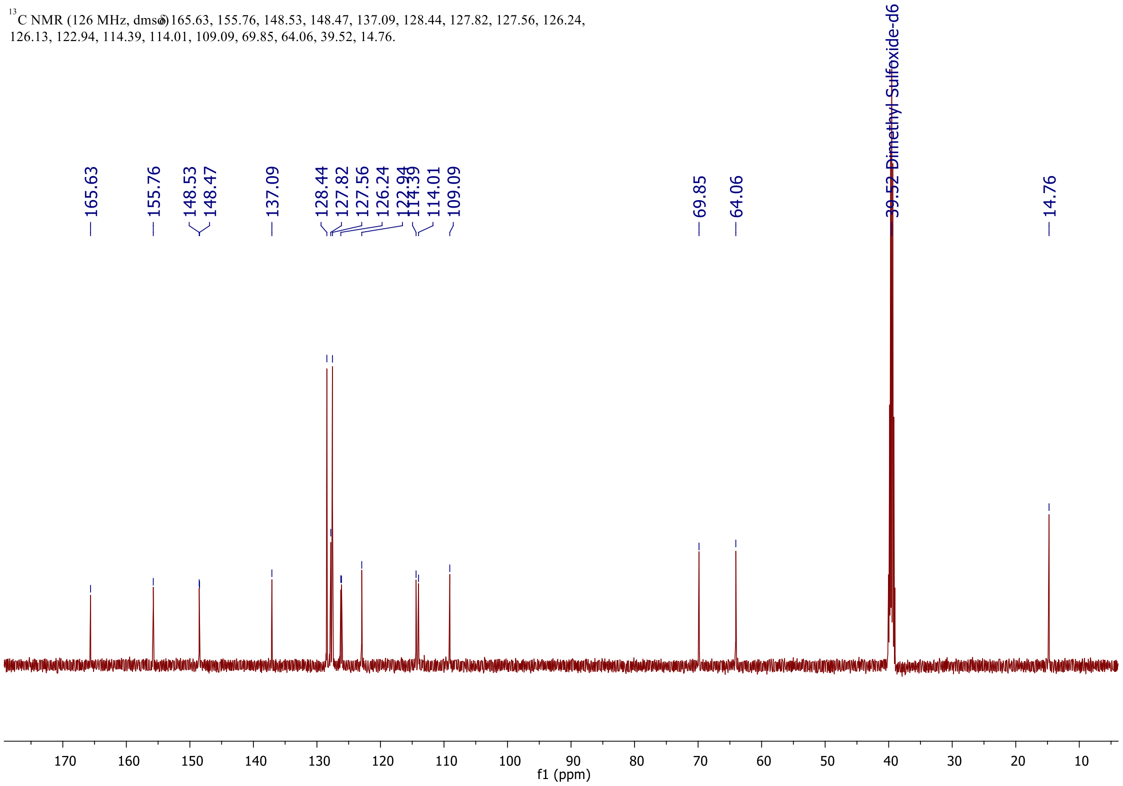

Figure SI 36 ^{13}CNMR 125 MHz of 26 in dimethyl sulfoxide-d_6.

(Z)-5-(4-((2-fluoro-5-(trifluoromethyl)benzyl)oxy)benzylidene)imidazolidine-2,4-dione (**27**)

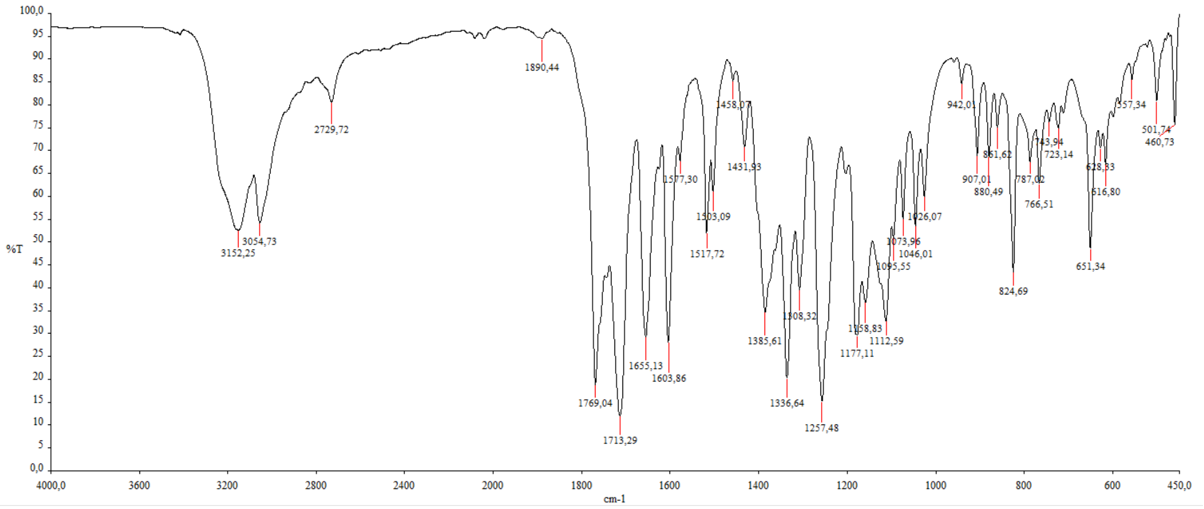

Figure SI 37 Infrared spectra of 27 in KBr pellets.

Elemental Composition Report **Page 1**

Single Mass Analysis
Tolerance = 10.0 PPM / DBE: min = -1.5, max = 50.0
Element prediction: Off
Number of isotope peaks used for i-FIT = 3

Monoisotopic Mass, Even Electron Ions
60 formula(e) evaluated with 1 results within limits (up to 3 best isotopic matches for each mass)
Elements Used:
C: 0-18 H: 0-30 N: 0-3 O: 0-5 F: 3-4

Naufal_2fcf3ochobhyd_neg 18 (0.288)
TOF MS ES-

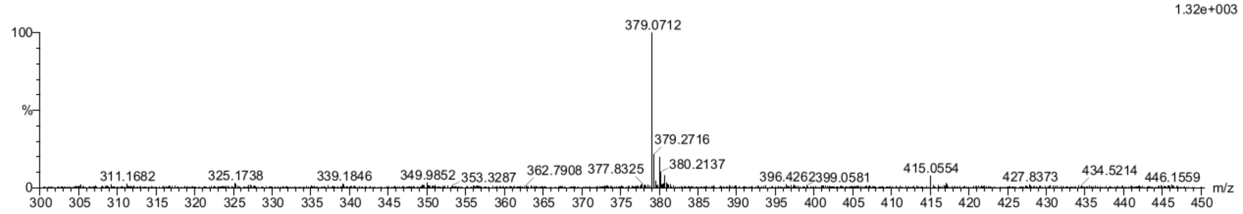

Figure SI 38 HR-TOF-MS-ES$^-$ spectra of 27.

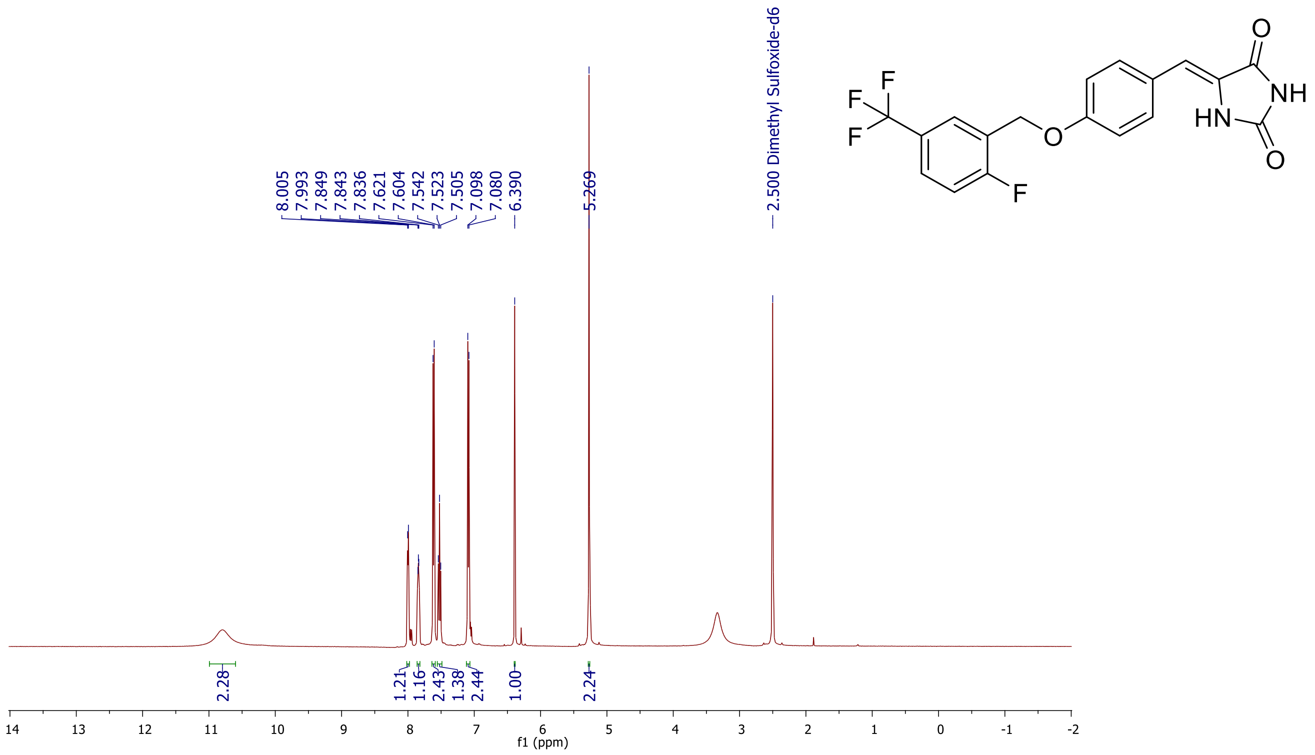

Figure SI 39 ^{1}H-NMR 500 MHz of 27 in dimethyl sulfoxide-d_6.

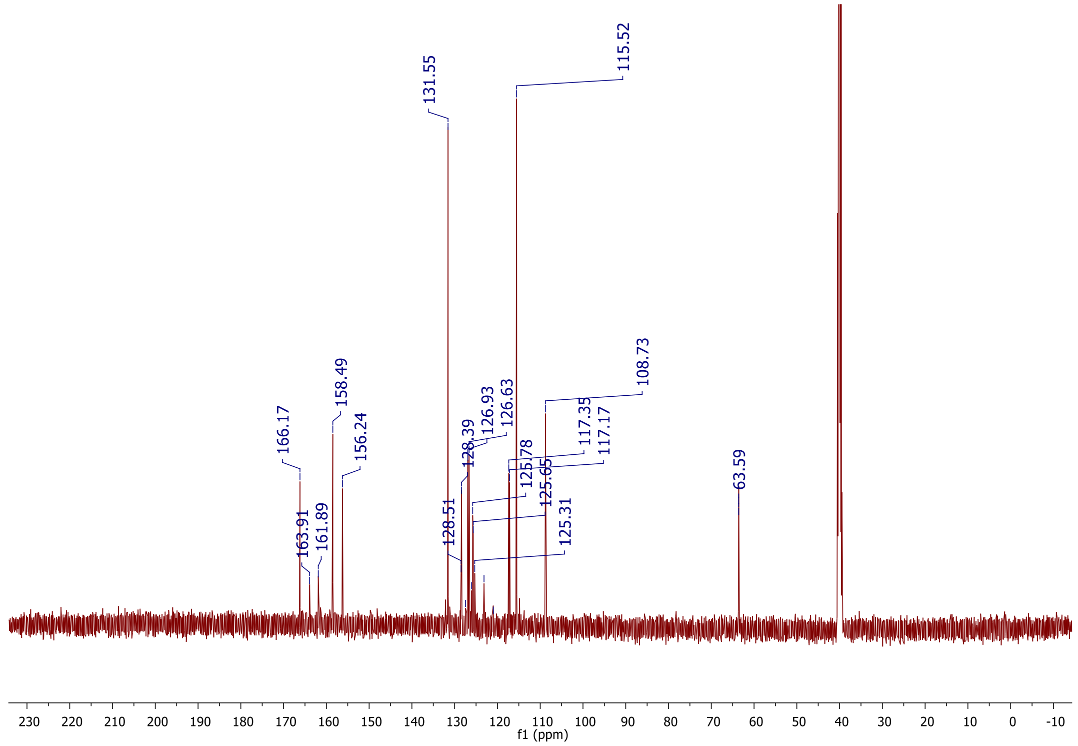

Figure SI 40 ^{13}CNMR 125 MHz of 27 in dimethyl sulfoxide-d_6.

(Z)-5-(4-((2-fluoro-5-(trifluoromethyl)benzyl)oxy)-3-methoxybenzylidene)imidazolidine-2,4-dione (**28**)

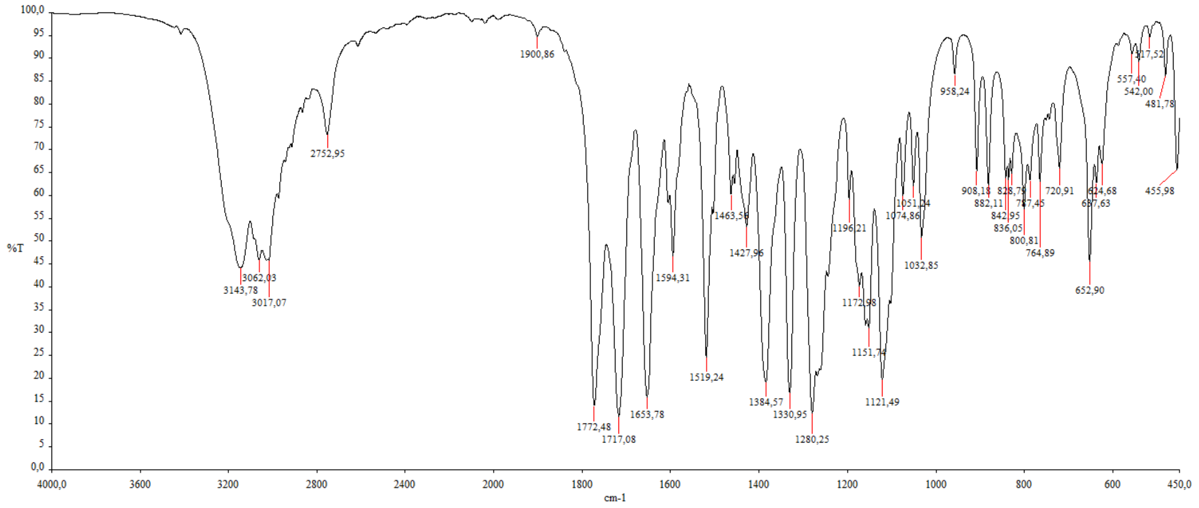

Figure SI 41 Infrared spectra of 28 in KBr pellets.

Elemental Composition Report **Page 1**

Single Mass Analysis
Tolerance = 10.0 PPM / DBE: min = -1.5, max = 50.0
Element prediction: Off
Number of isotope peaks used for i-FIT = 3

Monoisotopic Mass, Even Electron Ions
109 formula(e) evaluated with 1 results within limits (up to 3 best isotopic matches for each mass)
Elements Used:
C: 1-20 H: 1-20 N: 1-5 O: 1-5 F: 0-4

Naufal_2F5CF3OVHYD_neg 16 (0.255)
TOF MS ES-

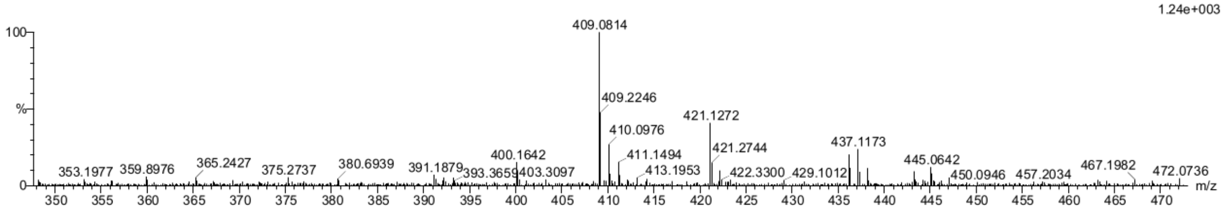

Figure SI 42 HR-TOF-MS-ES- spectra of 28.

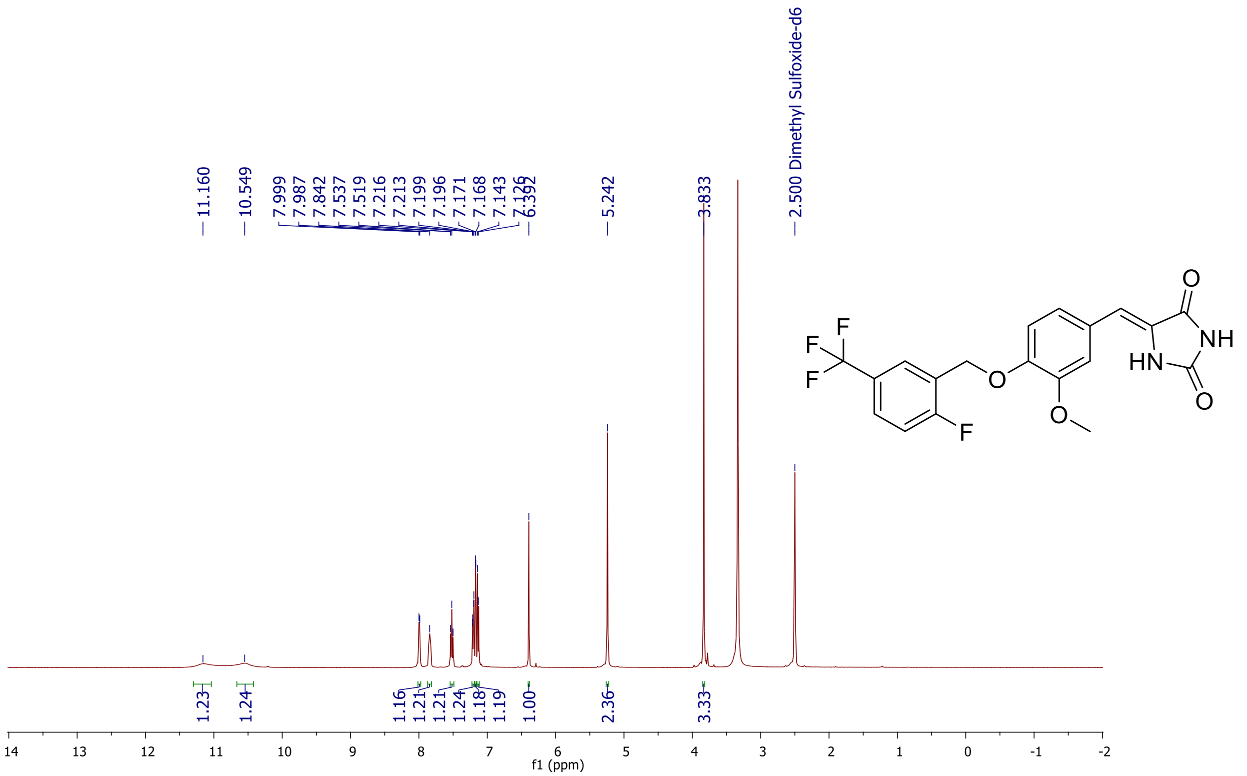

Figure SI 43 ^{1}H-NMR 500 MHz of 28 in dimethyl sulfoxide-*d*$_6$.

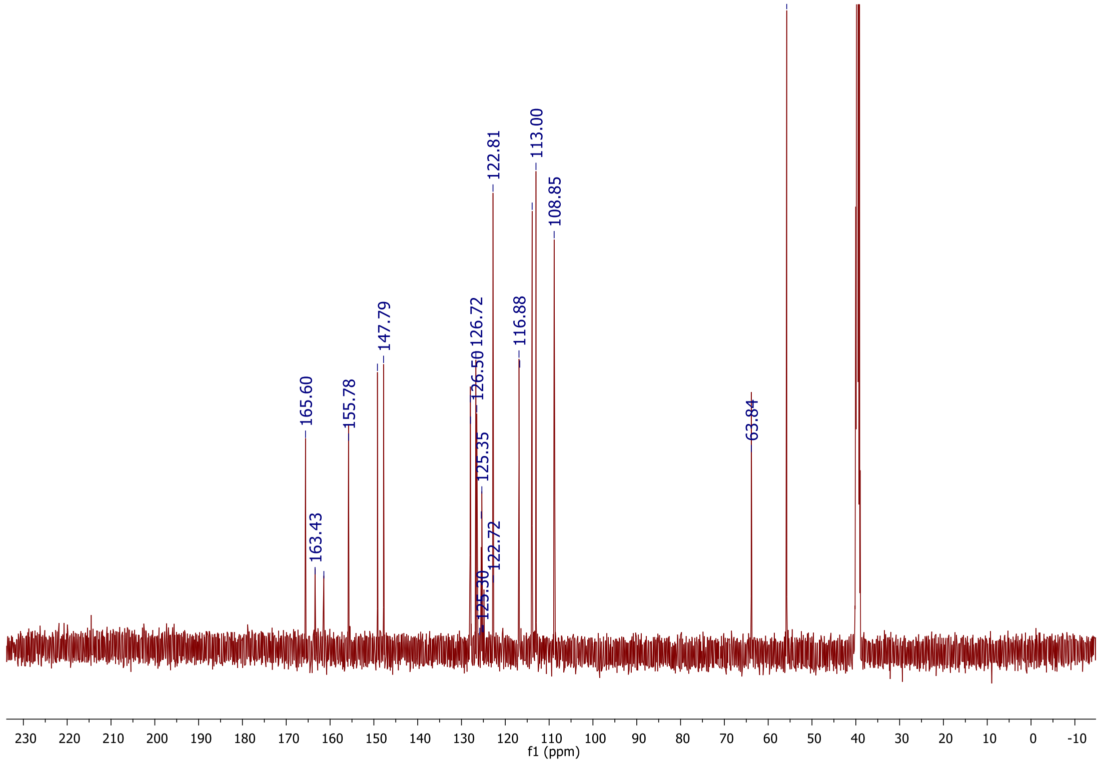

Figure SI 44 ^{13}CNMR 125 MHz of 28 in dimethyl sulfoxide-*d*$_6$.

(Z)-5-(3-((2-fluoro-5-(trifluoromethyl)benzyl)oxy)-4-methoxybenzylidene)imidazolidine-2,4-dione (**29**)

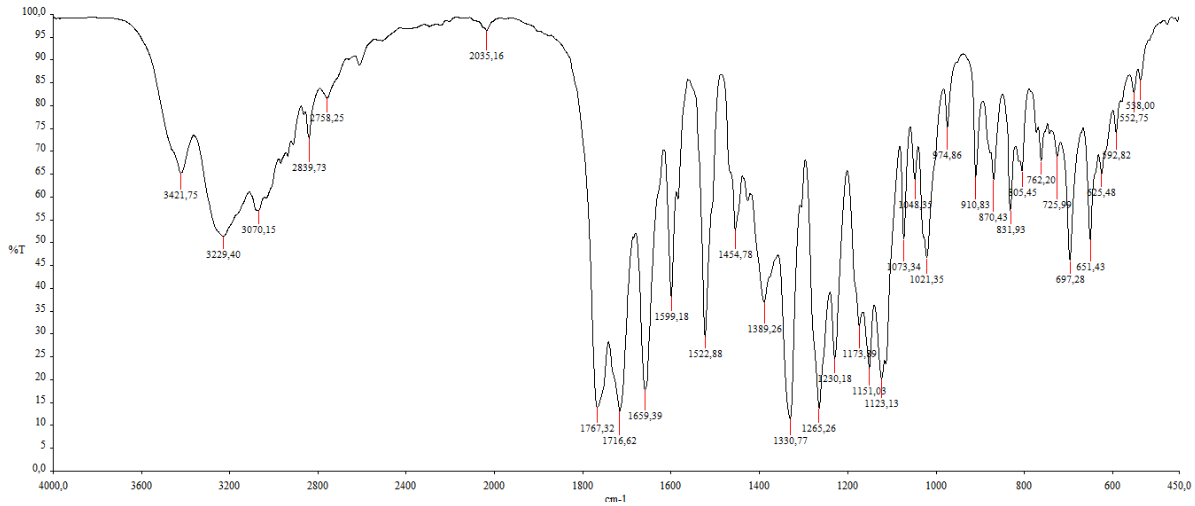

Figure SI 45 Infrared spectra of 29 in KBr pellets.

Elemental Composition Report **Page 1**

Single Mass Analysis
Tolerance = 10.0 PPM / DBE: min = -1.5, max = 50.0
Element prediction: Off
Number of isotope peaks used for i-FIT = 3

Monoisotopic Mass, Even Electron Ions
55 formula(e) evaluated with 1 results within limits (up to 3 best isotopic matches for each mass)
Elements Used:
C: 0-20 H: 0-30 N: 0-3 O: 0-5 F: 3-4

Naufal_2fcf3oivhyd_neg 22 (0.356)
TOF MS ES-

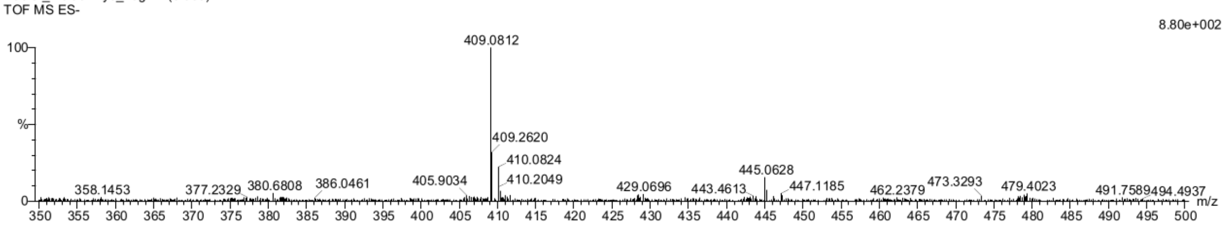

Figure SI 46 HR-TOF-MS-ES- spectra of 29.

^{1}H NMR (500 MHz, dmso) δ 11.18 (s, 1H), 10.54 (s, 1H), 8.00 (d, J = 6.0 Hz, 1H), 7.84 (s, 1H), 7.53 (t, J = 9.0 Hz, 1H), 7.34 (s, 1H), 7.26 (d, J = 8.4 Hz, 1H), 7.03 (d, J = 8.4 Hz, 1H), 6.39 (s, 1H), 5.31 (s, 2H), 3.80 (s, 3H).

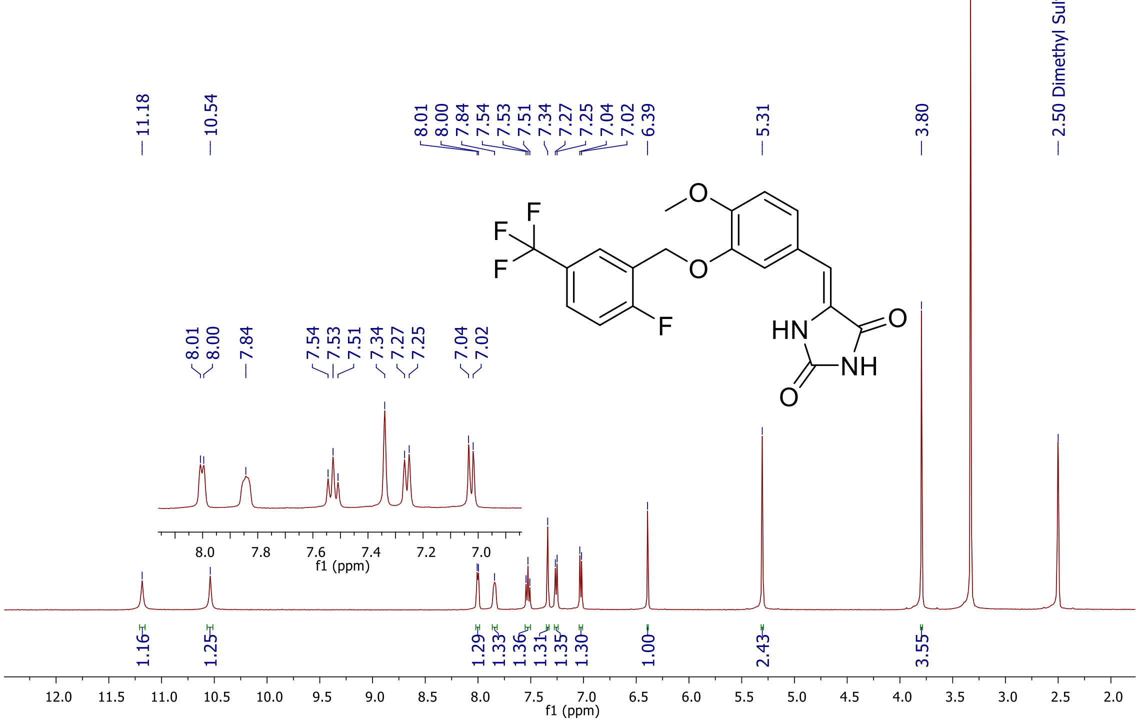

Figure SI 47 ^{1}H-NMR 500 MHz of 29 in dimethyl sulfoxide-d_6.

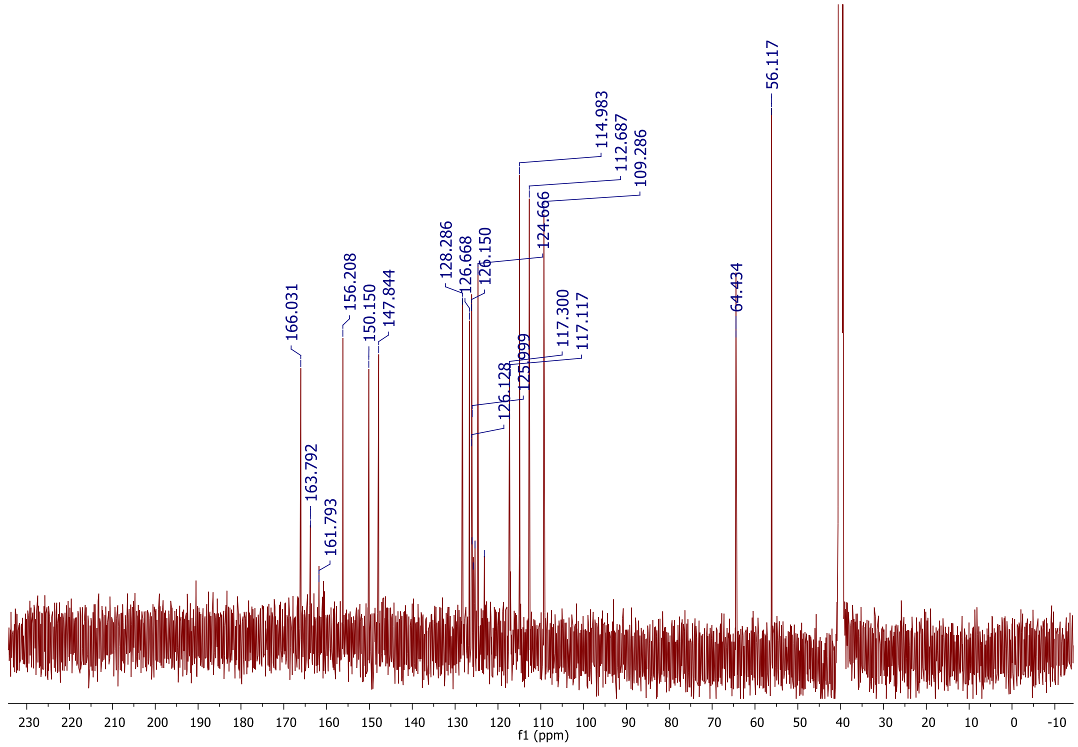

Figure SI 48 ^{13}CNMR 125 MHz of 29 in dimethyl sulfoxide-d_6.

(Z)-5-(3-ethoxy-4-((2-fluoro-5-(trifluoromethyl)benzyl)oxy)benzylidene)imidazolidine-2,4-dione (**30**)

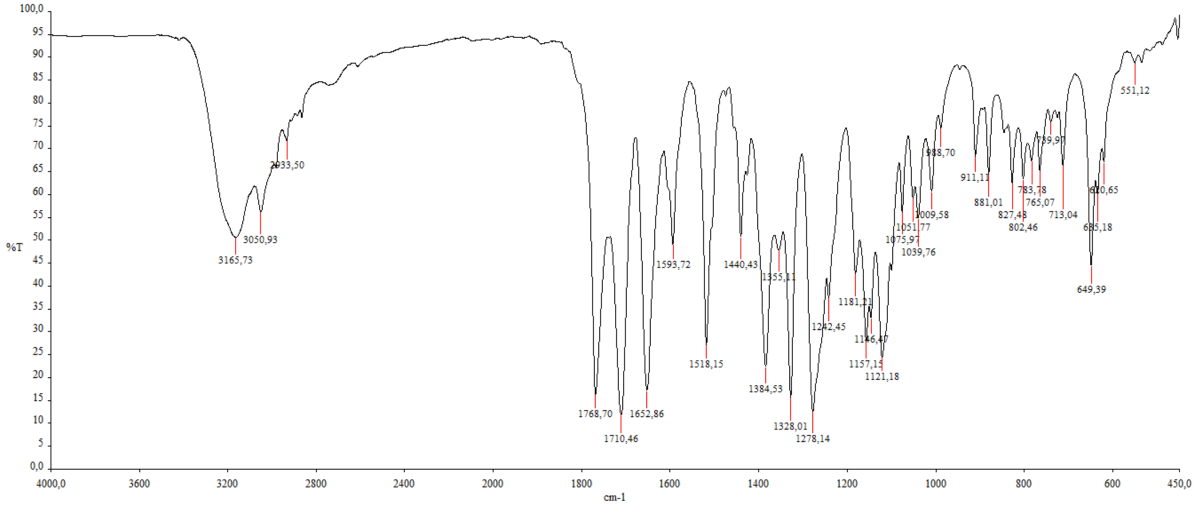

Figure SI 49 Infrared spectra of 24 in KBr pellets.

Elemental Composition Report **Page 1**

Single Mass Analysis
Tolerance = 10.0 PPM / DBE: min = -1.5, max = 50.0
Element prediction: Off
Number of isotope peaks used for i-FIT = 3

Monoisotopic Mass, Even Electron Ions
103 formula(e) evaluated with 1 results within limits (up to 3 best isotopic matches for each mass)
Elements Used:
C: 1-20 H: 1-20 N: 1-5 O: 1-5 F: 0-4

Naufal_2fcf3evihyd_neg 43 (0.713) Cm (42:43)
TOF MS ES-

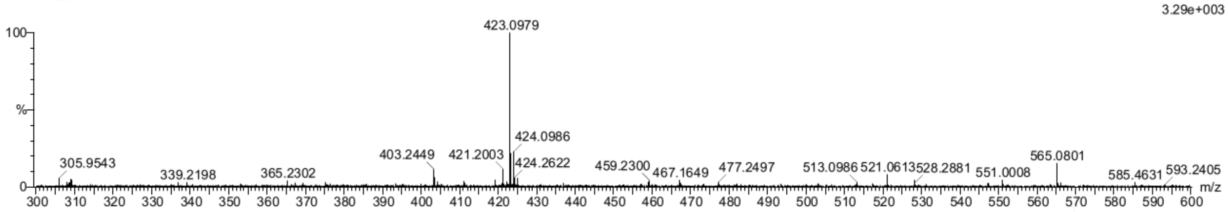

Figure SI 50 HR-TOF-MS-ES- spectra of 30.

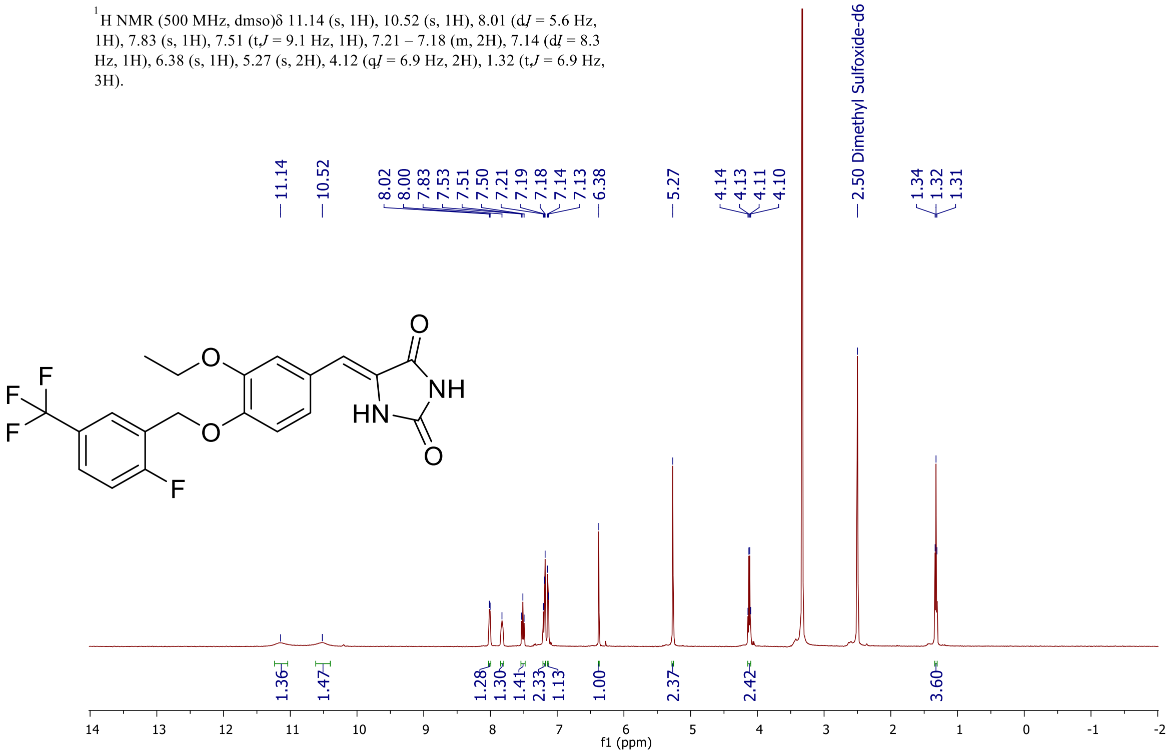

Figure SI 51 ^{1}H-NMR 500 MHz of 30 in dimethyl sulfoxide-d_6.

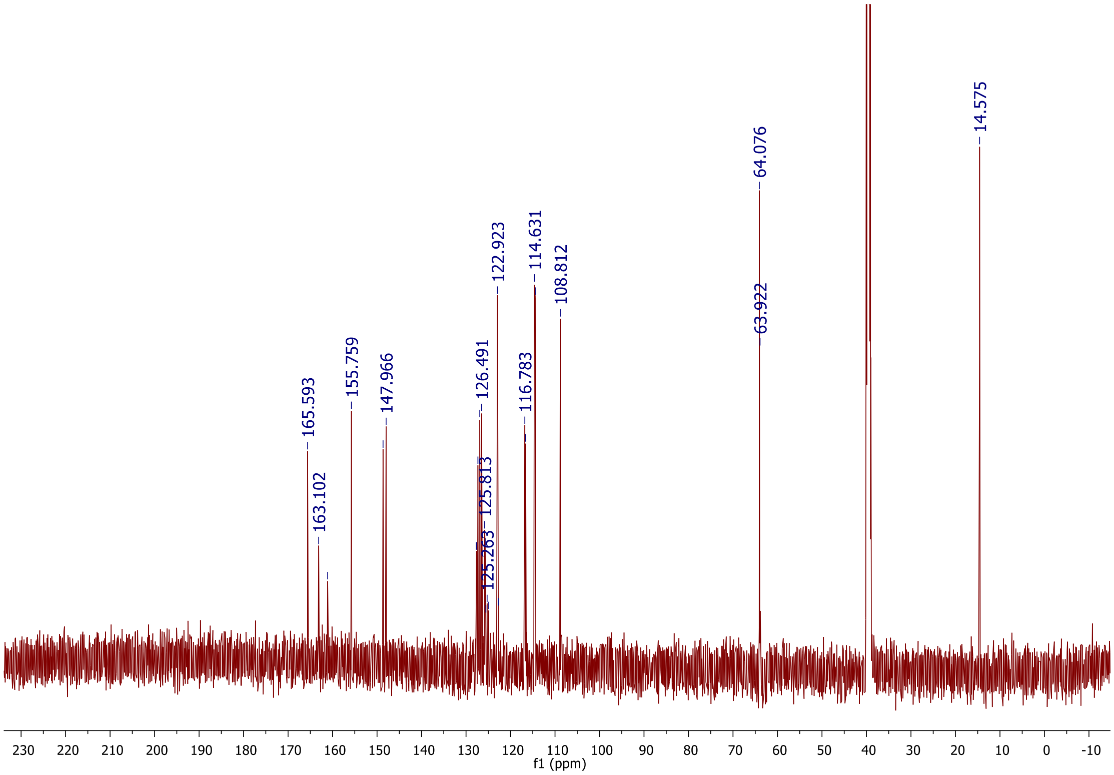

Figure SI 52 ^{13}CNMR 125 MHz of 30 in dimethyl sulfoxide-d_6.

(Z)-5-(4-((2,5-bis(trifluoromethyl)benzyl)oxy)benzylidene)imidazolidine-2,4-dione (**31**)

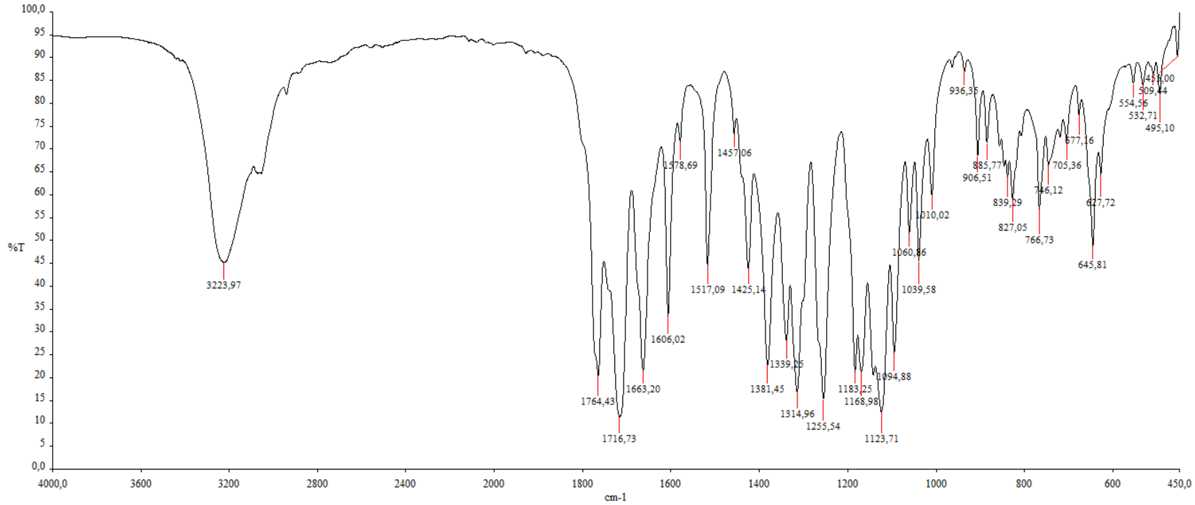

Figure SI 53 Infrared spectra of 31 in KBr pellets.

Elemental Composition Report **Page 1**

Single Mass Analysis
Tolerance = 10.0 PPM / DBE: min = -1.5, max = 50.0
Element prediction: Off
Number of isotope peaks used for i-FIT = 3

Monoisotopic Mass, Even Electron Ions
212 formula(e) evaluated with 1 results within limits (up to 3 best isotopic matches for each mass)
Elements Used:
C: 0-20 H: 0-30 N: 0-5 O: 0-5 F: 3-6

Naufal_biscf3ochohyd_neg 9 (0.136) Cm (9:11)
TOF MS ES-

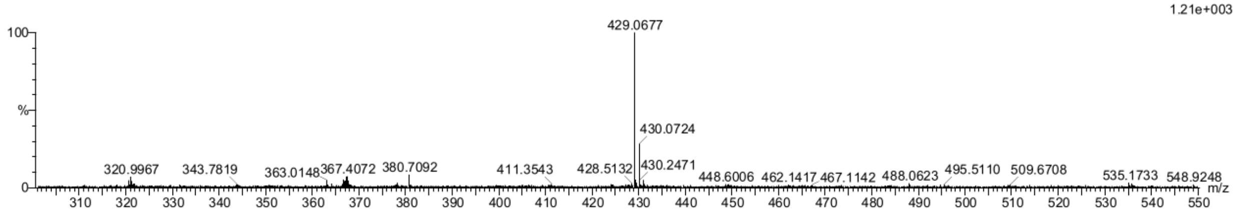

Figure SI 54 HR-TOF-MS-ES- spectra of 31.

^{1}H NMR (500 MHz, dmso)δ 11.14 (s, 1H), 10.48 (s, 1H), 8.17 (s, 1H), 8.07 (d, J = 8.2 Hz, 1H), 8.00 (d, J = 8.2 Hz, 1H), 7.62 (d, J = 8.7 Hz, 2H), 7.07 (d, J = 8.7 Hz, 2H), 6.39 (s, 1H), 5.37 (s, 2H).

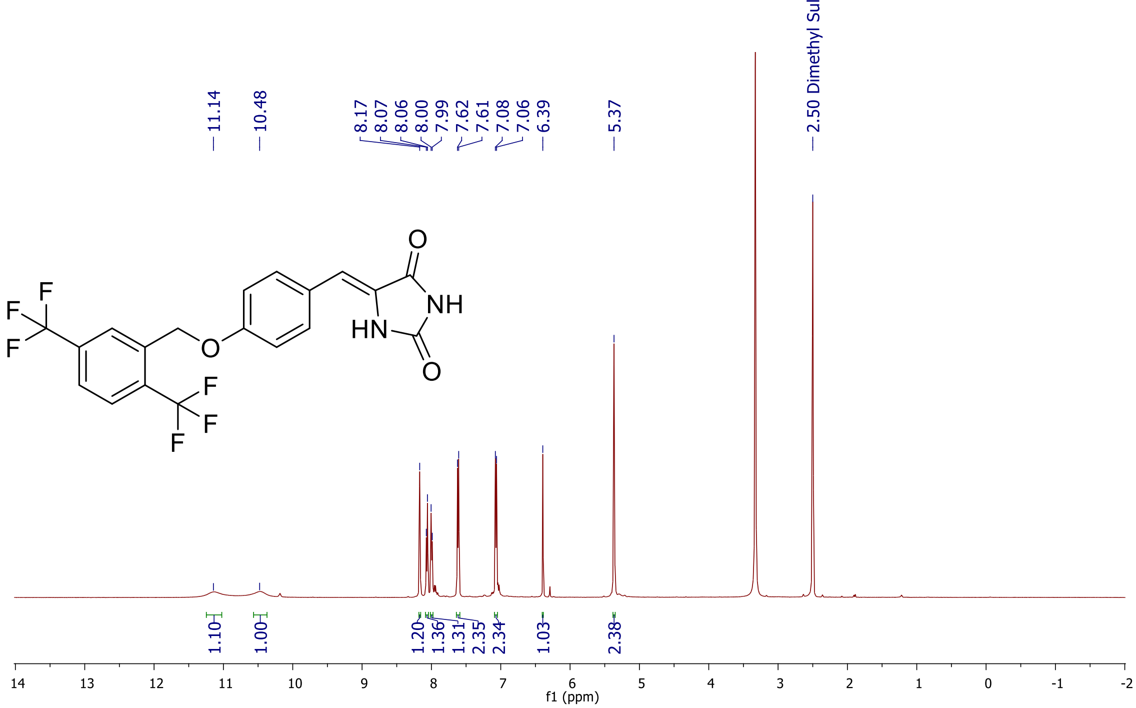

Figure SI 55 ^{1}H-NMR 500 MHz of 31 in dimethyl sulfoxide-d_6.

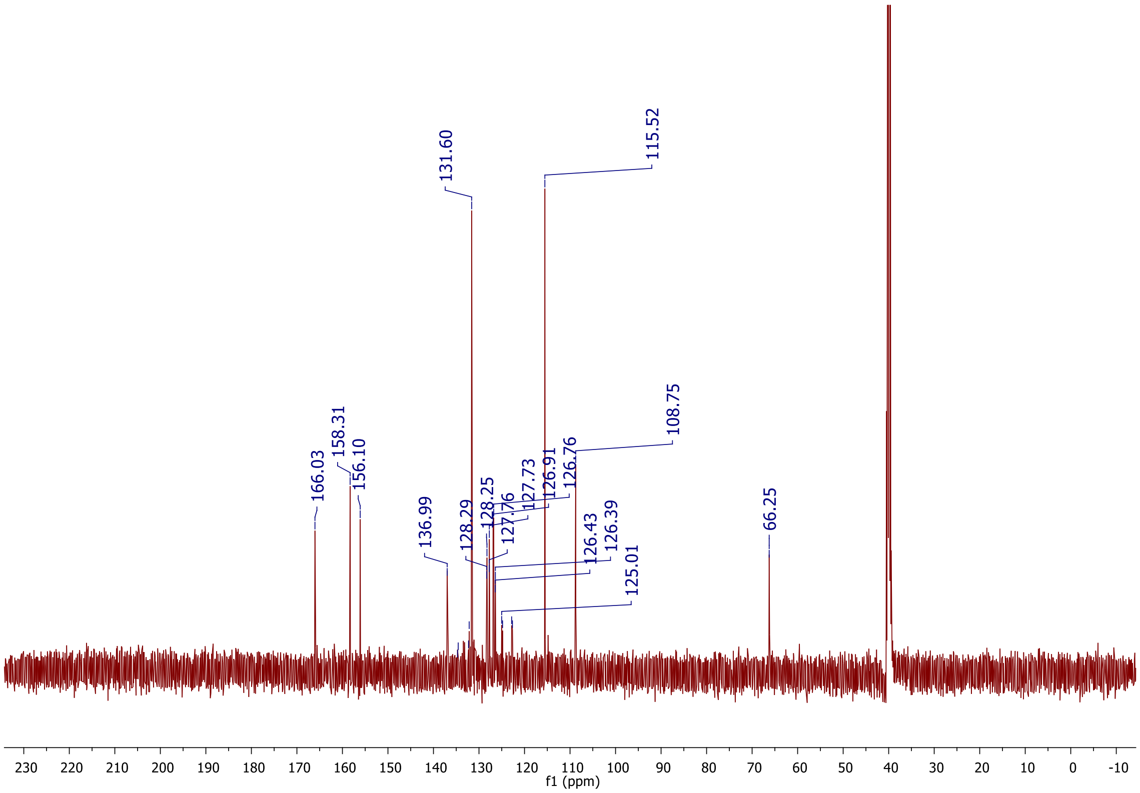

Figure SI 56 ^{13}CNMR 125 MHz of 31 in dimethyl sulfoxide-d_6.

(Z)-5-(4-((2,5-bis(trifluoromethyl)benzyl)oxy)-3-methoxybenzylidene)imidazolidine-2,4-dione (**32**)

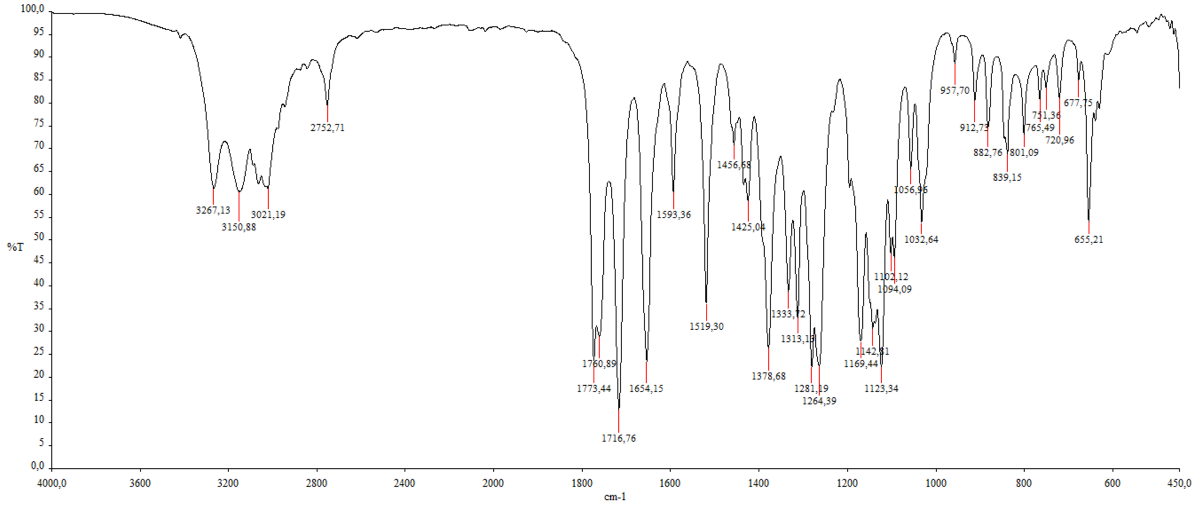

Figure SI 57 Infrared spectra of 32 in KBr pellets.

Elemental Composition Report **Page 1**

Single Mass Analysis
Tolerance = 10.0 PPM / DBE: min = -1.5, max = 50.0
Element prediction: Off
Number of isotope peaks used for i-FIT = 3

Monoisotopic Mass, Even Electron Ions
158 formula(e) evaluated with 1 results within limits (up to 3 best isotopic matches for each mass)
Elements Used:
C: 0-20 H: 0-30 N: 0-5 O: 0-5 F: 3-6

Naufal_biscf3ovhyd_neg 77 (1.289) Cm (76:77)
TOF MS ES-

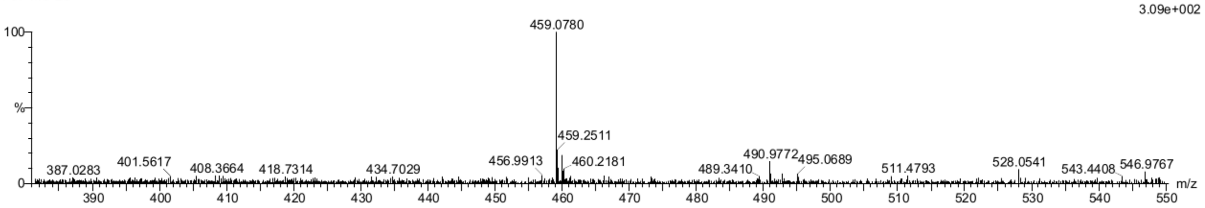

Figure SI 58 HR-TOF-MS-ES- spectra of 32.

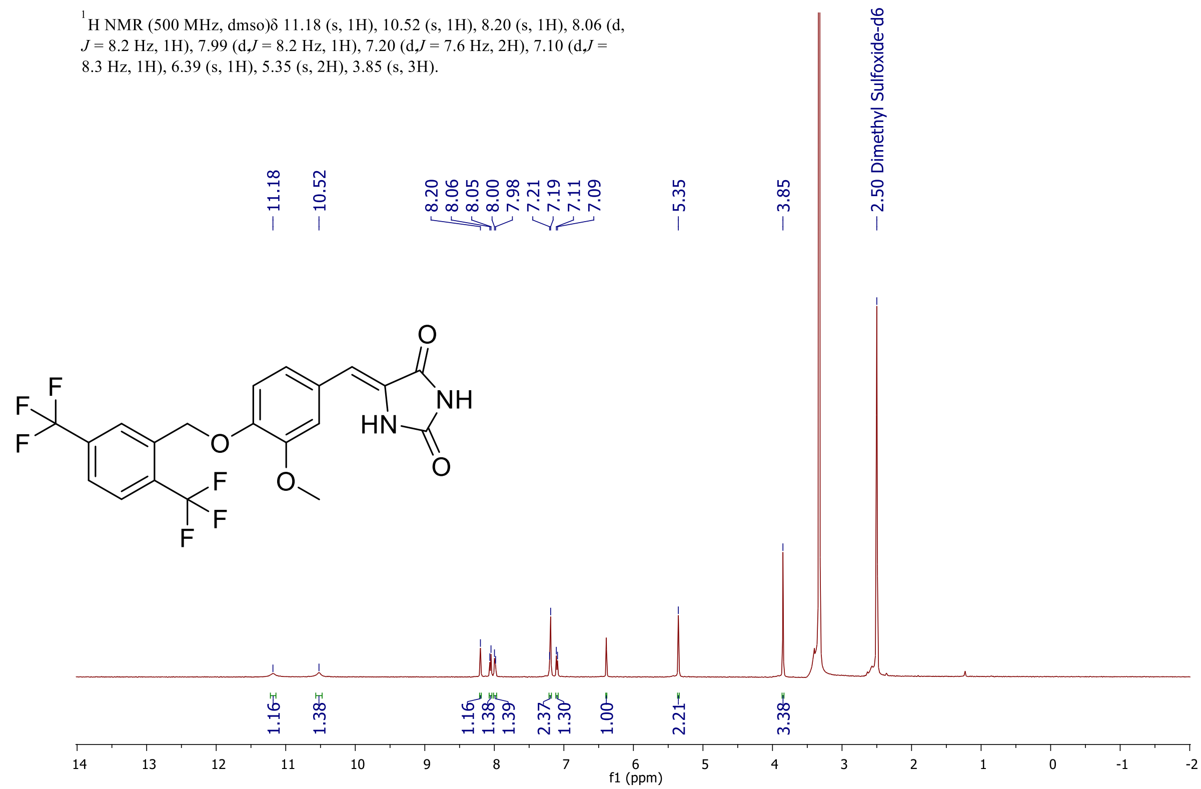

Figure SI 59 ^{1}H-NMR 500 MHz of 32 in dimethyl sulfoxide-d_6.

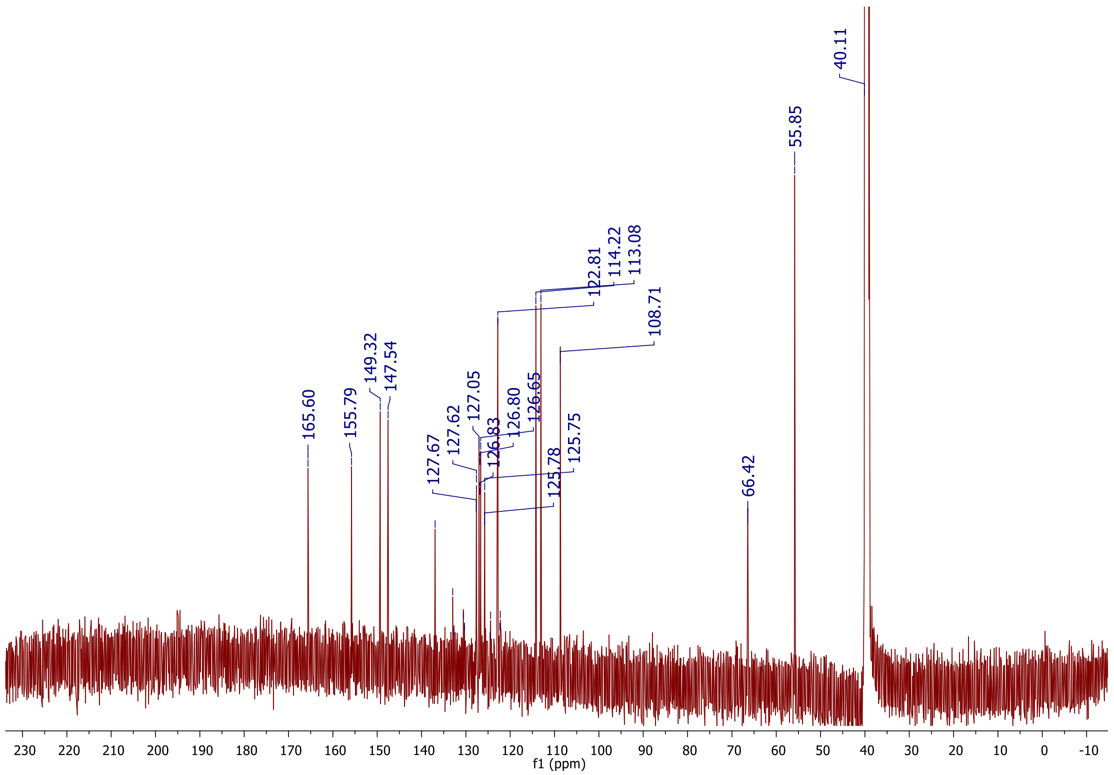

Figure SI 60 ^{13}CNMR 125 MHz of 32 in dimethyl sulfoxide-d_6.

(Z)-5-(3-((2,5-bis(trifluoromethyl)benzyl)oxy)-4-methoxybenzylidene)imidazolidine-2,4-dione (**33**)

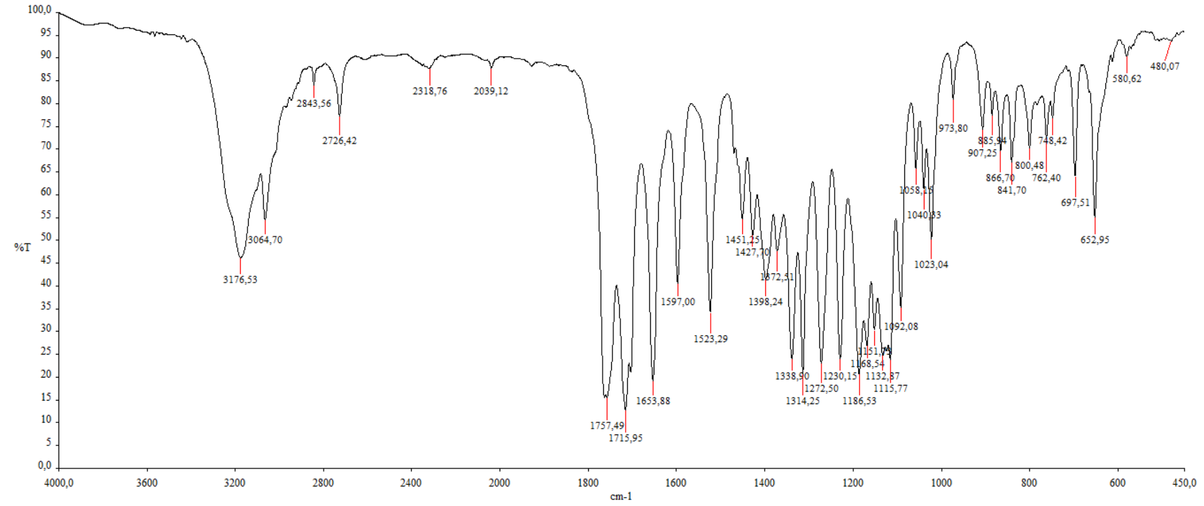

Figure SI 61 Infrared spectra of 33 in KBr pellets.

Elemental Composition Report **Page 1**

Single Mass Analysis
Tolerance = 10.0 PPM / DBE: min = -1.5, max = 50.0
Element prediction: Off
Number of isotope peaks used for i-FIT = 3

Monoisotopic Mass, Even Electron Ions
158 formula(e) evaluated with 1 results within limits (up to 3 best isotopic matches for each mass)
Elements Used:
C: 0-20 H: 0-30 N: 0-5 O: 0-5 F: 3-6

Naufal_biscf3oivhyd_neg 22 (0.356)
TOF MS ES-

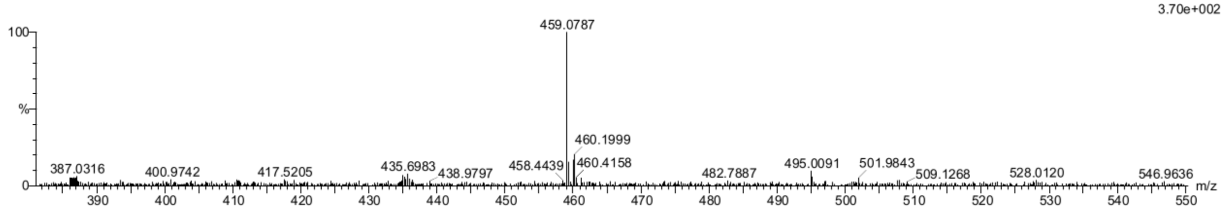

Figure SI 62 HR-TOF-MS-ES- spectra of 33.

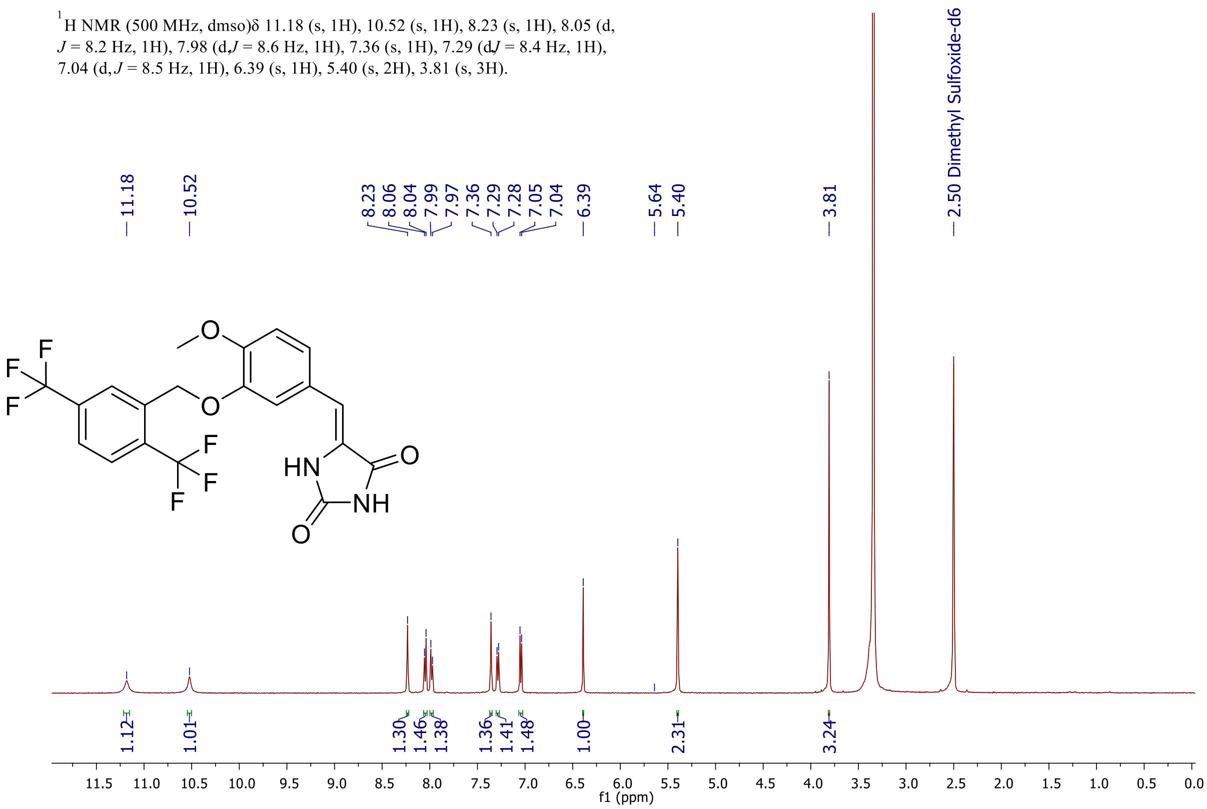

Figure SI 63 ^{1}H-NMR 500 MHz of 33 in dimethyl sulfoxide-d_6.

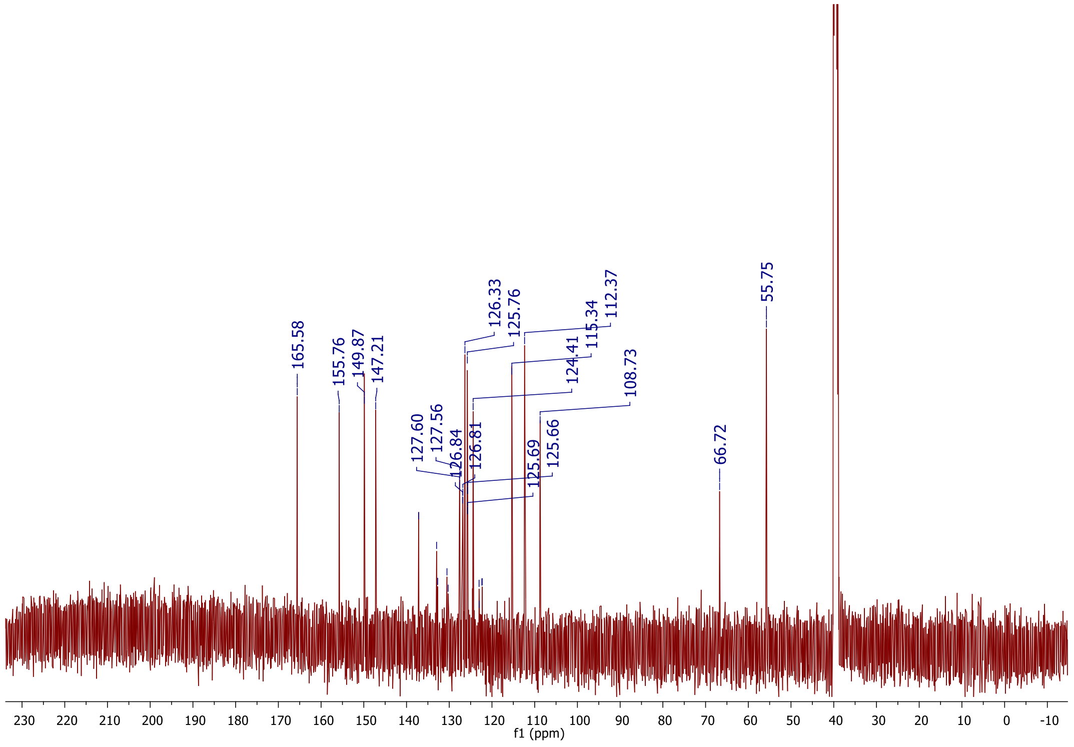

Figure SI 64 ^{13}CNMR 125 MHz of 33 in dimethyl sulfoxide-d_6.

(Z)-5-(4-((2,5-bis(trifluoromethyl)benzyl)oxy)-3-ethoxybenzylidene)imidazolidine-2,4-dione (**34**)

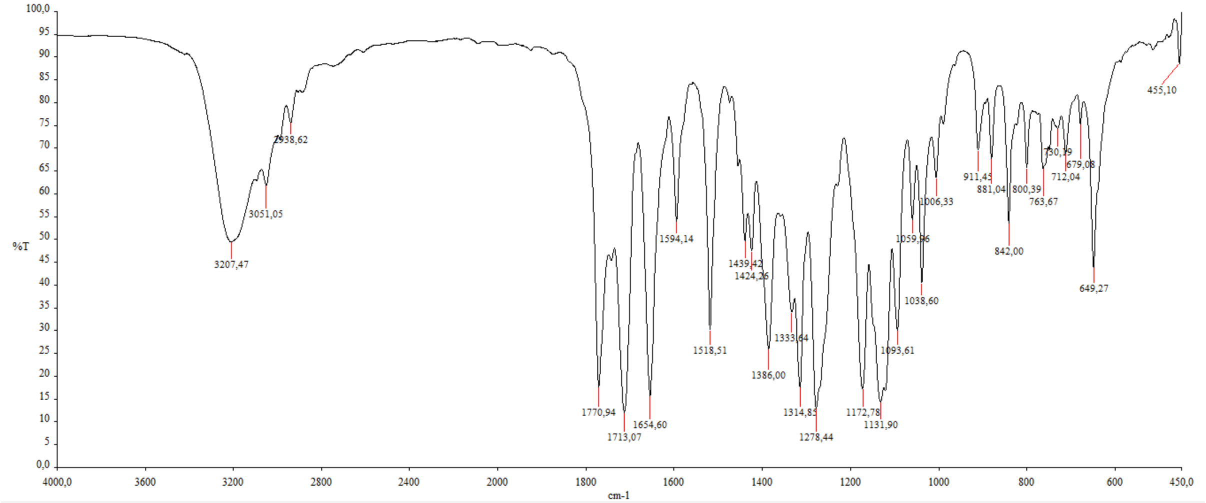

Figure SI 65 Infrared spectra of 34 in KBr pellets.

Elemental Composition Report **Page 1**

Single Mass Analysis
Tolerance = 10.0 PPM / DBE: min = -1.5, max = 50.0
Element prediction: Off
Number of isotope peaks used for i-FIT = 3

Monoisotopic Mass, Even Electron Ions
152 formula(e) evaluated with 1 results within limits (up to 3 best isotopic matches for each mass)
Elements Used:
C: 21-23 H: 0-30 N: 0-5 O: 0-5 F: 3-6

Naufal_biscf3oevhyd_neg 10 (0.153)
TOF MS ES-

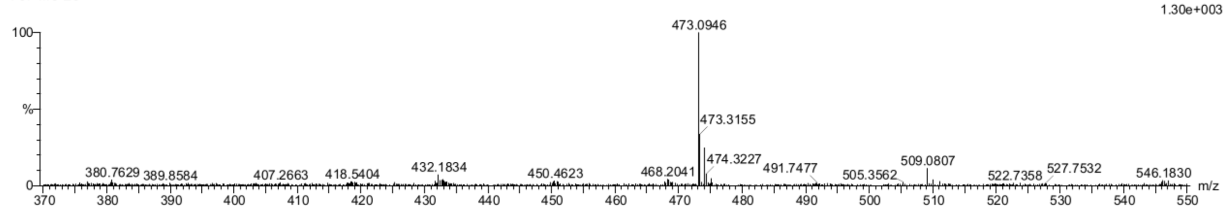

Figure SI 66 HR-TOF-MS-ES- spectra of 34.

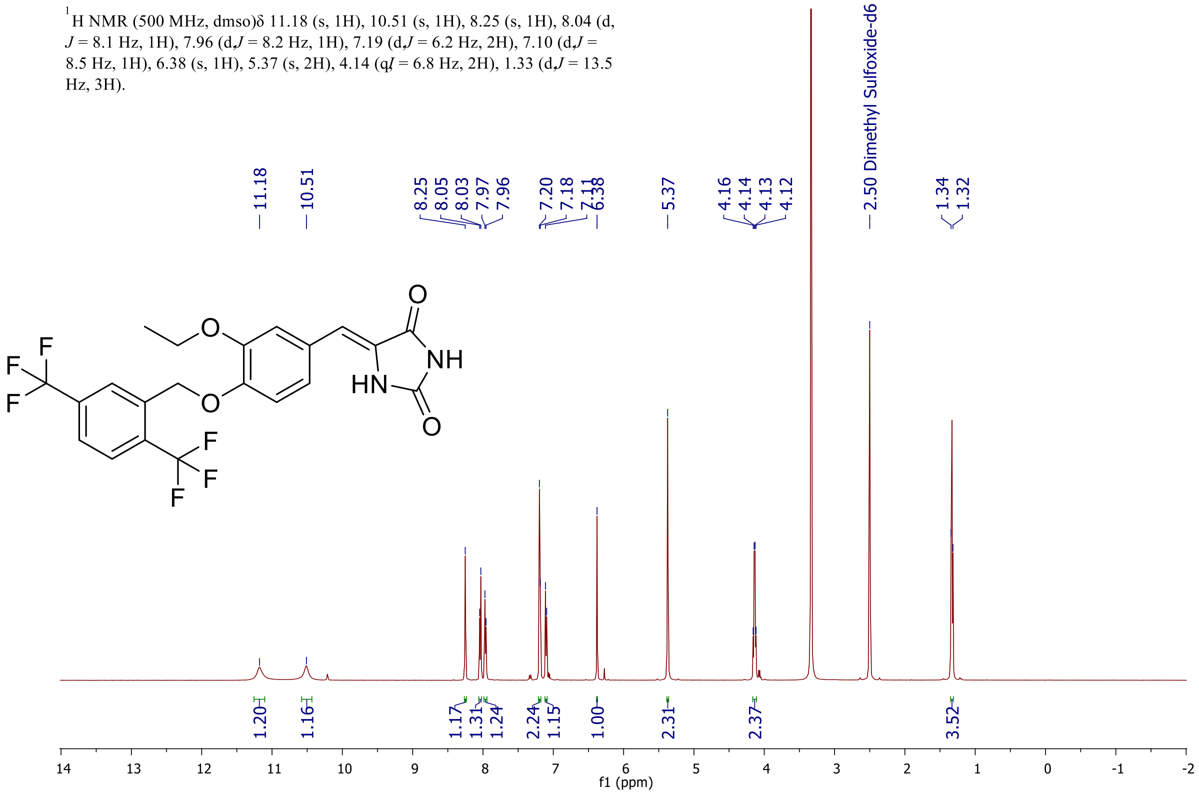

Figure SI 67 ^{1}H-NMR 500 MHz of 34 in dimethyl sulfoxide-d_6.

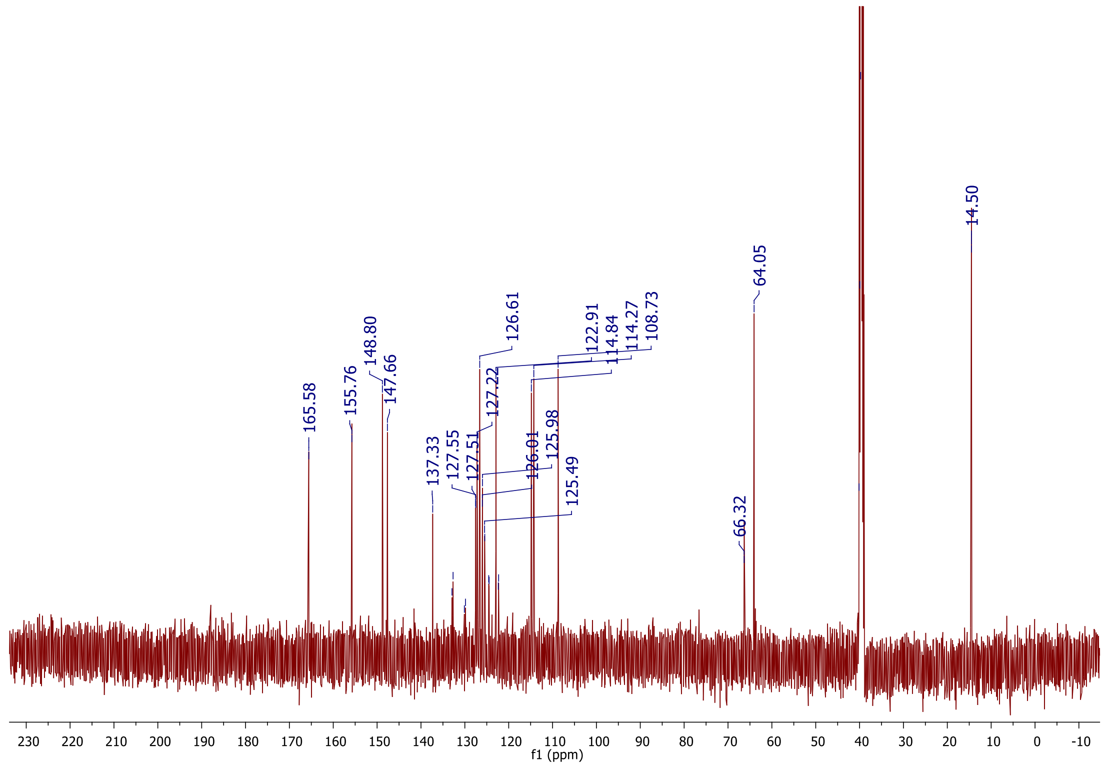

Figure SI 68 ^{13}CNMR 125 MHz of 34 in dimethyl sulfoxide-d_6.

(Z)-5-(2-chloro-5-(trifluoromethyl)benzylidene)imidazolidine-2,4-dione (**35**)

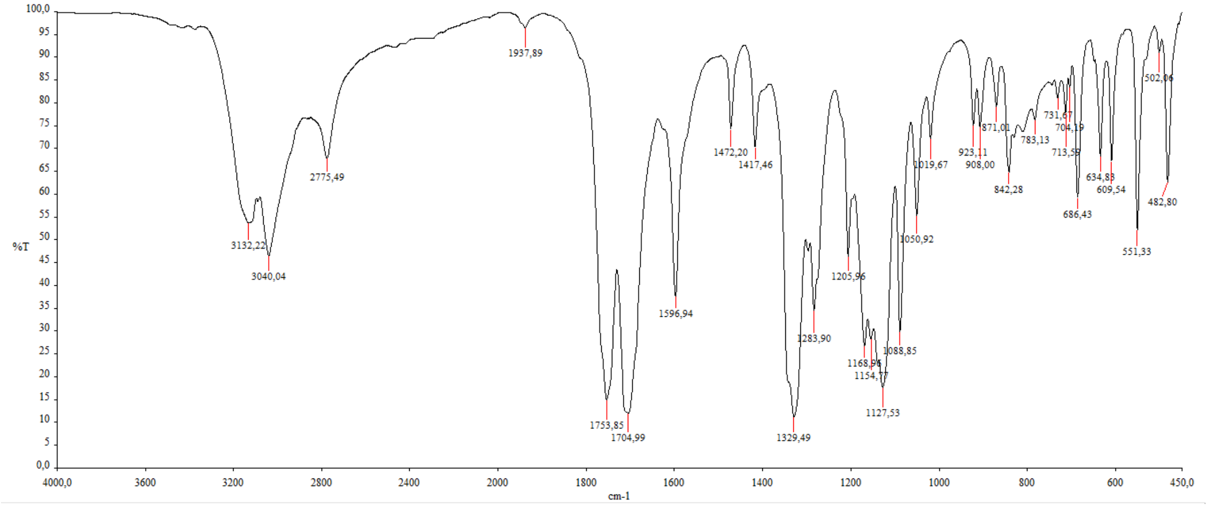

Figure SI 69 Infrared spectra of 35 in KBr pellets.

Elemental Composition Report **Page 1**

Single Mass Analysis
Tolerance = 10.0 PPM / DBE: min = -1.5, max = 50.0
Element prediction: Off
Number of isotope peaks used for i-FIT = 3

Monoisotopic Mass, Even Electron Ions
188 formula(e) evaluated with 1 results within limits (up to 3 best isotopic matches for each mass)
Elements Used:
C: 11-11 H: 0-30 N: 0-4 O: 0-4 Cl: 0-1 F: 0-3
Naufal_N2K5TFM_Neg 47 (0.780)
TOF MS ES-

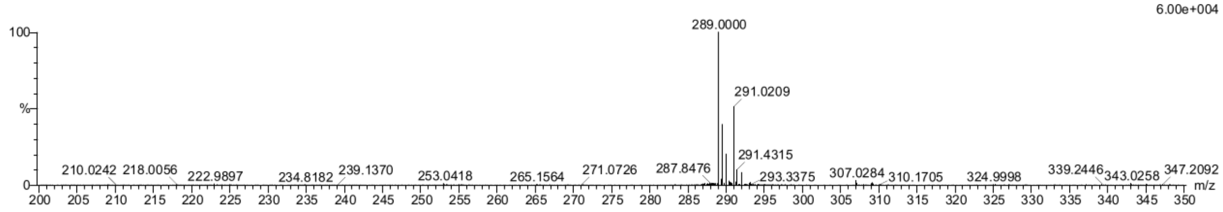

Figure SI 70 HR-TOF-MS-ES- spectra of 35.

^{1}H NMR (500 MHz, dmso)δ 11.21 (s, 2H), 7.89 (s, 1H), 7.73 (d,J = 8.4 Hz, 1H), 7.66 (d,J = 8.2 Hz, 1H), 6.47 (s, 1H).

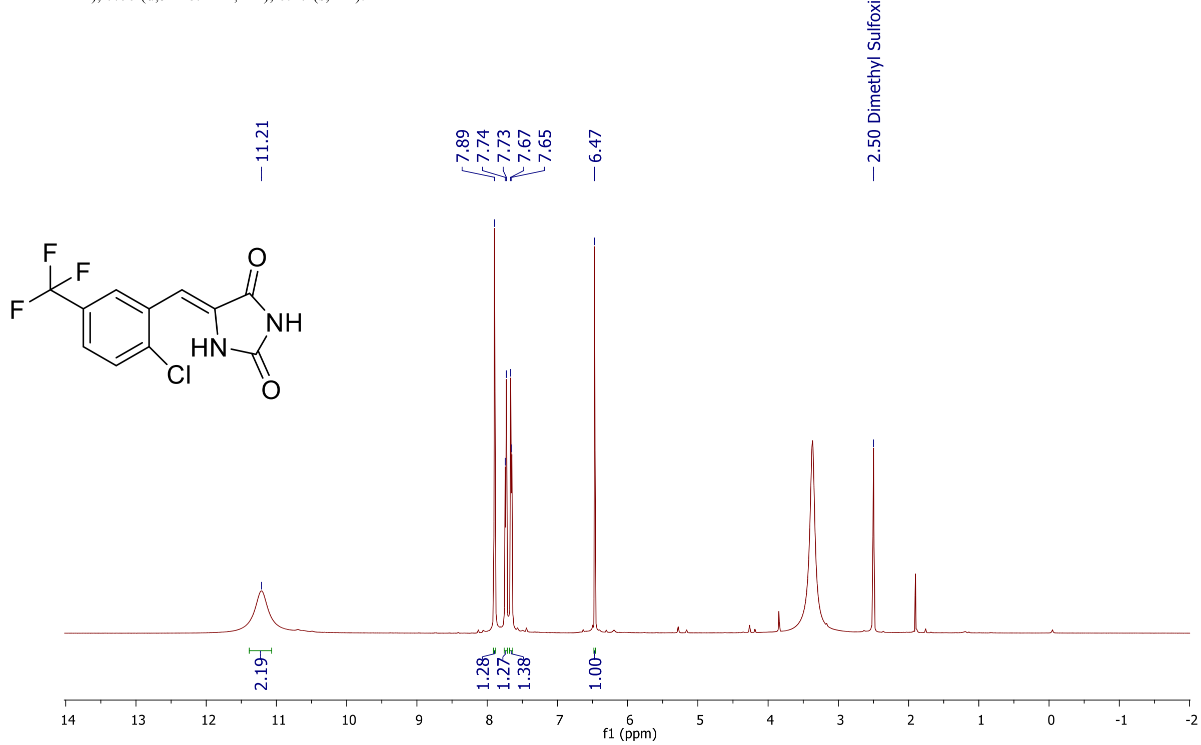

Figure SI 71. ^{1}H-NMR 500 MHz of 35 in dimethyl sulfoxide-d_6.

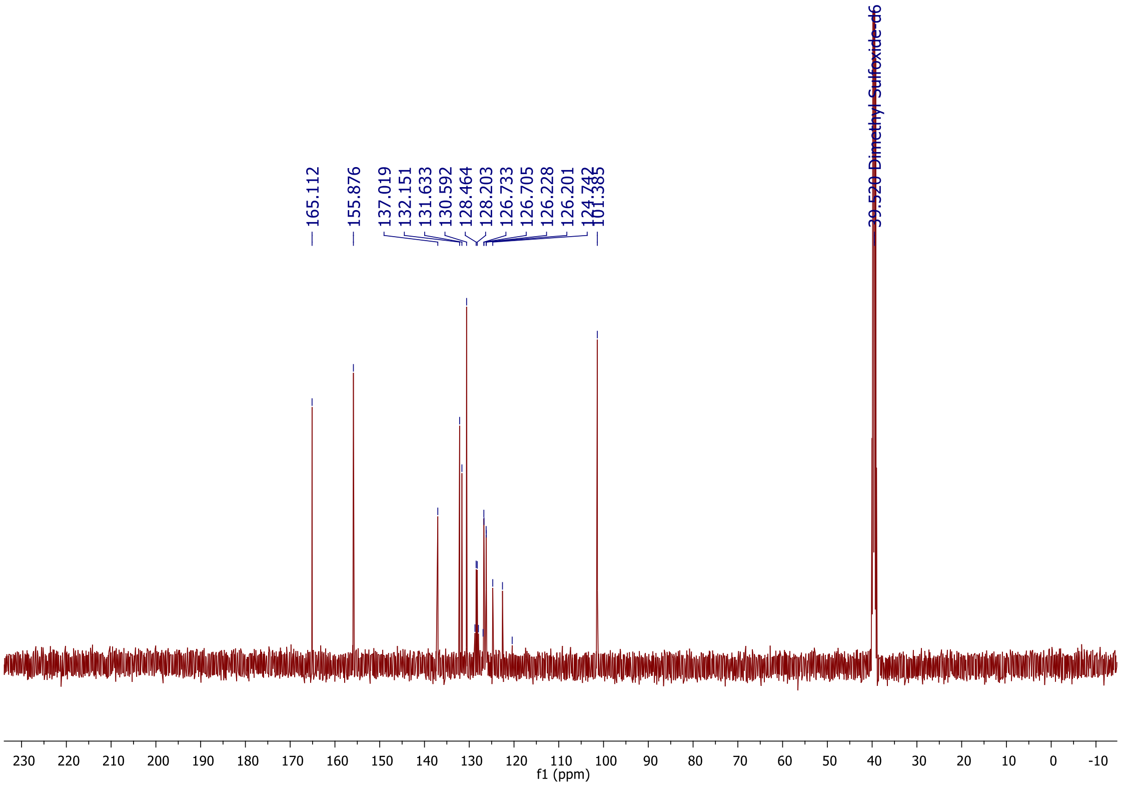

Figure SI 72 ^{13}CNMR 125 MHz of 35 in dimethyl sulfoxide-d_6.

Z)-5-(2-bromo-4-hydroxy-5-methoxybenzylidene)imidazolidine-2,4-dione (**36**)

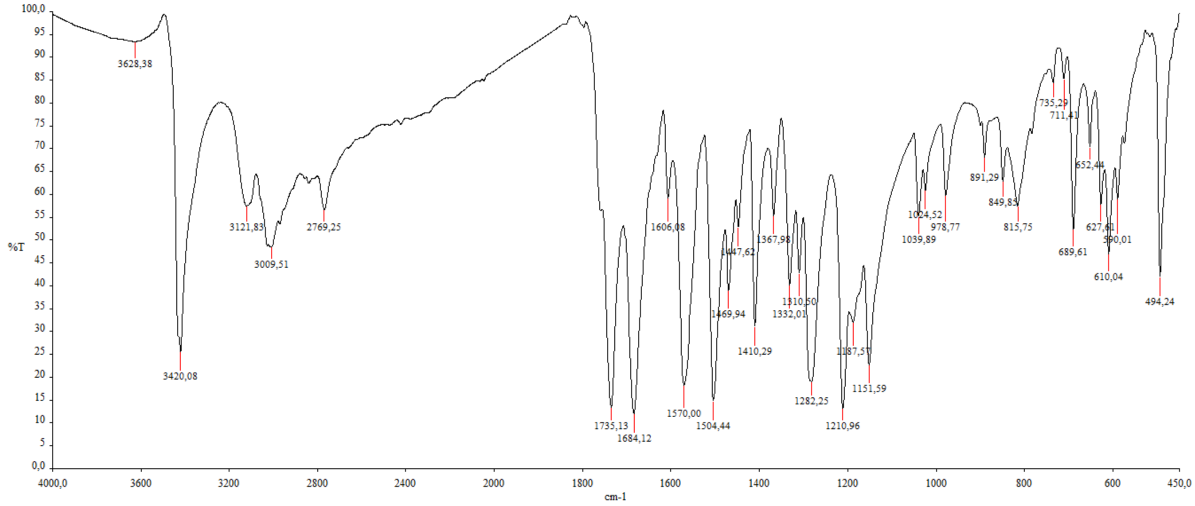

Figure SI 73 Infrared spectra of 36 in KBr pellets.

Elemental Composition Report **Page 1**

Single Mass Analysis
Tolerance = 10.0 PPM / DBE: min = -1.5, max = 50.0
Element prediction: Off
Number of isotope peaks used for i-FIT = 3

Monoisotopic Mass, Even Electron Ions
96 formula(e) evaluated with 1 results within limits (up to 3 best isotopic matches for each mass)
Elements Used:
C: 0-30 H: 0-30 N: 0-5 O: 0-5 Br: 1-1
Naufal_N2B4O5N-01_neg 36 (0.593)
TOF MS ES-

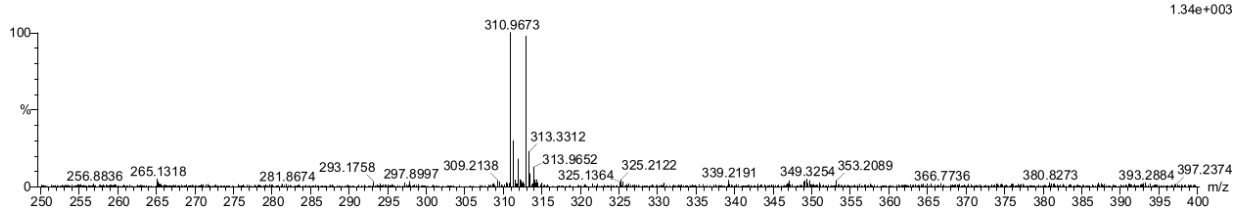

Figure SI 74 HR-TOF-MS-ES- spectra of 36.

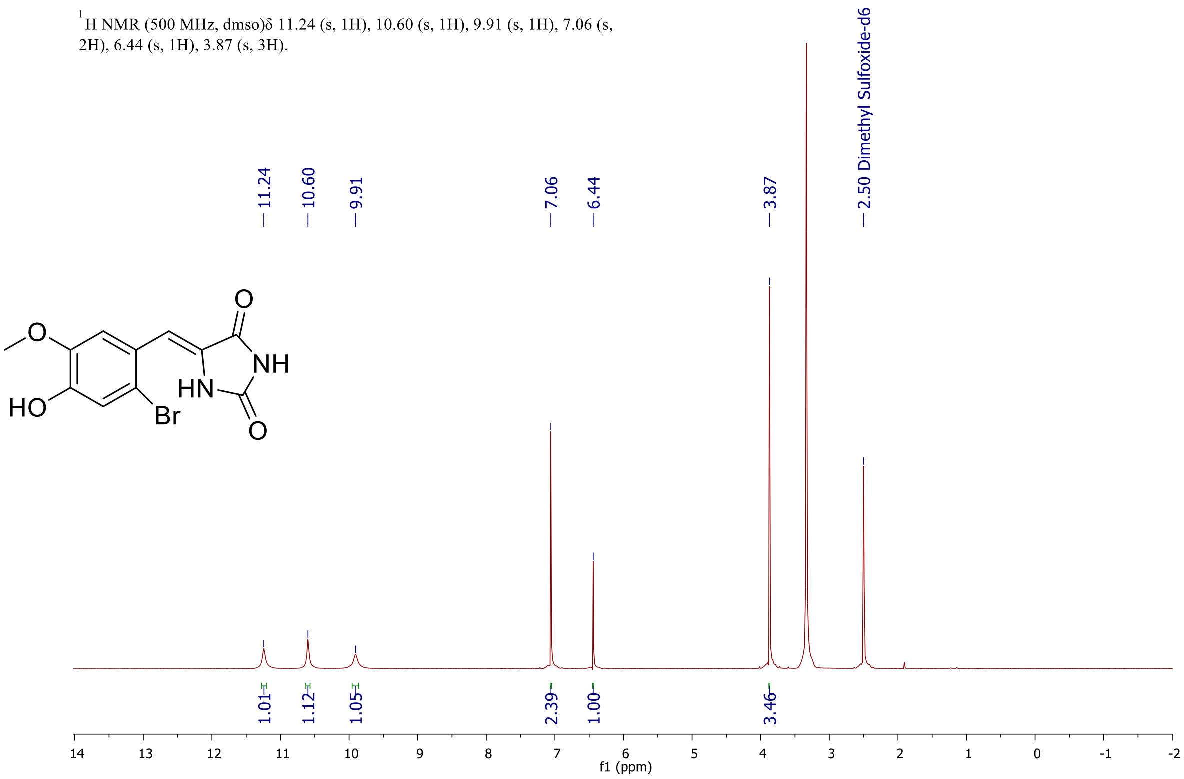

Figure SI 75 ^{1}H-NMR 500 MHz of 36 in dimethyl sulfoxide-d_6.

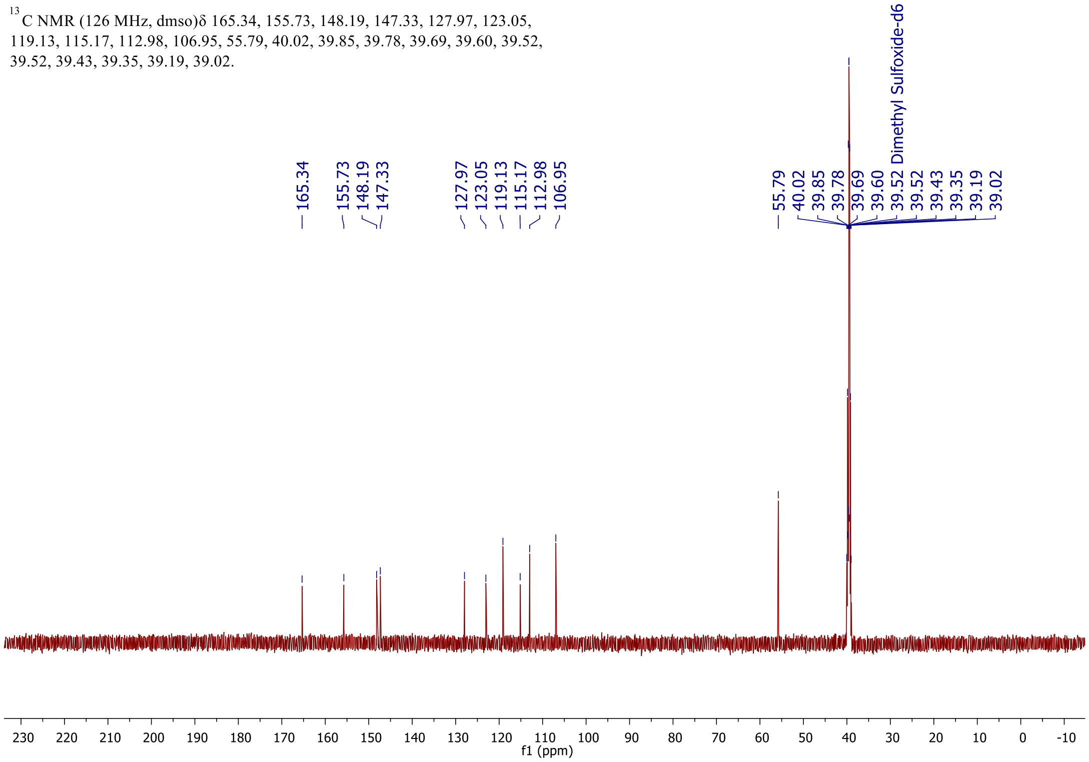

Figure SI 76 ^{13}CNMR 125 MHz of 36 in dimethyl sulfoxide-d_6.

(Z)-2-(4-((2,5-dioxoimidazolidin-4-ylidene)methyl)-2-methoxyphenoxy)acetic acid (**37**)

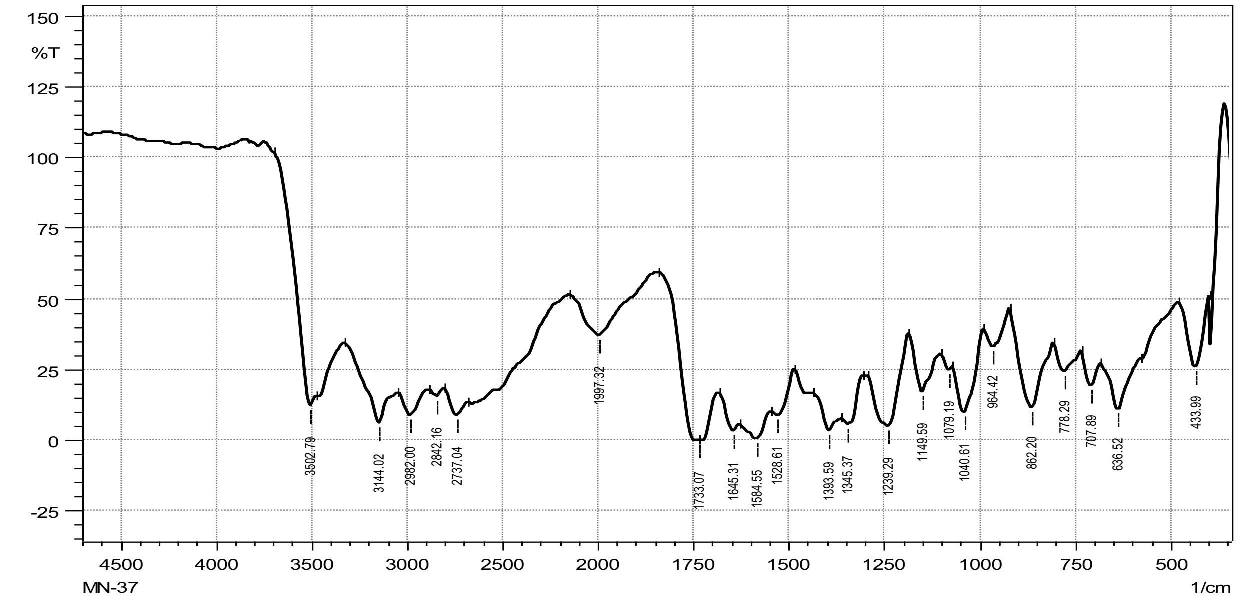

Figure SI 77. Infrared spectra of 37 in KBr pellets.

Elemental Composition Report **Page 1**

Single Mass Analysis
Tolerance = 10.0 PPM / DBE: min = -1.5, max = 50.0
Element prediction: Off
Number of isotope peaks used for i-FIT = 3

Monoisotopic Mass, Even Electron Ions
139 formula(e) evaluated with 1 results within limits (up to 3 best isotopic matches for each mass)
Elements Used:
C: 0-35 H: 0-50 N: 1-3 O: 0-15
Naufal_cek reaksi_neg 10 (0.153)
TOF MS ES-

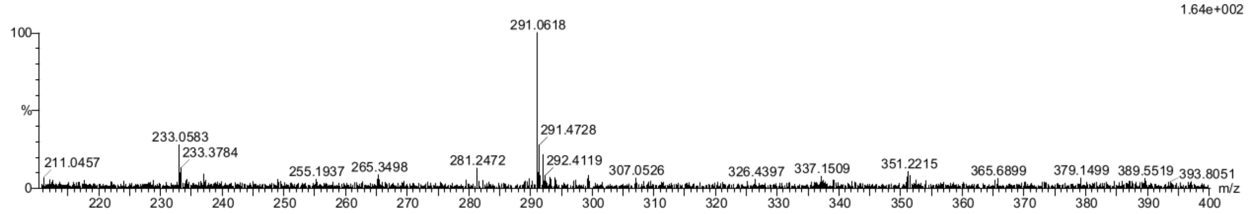

Figure SI 78 HR-TOF-MS-ES- spectra of 37.

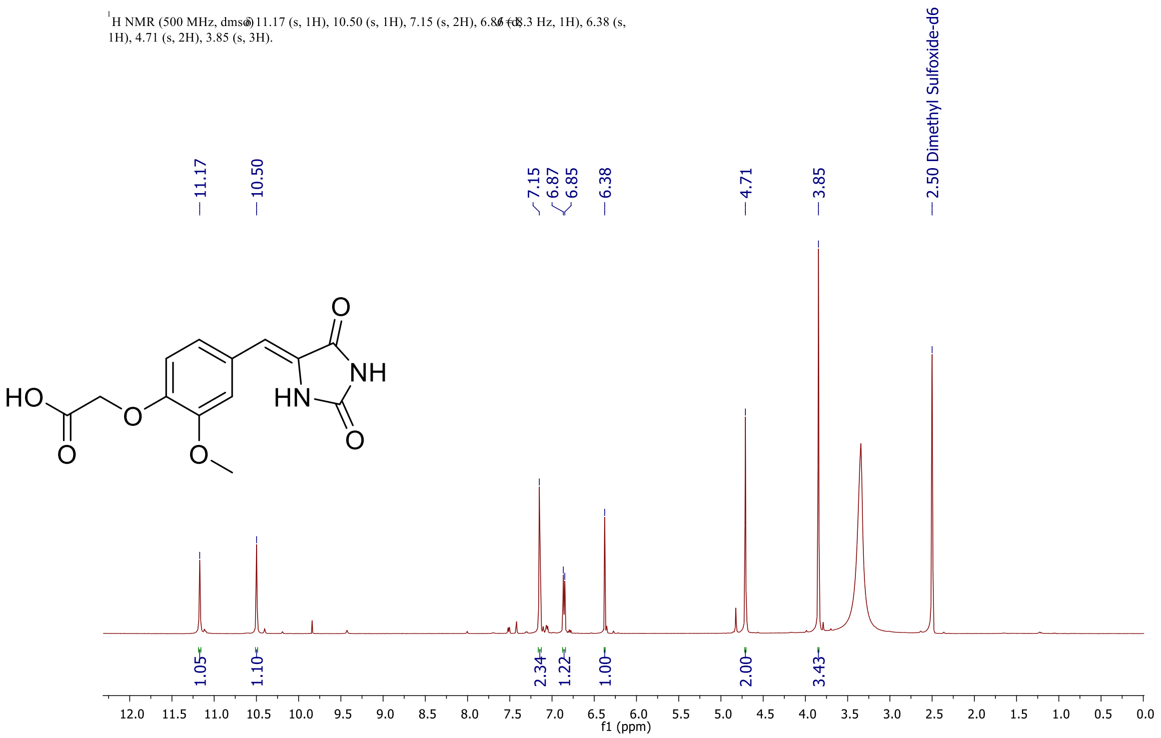

Figure SI 79 ^{1}H-NMR 500 MHz of 37 in dimethyl sulfoxide-d_6.

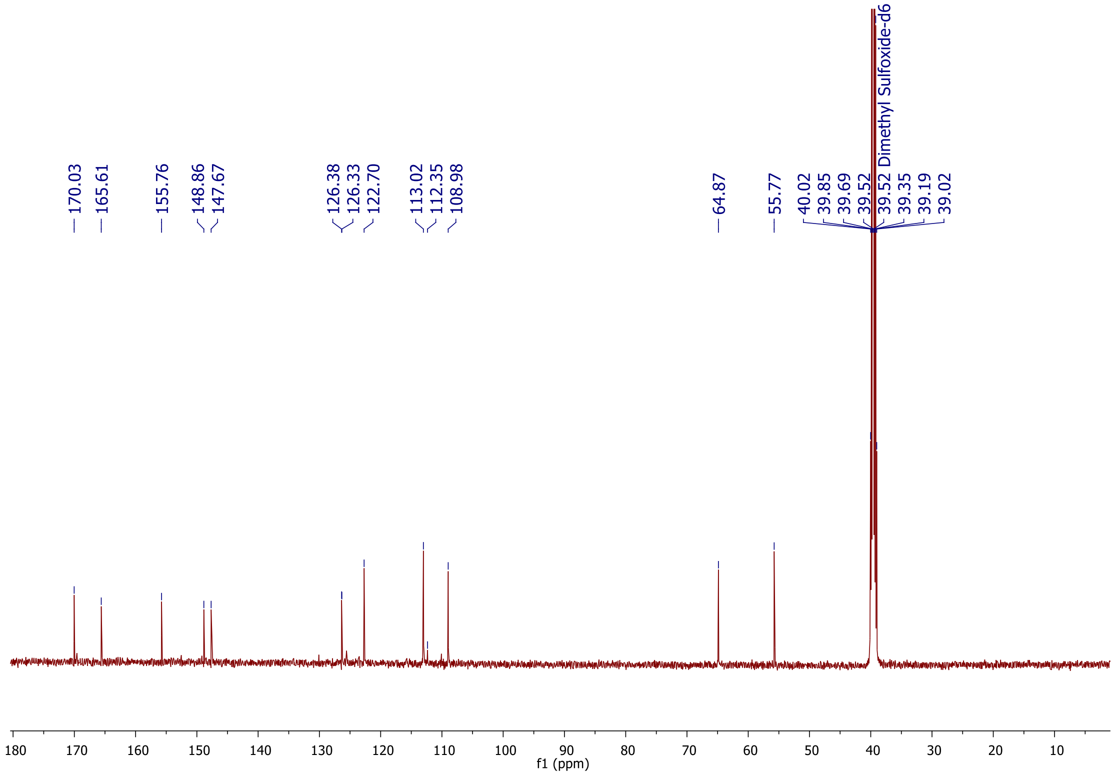

Figure SI 80 ^{13}CNMR 125 MHz of 37 in dimethyl sulfoxide-d_6.

ethyl (Z)-5-((2,5-dioxoimidazolidin-4-ylidene)methyl)-2,4-dimethyl-1H-pyrrole-3-carboxylate **38**

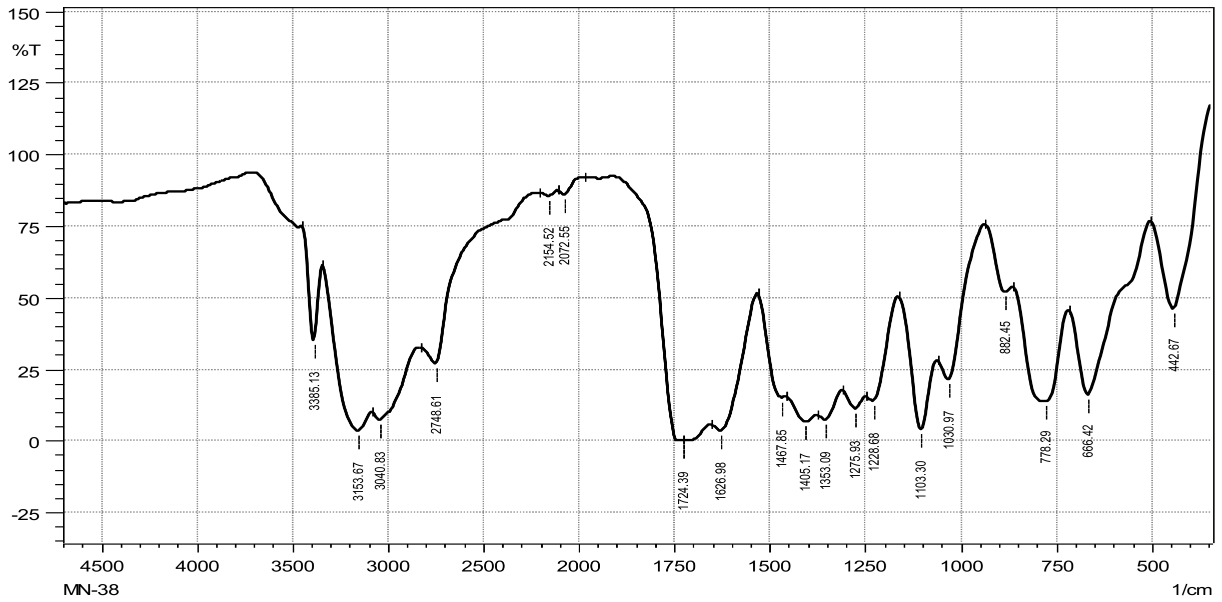

Figure SI 81 Infrared spectra of 38 in KBr pellets.

Elemental Composition Report **Page 1**

Single Mass Analysis
Tolerance = 10.0 PPM / DBE: min = -1.5, max = 50.0
Element prediction: Off
Number of isotope peaks used for i-FIT = 3

Monoisotopic Mass, Even Electron Ions
62 formula(e) evaluated with 1 results within limits (up to 3 closest results for each mass)
Elements Used:
C: 0-20 H: 0-20 N: 0-5 O: 0-5
Naufal_HYD_Py_CooET_neg 6 (0.085)
TOF MS ES-

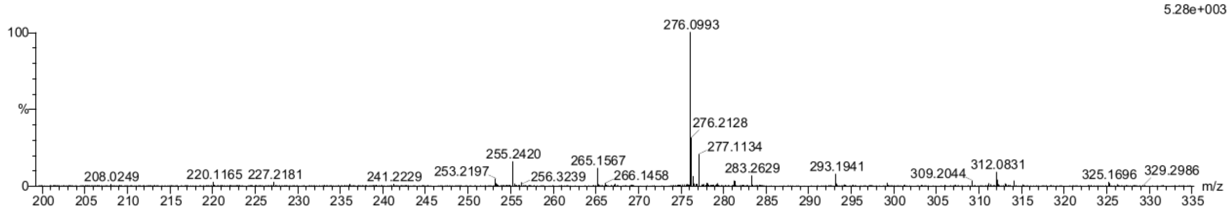

Figure SI 82 Infrared spectra of 38 in KBr pellets.

^{1}H NMR (500 MHz, dmso) δ 12.24 (s, 1H), 11.38 (s, 1H), 10.09 (s, 1H), 6.31 (s, 1H), 4.17 (dd, J = 14.1, 7.0 Hz, 2H), 2.45 (s, 3H), 2.23 (s, 3H), 1.27 (t, J = 7.0 Hz, 3H).

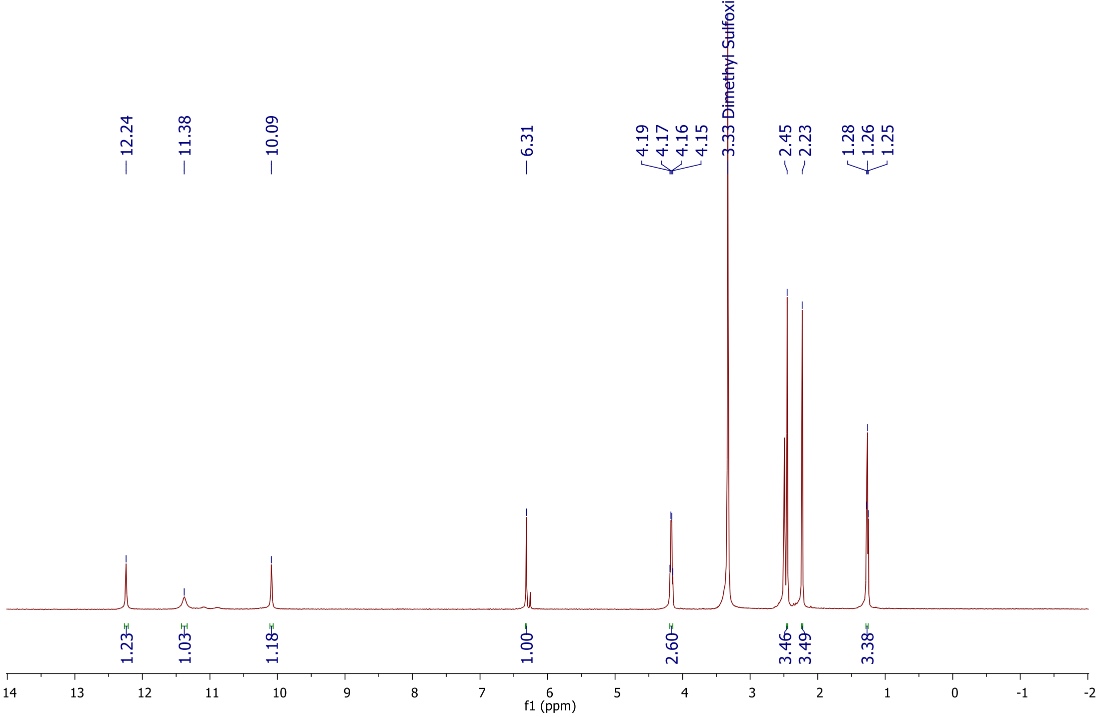

Figure SI 83 ^{1}H-NMR 500 MHz of 38 in dimethyl sulfoxide-d_6.

^{13}C NMR (126 MHz, dmso) δ 165.78, 164.54, 152.95, 137.10, 125.31, 122.93, 112.06, 104.95, 97.39, 58.89, 40.02, 39.85, 39.69, 39.52, 39.52, 39.35, 39.19, 39.02, 14.30, 14.16, 10.96.

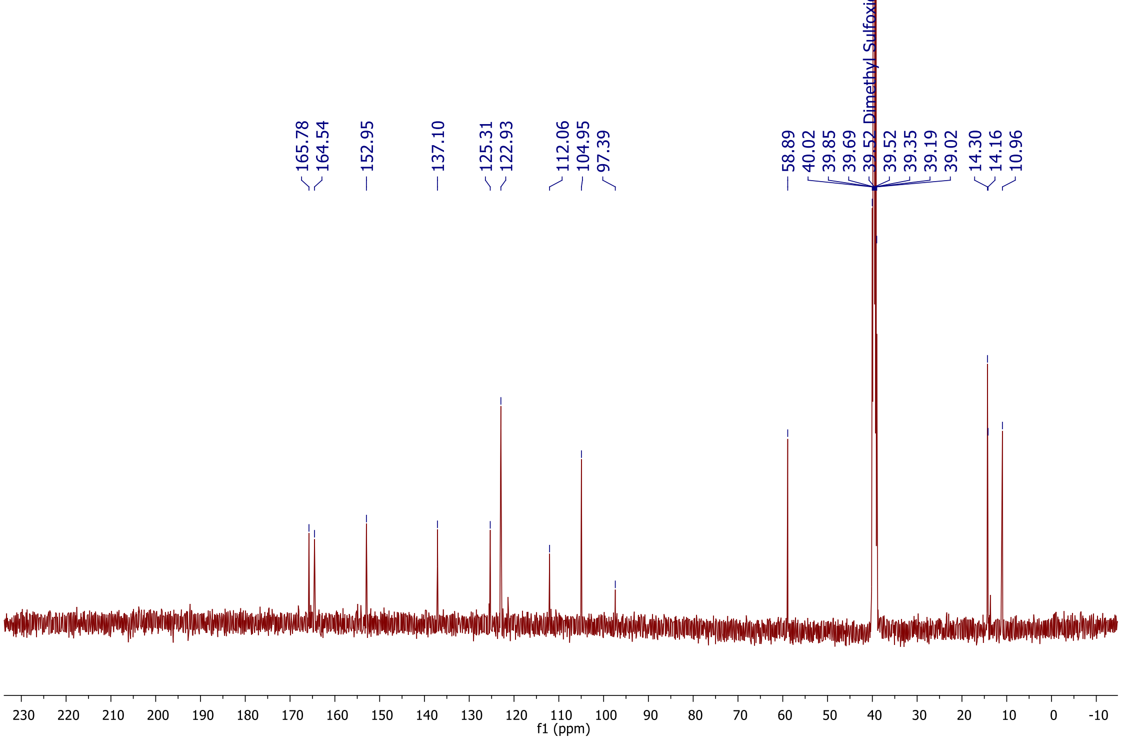

Figure SI 84 ^{13}CNMR 125 MHz of 38 in dimethyl sulfoxide-d_6.

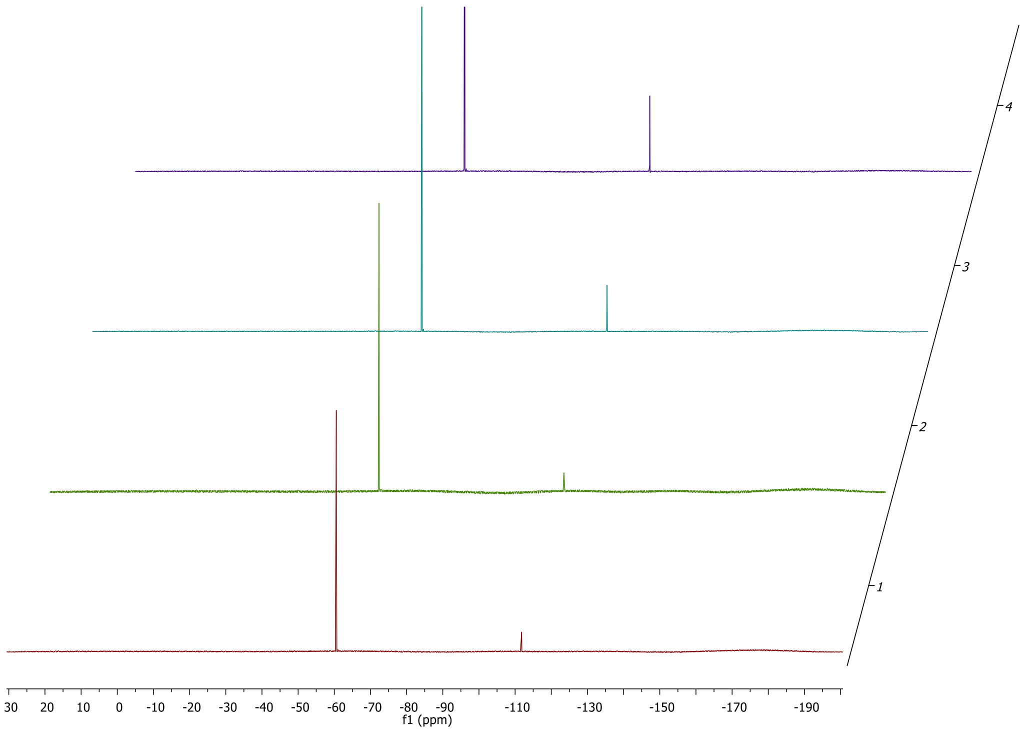

Figure SI 85 ^{19}FNMR spectra of 27-30.

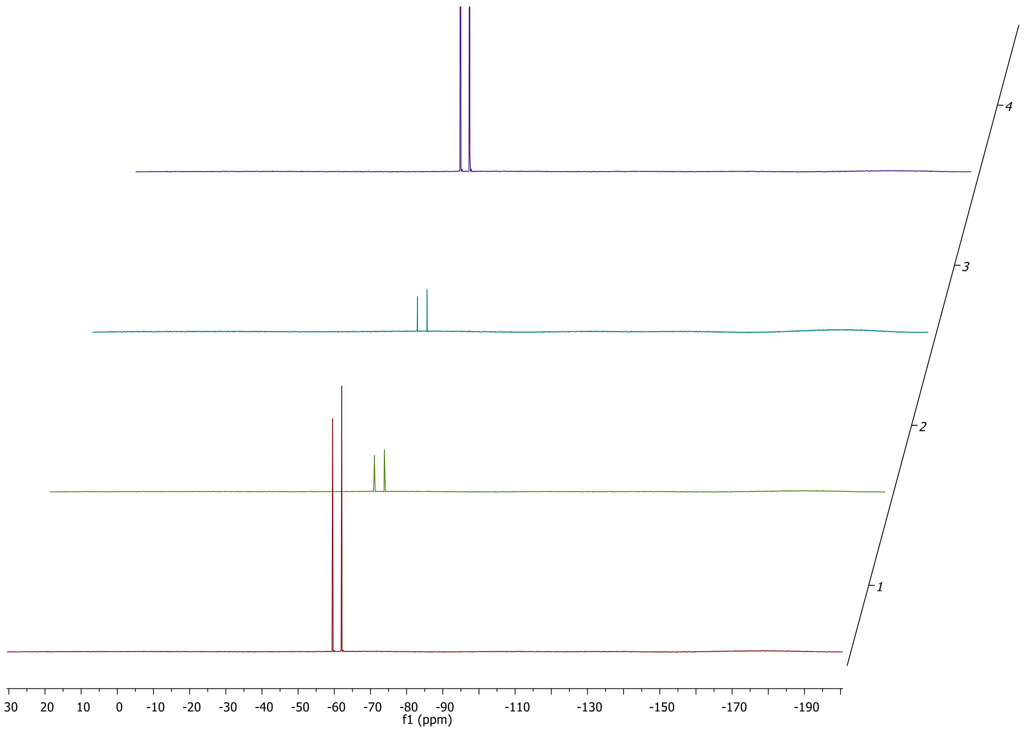

Figure SI 86 ^{19}FNMR spectra of 31-34.
